# Supplementary material for: Intra- and interchromosomal contact mapping reveals the Igh locus has extensive conformational heterogeneity and interacts with B-lineage genes
Source: Cell Rep. 2023 Sep 6;42(9):113074. doi: 10.1016/j.celrep.2023.113074 (PMC10548092; doi:10.1016/j.celrep.2023.113074)
Supplement: Document S1. Figures S1–S7 [file mmc1.pdf]

Cell Reports, Volume *42*

Supplemental information

Intra- and interchromosomal contact mapping reveals the *Igh* locus has extensive conformational heterogeneity and interacts with B-lineage genes

Olga Mielczarek, Carolyn H. Rogers, Yinxiu Zhan, Louise S. Matheson, Michael J.T. Stubbington, Stefan Schoenfelder, Daniel J. Bolland, Biola M. Javierre, Steven W. Wingett, Csilla Várnai, Anne Segonds-Pichon, Simon J. Conn, Felix Krueger, Simon Andrews, Peter Fraser, Luca Giorgetti, and Anne E. Corcoran

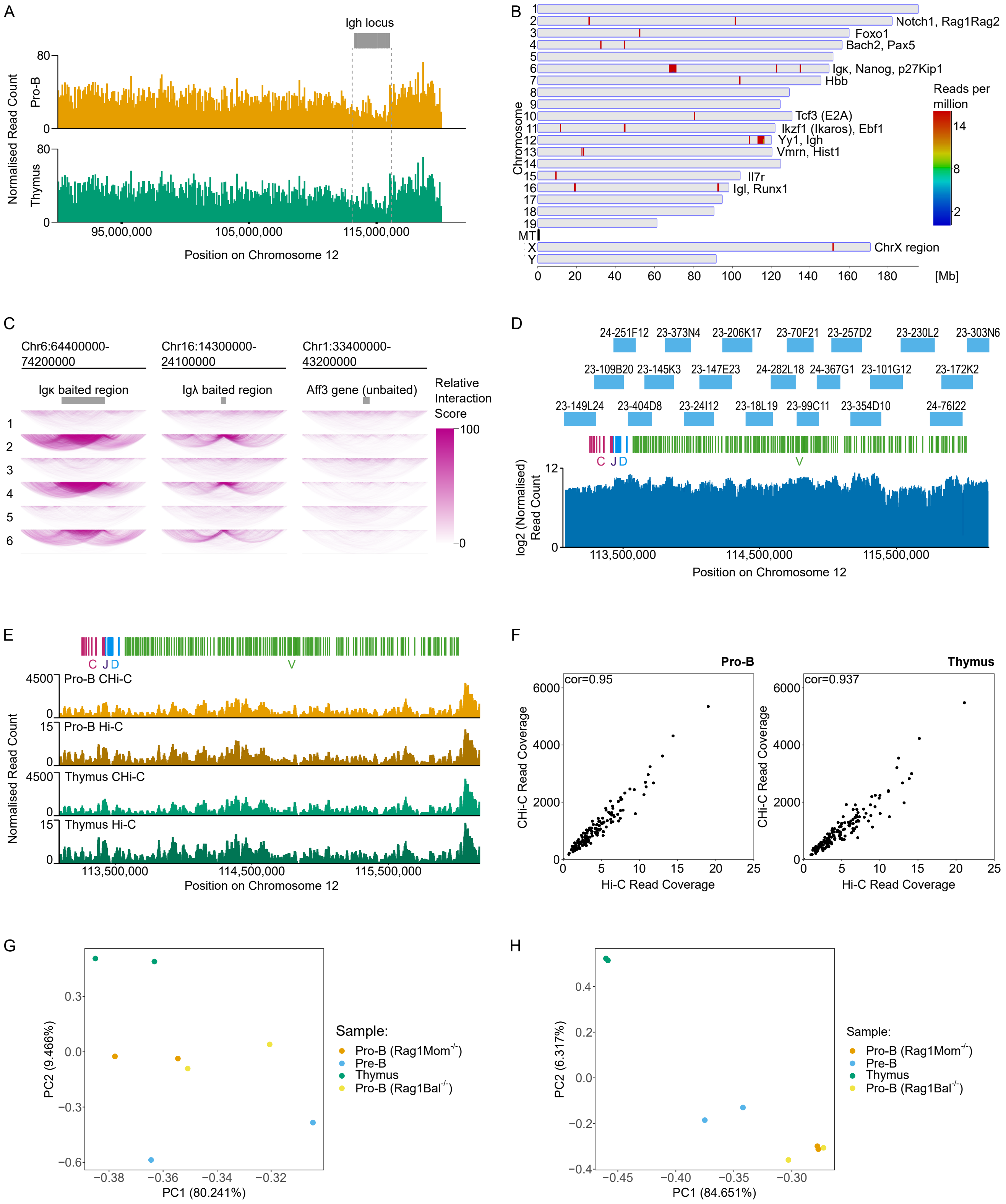
A
Igh locus
Normalised Read Count
Pro-B
Thymus
95,000,000
105,000,000
115,000,000
Position on Chromosome 12
B
Chromosome
Notch1, Rag1Rag2
Foxo1
Bach2, Pax5
Igκ, Nanog, p27Kip1
Hbb
Tcf3 (E2A)
Ikzf1 (Ikaros), Ebf1
Yy1, Igh
Vmrn, Hist1
Il7r
Igl, Runx1
ChrX region
Reads per million
[Mb]
C
Chr6:64400000-74200000
Chr16:14300000-24100000
Chr1:33400000-43200000
Igκ baited region
Igλ baited region
Aff3 gene (unbaited)
Relative Interaction Score
D
24-251F12 23-373N4 23-206K17 23-70F21 23-257D2 23-230L2 23-303N6
23-109B20 23-145K3 23-147E23 24-282L18 24-367G1 23-101G12 23-172K2
23-149L24 23-404D8 23-24I12 23-18L19 23-99C11 23-354D10 24-76I22
C J D
V
log2 (Normalised) Read Count
113,500,000
114,500,000
115,500,000
Position on Chromosome 12
E
C J D
V
Normalised Read Count
Pro-B CHi-C
Pro-B Hi-C
Thymus CHi-C
Thymus Hi-C
113,500,000
114,500,000
115,500,000
Position on Chromosome 12
F
Pro-B
Thymus
cor=0.95
cor=0.937
CHi-C Read Coverage
Hi-C Read Coverage
G
PC2 (9.466%)
PC1 (80.241%)
Sample:
Pro-B (Rag1Mom-/-)
Pre-B
Thymus
Pro-B (Rag1Bal-/-)
H
PC2 (6.317%)
PC1 (84.651%)
Sample:
Pro-B (Rag1Mom-/-)
Pre-B
Thymus
Pro-B (Rag1Bal-/-)

Figure S1. Enrichment of Hi-C material by bait pull-down. Related to Figure 1

(A) The Igh has lower read coverage in Hi-C than adjacent regions. Hi-C reads in the region chr12:90,000,000-end were quantitated in 100kb bins, normalised per million reads and biological replicates were averaged.

(B) The baits were validated by RNA-seq. Read count per million in 100bp bins is shown genome-wide.

(C) Interactions of the baited Igκ and Igλ loci and non-baited Aff3 locus in Hi-C and CHi-C datasets. 5 million randomly sampled reads from HiCUP-processed Hi-C and CHi-C datasets were quantified in 200kb bins and visualized in the WashU Epigenome Browser. Arcs show interactions. Arc colour indicates the number of interactions. One representative replicate is shown for each type of dataset: 1 – Hi-C Pro-B 1; 2 – CHi-C Pro-B 1; 3 – Hi-C Pre-B 1; 4 – CHi-C Pre-B 1; 5 – Hi-C Thymus 1; 6 – CHi-C Thymus 1.

(D) Validation of Igh baits by RNA-seq. Log2 read count per million over HindIII sites (-300bp to +300bp) for the Igh baited region (C) are shown. BAC positions are indicated by the light blue rectangles.

(E) CHi-C and Hi-C libraries exhibit the same read coverage pattern across the Igh locus. For pro-B and thymus samples, reads in Hi-C libraries and Scribler-processed CHi-C libraries were quantitated in 10kb bins with 1kb step, normalised per million reads. Biological replicates were averaged.

(F) CHi-C and Hi-C read coverage across the Igh locus is correlated. For pro-B and thymus samples, reads in Hi-C libraries and Scribler-processed CHi-C libraries were quantitated in 20kb windows over the Igh locus, normalised per million reads and biological replicates averaged. cor = spearman correlation coefficients.

(G) PCA shows reproducibility of biological replicates. Scribler-processed CHi-C datasets screened against a blacklist were processed in Homer to generate genome wide interaction matrices at 0.5Mb resolution, correcting for read coverage. The PCA examined normalised interaction frequencies across all replicates for all pairs of 0.5Mb bins genome wide, filtering out pairs of bins where the normalised interaction frequency was zero across all replicates.

(H) PCA shows that all pro-B cell replicates have similar Igh locus conformation.

Homer read coverage corrected interaction matrices at 20kb resolution were generated for the Igh baited region for all replicates. Values corresponding to 'white lines,' where normalised interaction frequency could not be computed, were filtered from the matrices. A PCA was performed to show normalised interaction frequencies across all replicates for all pairs of 20kb bins.

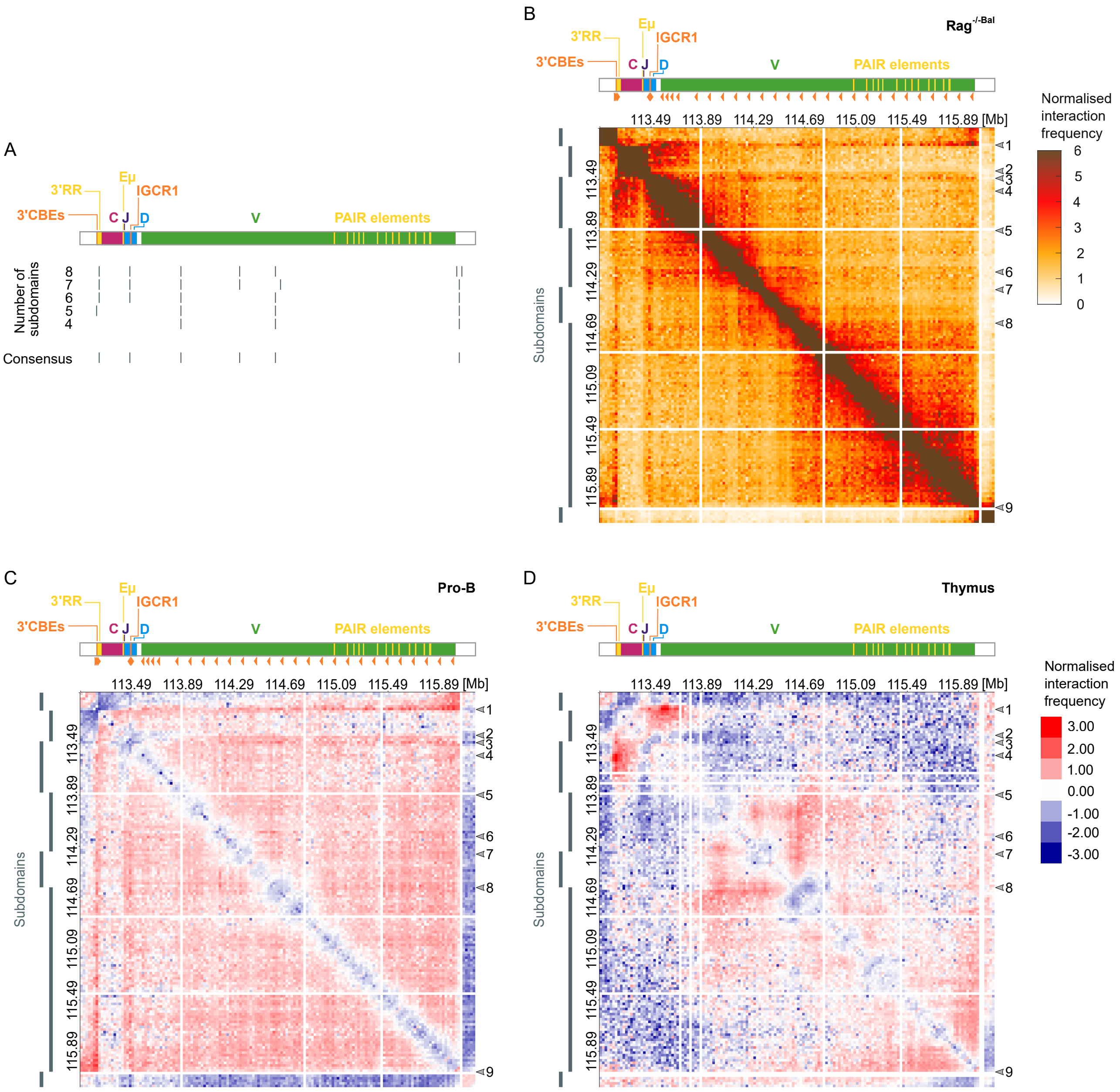

A
3'RR
Eμ
IGCR1
3'CBEs
C
J
D
V
PAIR elements
Number of subdomains
8
7
6
5
4
Consensus
B
Rag-/-Bal
113.49
113.89
114.29
114.69
115.09
115.49
115.89
[Mb]
Subdomains
Normalised interaction frequency
6
5
4
3
2
1
0
C
Pro-B
D
Thymus
3.00
2.00
1.00
0.00
-1.00
-2.00
-3.00

Figure S2. Interaction matrices of the Igh locus corrected for read coverage and distance. Related to Figure 2.

(A) Positions of subdomain boundaries determined by HiCseg when different numbers of subdomains are specified. Boundaries identified at least twice were taken as the consensus to define the subdomains.

(B) Read coverage corrected interaction matrix at 20kb resolution for the Igh baited region for $Rag^{-/-Bal}$ pro-B datasets.

(C) Read coverage and distance corrected interaction matrix at 20kb resolution for the Igh baited region for $Rag^{-/-Mom}$ pro-B and (D) thymus datasets.

All matrices were generated in Homer and biological replicates were averaged. White lines are bins with low read coverage excluded from analysis by Homer. Arrow 1 – 3'CBEs; arrow 2 – Eμ; arrow 3 – IGCR1; arrow 4 – most proximal V_H genes; arrow 5 – 5' of 7183 V_H gene family; arrow 6 – 5' of S107 V_H gene family; arrow 7 – 3' J606 V_H genes; arrow 8 – start of distal subdomain; arrow 9 - end of distal subdomain. The positions of subdomains in pro-B datasets determined using HiCseg are indicated by grey rectangles.

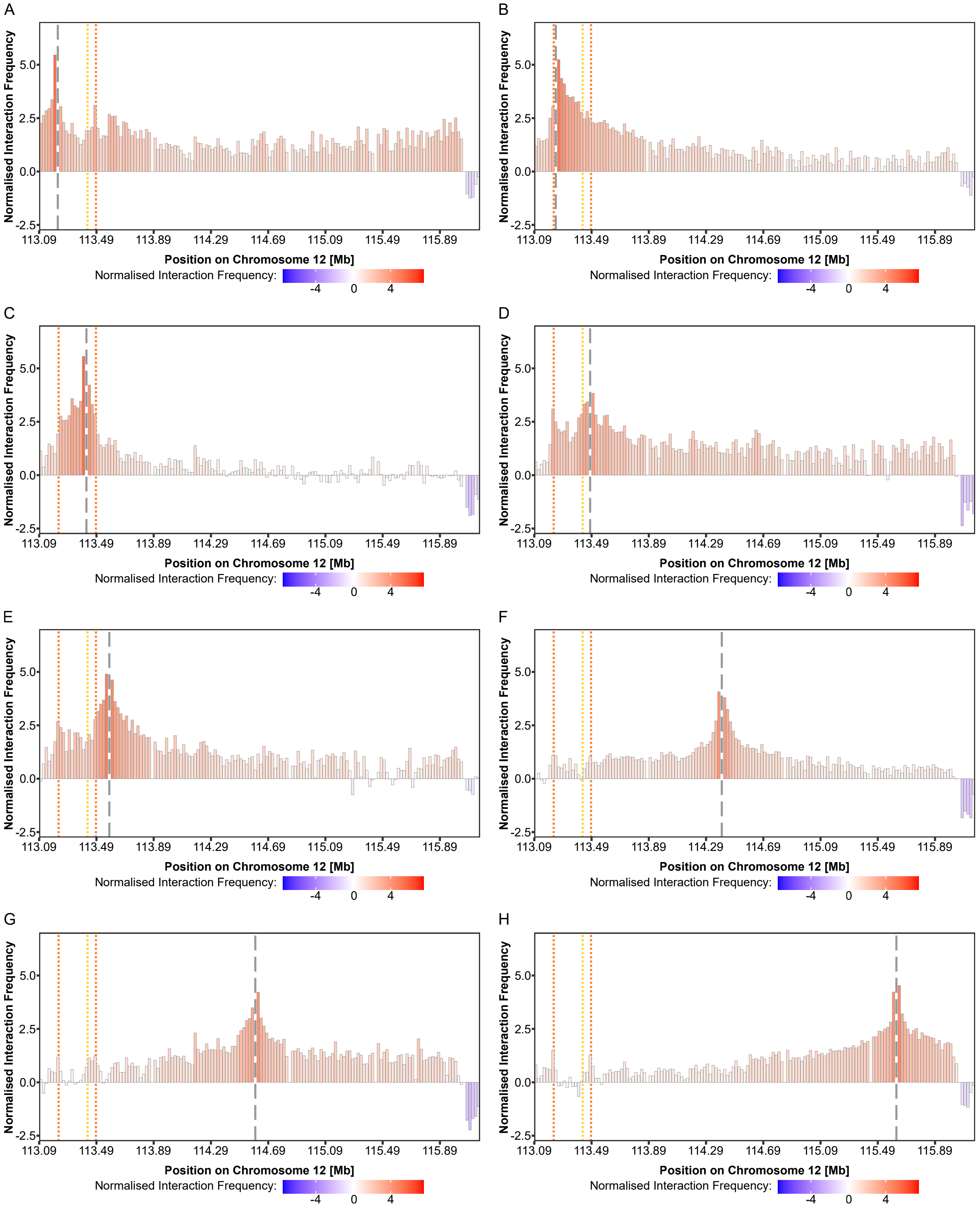
A
Normalised Interaction Frequency
5.0
2.5
0.0
-2.5
113.09
113.49
113.89
114.29
114.69
115.09
115.49
115.89
Position on Chromosome 12 [Mb]
Normalised Interaction Frequency:
-4
0
4
B
Normalised Interaction Frequency
5.0
2.5
0.0
-2.5
113.09
113.49
113.89
114.29
114.69
115.09
115.49
115.89
Position on Chromosome 12 [Mb]
Normalised Interaction Frequency:
-4
0
4
C
Normalised Interaction Frequency
5.0
2.5
0.0
-2.5
113.09
113.49
113.89
114.29
114.69
115.09
115.49
115.89
Position on Chromosome 12 [Mb]
Normalised Interaction Frequency:
-4
0
4
D
Normalised Interaction Frequency
5.0
2.5
0.0
-2.5
113.09
113.49
113.89
114.29
114.69
115.09
115.49
115.89
Position on Chromosome 12 [Mb]
Normalised Interaction Frequency:
-4
0
4
E
Normalised Interaction Frequency
5.0
2.5
0.0
-2.5
113.09
113.49
113.89
114.29
114.69
115.09
115.49
115.89
Position on Chromosome 12 [Mb]
Normalised Interaction Frequency:
-4
0
4
F
Normalised Interaction Frequency
5.0
2.5
0.0
-2.5
113.09
113.49
113.89
114.29
114.69
115.09
115.49
115.89
Position on Chromosome 12 [Mb]
Normalised Interaction Frequency:
-4
0
4
G
Normalised Interaction Frequency
5.0
2.5
0.0
-2.5
113.09
113.49
113.89
114.29
114.69
115.09
115.49
115.89
Position on Chromosome 12 [Mb]
Normalised Interaction Frequency:
-4
0
4
H
Normalised Interaction Frequency
5.0
2.5
0.0
-2.5
113.09
113.49
113.89
114.29
114.69
115.09
115.49
115.89
Position on Chromosome 12 [Mb]
Normalised Interaction Frequency:
-4
0
4

Figure S3. Virtual 4C interaction profiles of selected viewpoints in the Igh locus. Related to Figure 2.

Interaction counts were taken from the pro-B read coverage corrected interaction matrix in Figure 2A. The 20kb bin containing the viewpoint has been omitted for clarity and marked with a grey dashed line. Dotted lines indicate the positions of the 3'CBEs (left, orange), Eμ (middle, yellow) and IGCR1 (right, orange). (A) Interaction profile from 113.21-113.23Mb (bin containing 3'CBEs). (B) Interaction profile from 113.23-113.25Mb (bin containing 3'RR). (C) Interaction profile from 113.41-113.43Mb (bin containing Eμ). (D) Interaction profile from 113.47-113.49Mb (bin containing IGCR1). (E) Interaction profile from 113.57-113.59Mb (bin containing most 3' V_H gene). (F) Interaction profile from 114.39-114.41Mb (bin at 3' of J606 V_H gene family). (G) Interaction profile from 114.59-114.61Mb (bin preceding the distal domain). (H) Interaction profile from 115.61-115.63Mb (bin containing J558.71pg.172 and J558.72.173).

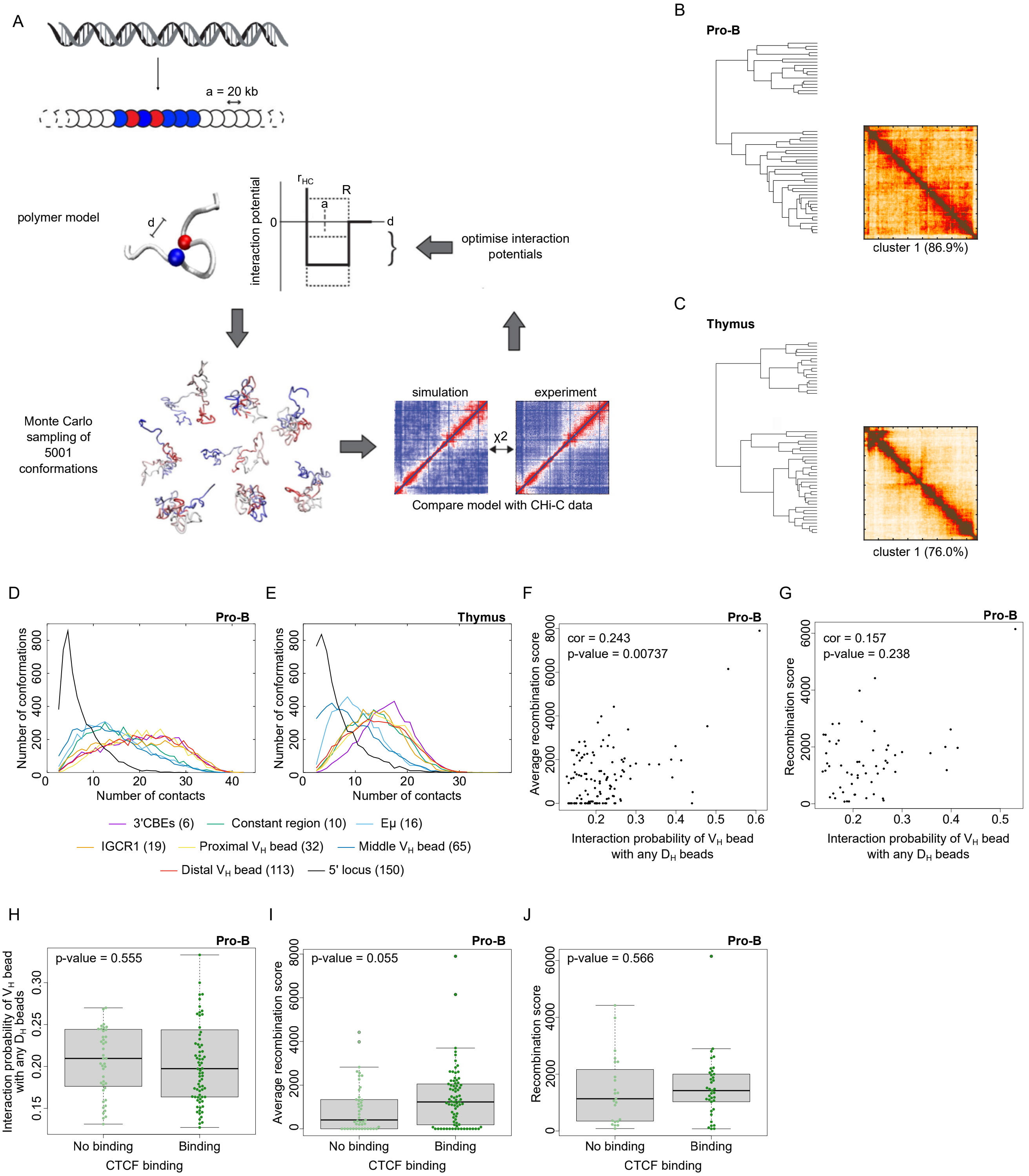

A
a = 20 kb
polymer model
d
interaction potential
r_HC
R
a
0
d
optimise interaction potentials
Monte Carlo sampling of 5001 conformations
simulation
experiment
χ2
Compare model with CHi-C data
B
Pro-B
cluster 1 (86.9%)
C
Thymus
cluster 1 (76.0%)
D
Pro-B
Number of conformations
Number of contacts
E
Thymus
Number of conformations
Number of contacts
3'CBEs (6)
Constant region (10)
Eμ (16)
IGCR1 (19)
Proximal V_H bead (32)
Middle V_H bead (65)
Distal V_H bead (113)
5' locus (150)
F
Pro-B
cor = 0.243
p-value = 0.00737
Average recombination score
Interaction probability of V_H bead with any D_H beads
G
Pro-B
cor = 0.157
p-value = 0.238
Recombination score
Interaction probability of V_H bead with any D_H beads
H
Pro-B
p-value = 0.555
Interaction probability of V_H bead with any D_H beads
No binding
Binding
CTCF binding
I
Pro-B
p-value = 0.055
Average recombination score
No binding
Binding
CTCF binding
J
Pro-B
p-value = 0.566
Recombination score
No binding
Binding
CTCF binding

Figure S4. Polymer modelling of Capture Hi-C. Related to Figure 3

(A) The chromatin fibre is treated as a string of beads, where adjacent beads correspond to consecutive 20kb of DNA sequence. There is no separation between beads (top). Schematic of the polymer model for structural deconvolution of CHi-C interaction frequencies (bottom). The model is constrained by the fixed distance between adjacent bead centres (*a=20kb*). Pairs of beads interact via short-range interaction potential (thick line) and each model gives a measure of pairwise distances between beads (*d*). $R=1.5a$ is a radius of a physical interaction, whereas $r_{HC}=0.6a$ is a hard-core repulsion radius. Monte Carlo sampling simulates the equilibrium ensemble of conformations and contact probabilities are compared to experimental CHi-C interaction frequency map using chi^2 (χ^2) as a dissimilarity score. Interaction potentials are iteratively optimised by further sampling (dotted lines) until simulated equilibrium ensemble converges with the experimental data.

(B and C) Single structures generated by the polymer model for pro-B (B) and thymus (C) datasets were clustered using differences in bead-to-bead contacts between each pair of conformations as a dissimilarity score.

(D and E) The distribution of the number of interacting partners for the 5001 conformations is shown for selected beads of the polymer model in pro-B cells (D) and thymus (E).

(F and G) There is weak correlation between V_H-D_H bead interaction probability and recombination score (provided by (Bolland et al., 2016)

(F) For all V_H beads, the correlation between the V_H-D_H bead interaction probability and the average recombination score of all V genes in that bead was determined. (G) For V_H beads containing a single active V_H gene, the correlation between the V_H-D_H interaction probability and the recombination score of the V_H gene was determined. cor = Spearman's correlation coefficient.

(H) CTCF binding does not impact V_H-D_H bead interactions. Boxplot shows the interaction probability of a given V_H bead with any D_H bead, where V_H beads are grouped depending on whether they bind CTCF or not. A Mann Whitney U test was performed.

(I and J) CTCF binding does not impact V_H-DJ_H recombination frequency. (I) The average recombination score of all V_H genes in a bead was compared between beads that do and do not bind CTCF. (J) For V_H beads containing a single active V_H gene, recombination score of that gene was compared between genes in beads that do and do not bind CTCF. A Mann Whitney U test was used in (I) and (J).

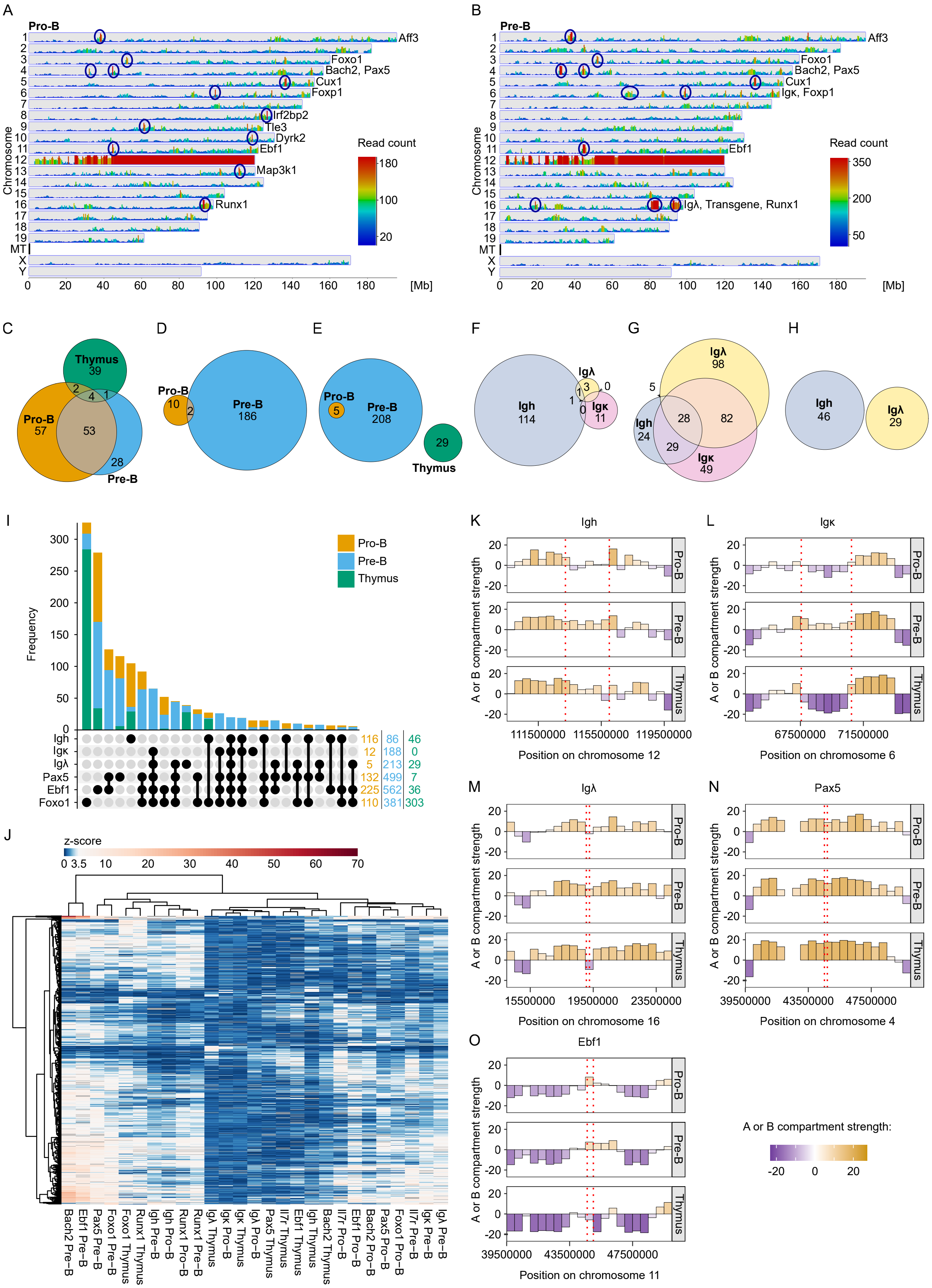
A
Pro-B
Aff3
Foxo1
Bach2, Pax5
Cux1
Foxp1
Irf2bp2
Tle3
Dyrk2
Ebf1
Map3k1
Runx1
Chromosome
Read count
180
100
20
[Mb]
B
Pre-B
Aff3
Foxo1
Bach2, Pax5
Cux1
Igκ, Foxp1
Ebf1
Igλ, Transgene, Runx1
Read count
350
200
50
C
Thymus 39
Pro-B 57
53
28
Pre-B
D
Pro-B 10
Pre-B 186
E
Pro-B 5
Pre-B 208
29
Thymus
F
Igh 114
Igλ
Igκ 11
G
Igλ 98
Igh 24
28
82
29
Igκ 49
H
Igh 46
Igλ 29
I
Frequency
Pro-B
Pre-B
Thymus
Igh
Igκ
Igλ
Pax5
Ebf1
Foxo1
J
z-score
0 3.5 10 20 30 40 50 60 70
Bach2 Pre-B
Ebf1 Pre-B
Pax5 Pre-B
Foxo1 Pre-B
Foxo1 Thymus
Runx1 Thymus
Igh Pre-B
Igh Pro-B
Runx1 Pro-B
Runx1 Pre-B
Igλ Thymus
Igκ Pro-B
Igκ Thymus
Igλ Pro-B
Pax5 Thymus
Il7r Thymus
Ebf1 Thymus
Igh Thymus
Bach2 Thymus
Il7r Pro-B
Ebf1 Pro-B
Bach2 Pro-B
Pax5 Pro-B
Foxo1 Pro-B
Il7r Pre-B
Igκ Pre-B
Igλ Pre-B
K
Igh
A or B compartment strength
Position on chromosome 12
L
Igκ
Position on chromosome 6
M
Igλ
Position on chromosome 16
N
Pax5
Position on chromosome 4
O
Ebf1
Position on chromosome 11
A or B compartment strength:
-20 0 20

Figure S5. Inter-chromosomal contacts of the Ig loci. Related to Figure 5.
(A and B) V4Cs from the Igh baited region were performed on Scribler-processed and blacklisted CHi-C datasets. Other ends were quantified in 0.5Mb bins genome wide and biological replicates were averaged. Genome wide views of interactions from the Igh are shown in (A) pro-B cells and (B) pre-B cells.
(C-E) Venn diagrams show shared significant (z-score>3.5) interchromosomal interactions from the (C) Igh viewpoint; (D) Igκ viewpoint; (E) Igλ viewpoint.
(F-H) The interchromosomal interactions are developmental stage specific and the Ig loci share interaction partners in pre-B cells. Venn diagrams show the shared significant (z-score>3.5) interactions in (F) Pro-B; (G) Pre-B; (G) Thymus.
(I) UpSet plot showing the number of unique and shared interchromosomal interactions between the three Ig loci, Pax5, Ebf1, and Foxo1 at each developmental stage. Numbers to the right indicate the total number of interchromosomal interactions for each viewpoint; only the top 25 intersection patterns (based on the sum across developmental stages) are shown. Note that whilst the bars are stacked to facilitate visualisation, interactions at a given developmental stage will overlap with those at other stages, as shown for the Ig loci in C-D.
(J) Hierarchical clustering shows similarities between interchromosomal interactions from the Ig loci and baited regions implicated in B cell function. 0.5Mb bins were selected for hierarchical clustering if they had a significant interaction with any of the viewpoints of interest (Igh, Igκ, Igλ, Bach2, Ebf1, Foxo1, Notch1, Pax5, Runx1 and Il7r) at any developmental stage. Clustering, based on average z-score values, was performed using the pheatmap package in R. The distance measure "Euclidean" and the clustering method "complete" were used.
(K-O) A or B compartment strength is shown in 0.5Mb bins for 10.5Mb regions centred on loci of interest: (K) Igh, (L) Igκ, (M) Igλ, (N) Pax5 and (O) Ebf1. Values >0 indicate A compartments and values <0 indicate B compartments. The position of the locus of interest is indicated by dotted red lines. Empty bins, seen in (M), arise due to low read coverage precluding calculationof PC1 values in Homer.

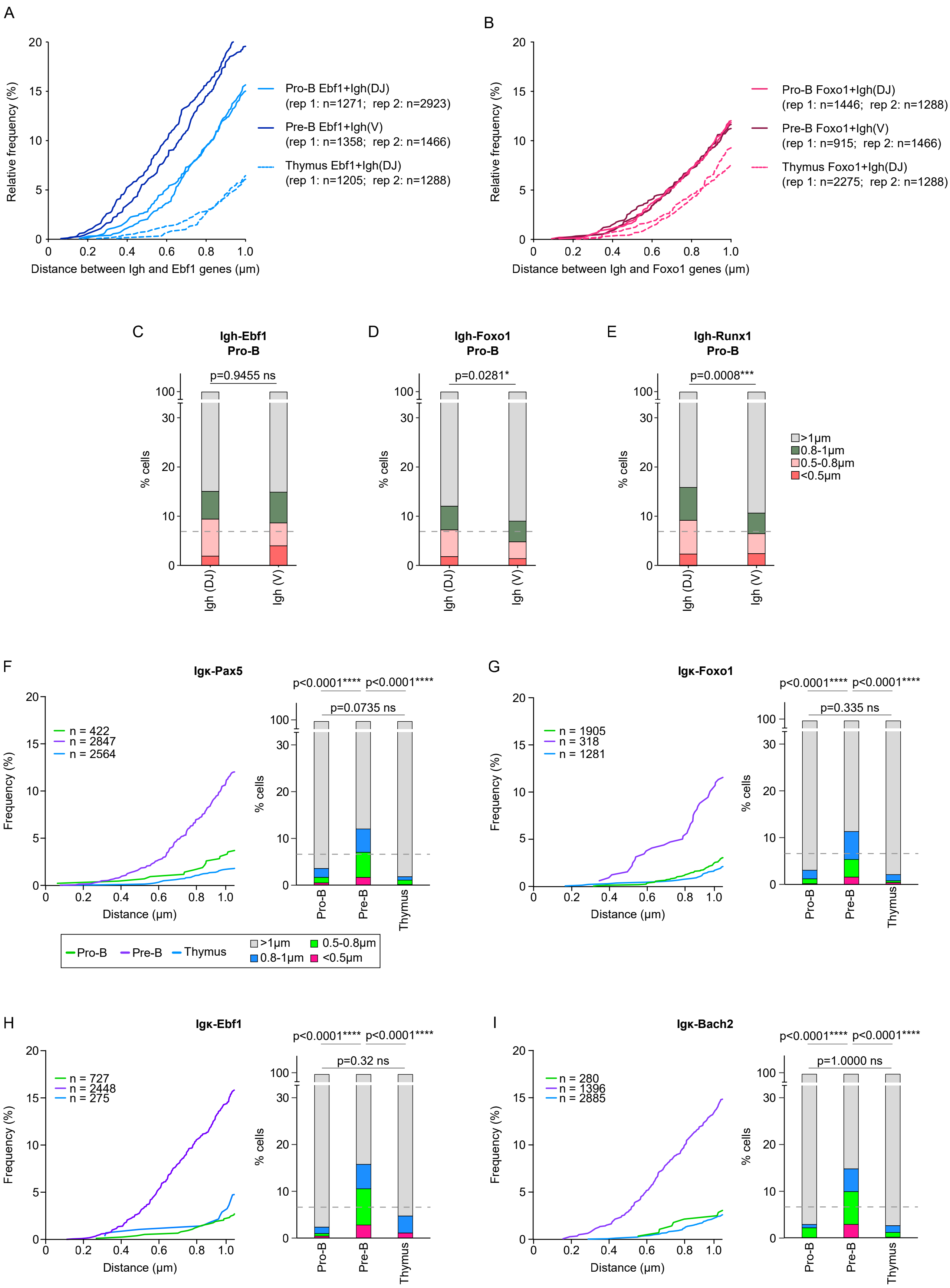

A
Relative frequency (%)
Distance between Igh and Ebf1 genes (μm)
Pro-B Ebf1+Igh(DJ)
(rep 1: n=1271; rep 2: n=2923)
Pre-B Ebf1+Igh(V)
(rep 1: n=1358; rep 2: n=1466)
Thymus Ebf1+Igh(DJ)
(rep 1: n=1205; rep 2: n=1288)
B
Relative frequency (%)
Distance between Igh and Foxo1 genes (μm)
Pro-B Foxo1+Igh(DJ)
(rep 1: n=1446; rep 2: n=1288)
Pre-B Foxo1+Igh(V)
(rep 1: n=915; rep 2: n=1466)
Thymus Foxo1+Igh(DJ)
(rep 1: n=2275; rep 2: n=1288)
C
Igh-Ebf1
Pro-B
p=0.9455 ns
% cells
Igh (DJ)
Igh (V)
D
Igh-Foxo1
Pro-B
p=0.0281*
% cells
Igh (DJ)
Igh (V)
E
Igh-Runx1
Pro-B
p=0.0008***
% cells
Igh (DJ)
Igh (V)
>1μm
0.8-1μm
0.5-0.8μm
<0.5μm
F
Igκ-Pax5
n = 422
n = 2847
n = 2564
Frequency (%)
Distance (μm)
p<0.0001**** p<0.0001****
p=0.0735 ns
% cells
Pro-B
Pre-B
Thymus
Pro-B Pre-B Thymus
>1μm 0.5-0.8μm
0.8-1μm <0.5μm
G
Igκ-Foxo1
n = 1905
n = 318
n = 1281
Frequency (%)
Distance (μm)
p<0.0001**** p<0.0001****
p=0.335 ns
% cells
Pro-B
Pre-B
Thymus
H
Igκ-Ebf1
n = 727
n = 2448
n = 275
Frequency (%)
Distance (μm)
p<0.0001**** p<0.0001****
p=0.32 ns
% cells
Pro-B
Pre-B
Thymus
I
Igκ-Bach2
n = 280
n = 1396
n = 2885
Frequency (%)
Distance (μm)
p<0.0001**** p<0.0001****
p=1.0000 ns
% cells
Pro-B
Pre-B
Thymus

Figure S6. 3D FISH data is reproducible and validates Chi-C interchromosomal interactions of Igh and Igκ. Related to Figure 5.

Biological replicates of 3D FISH experiments for (A) Igh + Ebf1 and (B) Igh + Foxo1 were highly reproducible. The Igh (DJ) probe was used for pro-B and thymus, and the Igh (V) probe for pre-B. The closest signals in each cell were used. n = number of cells analysed per slide.

(C-E) The D-J region participates in interchomosomal interactions more frequently than the distal V region. Distances between the Igh and the locus of interest in pro-B cells (Ebf1 in (C); Foxo1 in (D); Runx1 in (E)) were compared when using the V FISH probe (BAC RP23-70F21) and the DJ FISH probe (BAC RP23-109B20) depicted in Figure 6. A Mann-Whitney test was performed. The distances <1μm are plotted in three categories: <0.5μm, 0.5-0.8μm and 0.8-1μm; Dashed line denotes the probability of a random interaction at a distance of <1μm of 6.9%.

(F-I) 3D FISH probing of the Igκ locus validates Igk interchromosomal interactions. Igκ with (F) Pax5, (G) Foxo1, (H) Ebf1 or (I) Bach2. Igκ probes RP24-179E20 and RP23-124O23 were labelled with Alexa Fluor 488 (green). Line graphs show cumulative distributions of distances <1μm between the Igκ locus and genes of interest in three cell types. The closest signal pairs in each cell were used. n=number of nuclei analysed. Bar graphs show distribution of distances grouped into four brackets (<0.5μm, 0.5-0.8μm, 0.8-1μm, >1μm). Dashed line denotes the probability of a random interaction at a distance of <1μm of 6.9%. P-values were calculated using the Fisher's exact test with Bonferroni correction.

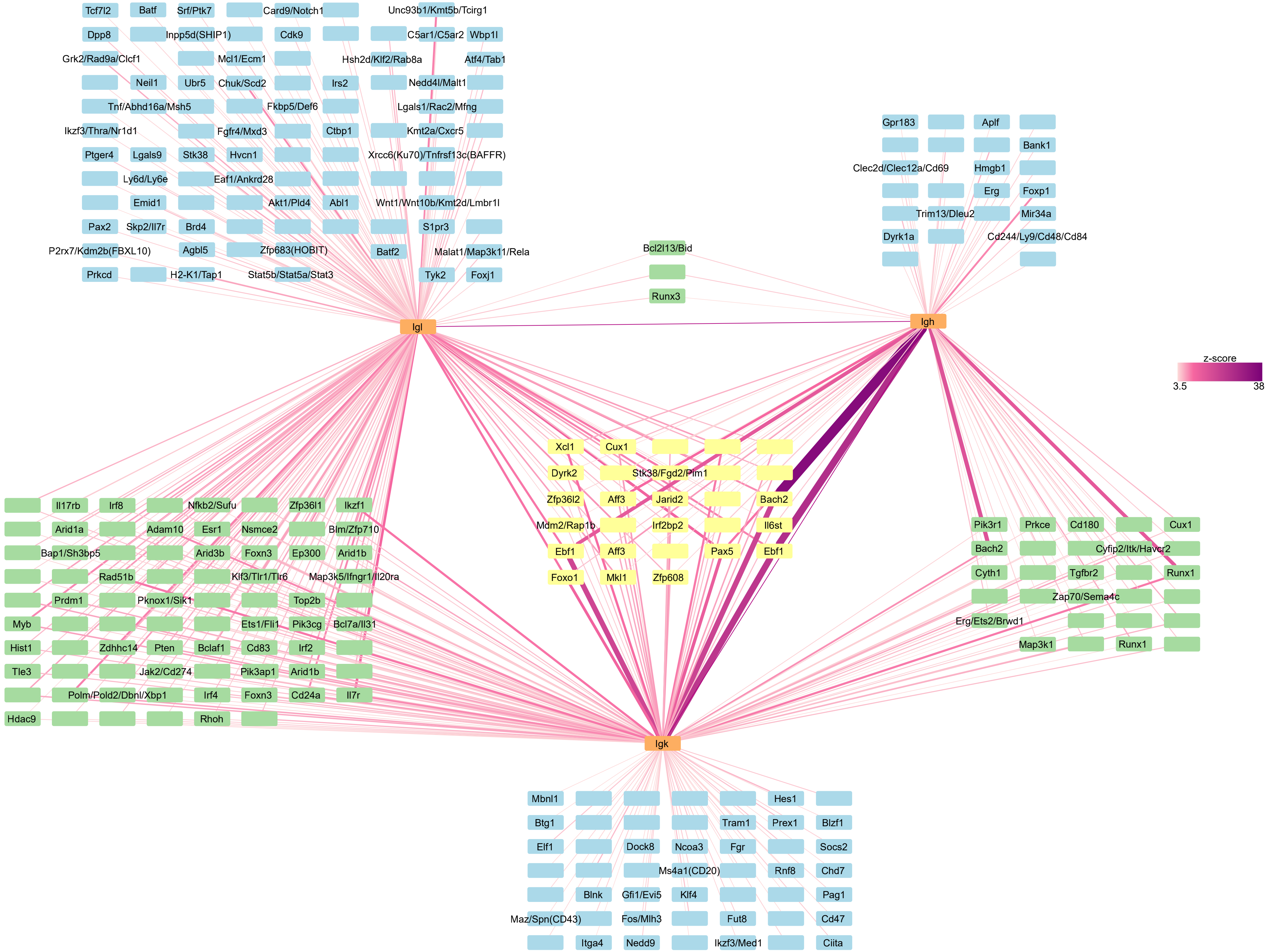
Tcf7l2
Batf
Srf/Ptk7
Card9/Notch1
Unc93b1/Kmt5b/Tcirg1
Dpp8
Inpp5d(SHIP1)
Cdk9
C5ar1/C5ar2
Wbp1l
Grk2/Rad9a/Clcf1
Mcl1/Ecm1
Hsh2d/Klf2/Rab8a
Atf4/Tab1
Neil1
Ubr5
Chuk/Scd2
Irs2
Nedd4l/Malt1
Tnf/Abhd16a/Msh5
Fkbp5/Def6
Lgals1/Rac2/Mfng
Ikzf3/Thra/Nr1d1
Fgfr4/Mxd3
Ctbp1
Kmt2a/Cxcr5
Ptger4
Lgals9
Stk38
Hvcn1
Xrcc6(Ku70)/Tnfrsf13c(BAFFR)
Ly6d/Ly6e
Eaf1/Ankrd28
Emid1
Akt1/Pld4
Abl1
Wnt1/Wnt10b/Kmt2d/Lmbr1l
Pax2
Skp2/Il7r
Brd4
S1pr3
P2rx7/Kdm2b(FBXL10)
Agbl5
Zfp683(HOBIT)
Batf2
Malat1/Map3k11/Rela
Prkcd
H2-K1/Tap1
Stat5b/Stat5a/Stat3
Tyk2
Foxj1
Gpr183
Aplf
Bank1
Clec2d/Clec12a/Cd69
Hmgb1
Erg
Foxp1
Trim13/Dleu2
Mir34a
Dyrk1a
Cd244/Ly9/Cd48/Cd84
Bcl2l13/Bid
Runx3
Igl
Igh
z-score
3.5
38
Xcl1
Cux1
Dyrk2
Stk38/Fgd2/Pim1
Zfp36l2
Aff3
Jarid2
Bach2
Mdm2/Rap1b
Irf2bp2
Il6st
Ebf1
Aff3
Pax5
Ebf1
Foxo1
Mkl1
Zfp608
Il17rb
Irf8
Nfkb2/Sufu
Zfp36l1
Ikzf1
Arid1a
Adam10
Esr1
Nsmce2
Blm/Zfp710
Bap1/Sh3bp5
Arid3b
Foxn3
Ep300
Arid1b
Rad51b
Klf3/Tlr1/Tlr6
Map3k5/Ifngr1/Il20ra
Prdm1
Pknox1/Sik1
Top2b
Myb
Ets1/Fli1
Pik3cg
Bcl7a/Il31
Hist1
Zdhhc14
Pten
Bclaf1
Cd83
Irf2
Tle3
Jak2/Cd274
Pik3ap1
Arid1b
Polm/Pold2/Dbnl/Xbp1
Irf4
Foxn3
Cd24a
Il7r
Hdac9
Rhoh
Pik3r1
Prkce
Cd180
Cux1
Bach2
Cyfip2/Itk/Havcr2
Cyth1
Tgfbr2
Runx1
Zap70/Sema4c
Erg/Ets2/Brwd1
Map3k1
Runx1
Igk
Mbnl1
Hes1
Btg1
Tram1
Prex1
Blzf1
Elf1
Dock8
Ncoa3
Fgr
Socs2
Ms4a1(CD20)
Rnf8
Chd7
Blnk
Gfi1/Evi5
Klf4
Pag1
Maz/Spn(CD43)
Fos/Mlh3
Fut8
Cd47
Itga4
Nedd9
Ikzf3/Med1
Ciita

Figure S7. Interchromosomal interaction network of the Ig loci in pre-B cells. Related to Figure 6.

The nodes correspond to 0.5Mb bins in which interchromosomal interactions were analysed. Bins contacted by all three Ig loci are indicated by yellow nodes, bins contacted by two Ig loci are indicated by green nodes, and bins contacted by only one Ig locus are indicated by blue nodes. The thickness and shade of the purple lines linking the nodes represent the frequency of the interaction (z-score value). Putative genes of interest are annotated for selected nodes.
