# Supplementary material for: Intra- and interchromosomal contact mapping reveals the Igh locus has extensive conformational heterogeneity and interacts with B-lineage genes
Source: Cell Rep. 2023 Sep 6;42(9):113074. doi: 10.1016/j.celrep.2023.113074 (PMC10548092; doi:10.1016/j.celrep.2023.113074)
Supplement: Document S2. Article plus supplemental information [file mmc8.pdf]

Cell Reports

Article

Intra- and interchromosomal contact mapping reveals the *Igh* locus has extensive conformational heterogeneity and interacts with B-lineage genes

Graphical abstract

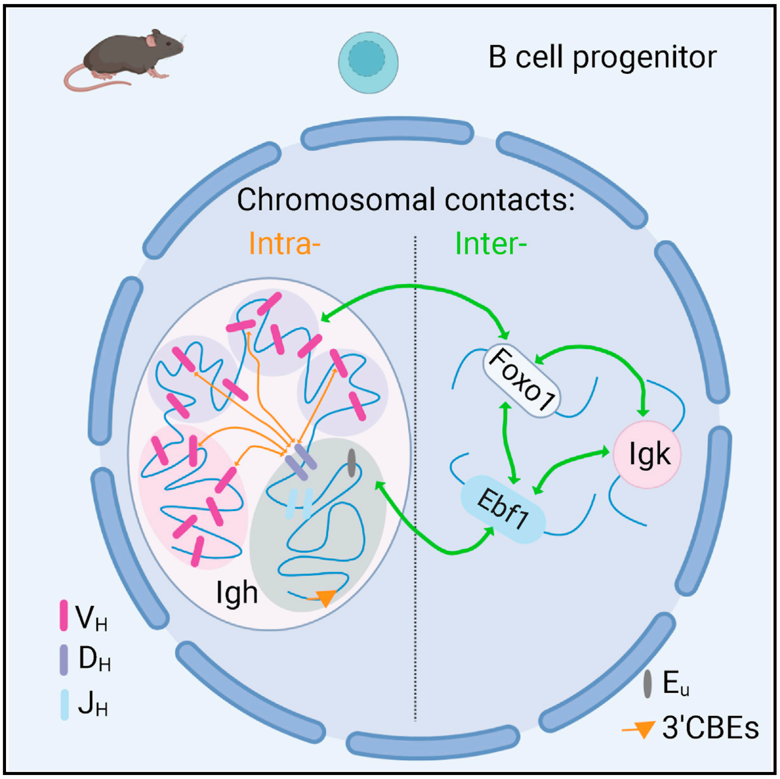

Authors

Olga Mielczarek, Carolyn H. Rogers, Yinxiu Zhan, ..., Peter Fraser, Luca Giorgetti, Anne E. Corcoran

Correspondence

anne.corcoran@babraham.ac.uk

In brief

Mielczarek et al. use Capture Hi-C to obtain a detailed map of chromosomal interactions within the immunoglobulin heavy-chain locus. Polymer modeling shows that each Igh structure is unique, enabling antibody diversity. The Ig loci associate with key B lineage genes in a developmental-stage-specific interchromosomal network.

Highlights

- Capture Hi-C maps intra- and interchromosomal contacts of the immunoglobulin heavy chain
- Polymer modeling shows thousands of different Igh structures that underpin diversity
- The Ig loci participate in a stage-specific interchromosomal network with B lineage genes

Mielczarek et al., 2023, Cell Reports *42*, 113074
September 26, 2023 © 2023 The Authors.
https://doi.org/10.1016/j.celrep.2023.113074

CellPress

Cell Reports

CellPress
OPEN ACCESS

Article

Intra- and interchromosomal contact mapping reveals the *Igh* locus has extensive conformational heterogeneity and interacts with B-lineage genes

Olga Mielczarek,[1,6,16] Carolyn H. Rogers,[1,2,16] Yinxiu Zhan,[3,7,16] Louise S. Matheson,[1,2] Michael J.T. Stubbington,[1,8] Stefan Schoenfelder,[1] Daniel J. Bolland,[1,2,9] Biola M. Javierre,[1,10] Steven W. Wingett,[4,11] Csilla Várnai,[1,12] Anne Segonds-Pichon,[4,13] Simon J. Conn,[5] Felix Krueger,[4,14] Simon Andrews,[4] Peter Fraser,[1,15] Luca Giorgetti,[3] and Anne E. Corcoran[1,2,17,*]

[1]Nuclear Dynamics Programme, Babraham Institute, Babraham Research Campus, Cambridge CB22 3AT, UK
[2]Immunology Programme, Babraham Institute, Babraham Research Campus, Cambridge CB22 3AT, UK
[3]Friedrich Miescher Institute for Biomedical Research, Maulbeerstrasse 66, 4058 Basel, Switzerland
[4]Bioinformatics Group, Babraham Institute, Babraham Research Campus, Cambridge CB22 3AT, UK
[5]Flinders Health and Medical Research Institute, College of Medicine and Public Health, Flinders University, Bedford Park, SA 5042, Australia
[6]Present address: Horizon Discovery (PerkinElmer) Cambridge Research Park, 8100 Beach Dr., Waterbeach, Cambridge CB25 9TL, UK
[7]Present address: Instituto Europeo di Oncologia, Via Adamello 16, 20139 Milan, Italy
[8]Present address: CS Genetics, Cambridge, UK
[9]Present address: PetMedix, B940, Babraham Research Campus, Cambridge CB22 3FH, UK
[10]Present address: Josep Carreras Leukemia Research Institute, Campus ICO-Germans Trias i Pujol Ctra de Can Ruti, 08916 Barcelona, Spain
[11]Present address: MRC Laboratory of Molecular Biology, Cambridge Biomedical Campus, Francis Crick Ave, Trumpington, Cambridge CB2 0QH, UK
[12]Present address: Institute of Cancer and Genomic Science, University of Birmingham, 6 Mindelsohn Way, Birmingham B15 2SY, UK
[13]Present address: MRC Unit The Gambia at LSHTM, Atlantic Boulevard, Fajara, The Gambia
[14]Present address: Altos Labs, Cambridge, UK
[15]Present address: Department of Biological Science, Florida State University, 319 Stadium Drive, Tallahassee, FL 32306-4295, USA
[16]These authors contributed equally
[17]Lead contact
*Correspondence: anne.corcoran@babraham.ac.uk
https://doi.org/10.1016/j.celrep.2023.113074

SUMMARY

To produce a diverse antibody repertoire, immunoglobulin heavy-chain (*Igh*) loci undergo large-scale alterations in structure to facilitate juxtaposition and recombination of spatially separated variable (V_H), diversity (D_H), and joining (J_H) genes. These chromosomal alterations are poorly understood. Uncovering their patterns shows how chromosome dynamics underpins antibody diversity. Using tiled Capture Hi-C, we produce a comprehensive map of chromatin interactions throughout the 2.8-Mb *Igh* locus in progenitor B cells. We find that the *Igh* locus folds into semi-rigid subdomains and undergoes flexible looping of the V_H genes to its 3′ end, reconciling two views of locus organization. Deconvolution of single *Igh* locus conformations using polymer simulations identifies thousands of different structures. This heterogeneity may underpin the diversity of V(D)J recombination events. All three immunoglobulin loci also participate in a highly specific, developmentally regulated network of interchromosomal interactions with genes encoding B cell-lineage factors. This suggests a model of interchromosomal coordination of B cell development.

INTRODUCTION

During B cell development in the bone marrow, the immunoglobulin heavy-chain (*Igh*) and light-chain (*Igk* and *Igl*) loci undergo somatic recombination to generate a vast array of antigen-specific B cell receptors (BCRs). V(D)J recombination is catalyzed by the endonuclease complex RAG, encoded by the recombination activation genes *Rag1* and *Rag2*.[1] RAG expression initiates in common lymphoid progenitors (CLPs) to activate D-J recombination, completed on both *Igh* alleles by the early pro-B cell stage. One allele then undergoes variable (V_H)-DJ_H recombination in committed pro-B cells.[2] After productive recombination, the light chain encoded by the *Igk* locus recombines in pre-B cells. Surface expression of the heavy and light chain together forms the mature BCR in immature B cells.

The mouse *Igh* comprises 195 V_H (spanning 2.5 Mb), 10 diversity (D_H) (60 kb), four joining (J_H) (1.5 kb) genes, and eight constant (C) genes. The V_H genes belong to 16 families based on

 © 2023 The Authors.
This is an open access article under the CC BY license (http://creativecommons.org/licenses/by/4.0/).

sequence homology, grouped into proximal (320 kb nearer the 3′ end), middle (560 kb), and distal (1.6 Mb nearer the 5′ end) V_H genes.[3] 128 V_H genes actively recombine,[4] and participation of all is essential for antibody diversity. The locus also harbors several regulatory elements, including the intronic enhancer Eμ, which promotes transcription of the recombined heavy chain,[5] and the intergenic control region I (IGCR1), an insulator that ensures sequential recombination and equilibrates usage of proximal and distal V_H genes.[6–9] At the 3′ end, the 3′ regulatory region (3′ RR) modulates transcription, while the 3′ superanchor, composed of several CCCTC-binding factor (CTCF)-binding elements (3′ CBEs) provides insulation at the 3′ boundary (Figure S2A).[10–12]

Chromatin is non-randomly organized in the nucleus and its spatial conformation influences gene expression.[13–15] Chromosomes occupy discrete territories,[16,17] and the majority of genomic interactions are intrachromosomal.[18] However, genes can occasionally loop out of their chromosome territories to interact in *trans* with other genomic regions.[19,20] Chromatin segregates into euchromatic A compartments and heterochromatic B compartments,[18] which are divided into topologically associating domains (TADs) up to megabases in size,[21,22] reviewed in Galupa and Heard.[23] TADs tend to interact internally and are often flanked by convergent CTCF-binding sites.[24,25] The *Igh* locus forms its own 2.8-Mb TAD.[12,26,27]

The *Igh* locus undergoes developmentally controlled conformational and epigenetic changes to facilitate V(D)J recombination. Prior to the pro-B cell stage, the *Igh* is tethered to the nuclear lamina via its 5′ V_H region, which bears repressive chromatin marks,[28,29] while, concomitantly with D-J recombination, the 3′ region gains active marks.[30,31] At the onset of V_H-DJ_H recombination in pro-B cells, the *Igh* locus relocates to the center of the nucleus[32,33] and gains active marks over the V_H region.[34,35] To ensure that all V_H genes have an opportunity to recombine, they are brought into physical proximity of the D-J region by large-scale locus contraction and DNA looping.[33,36,37] Thus, the *Igh* elements become juxtaposed and confined to a much smaller 3D space than expected from their genomic distance.[38]

Fluorescence *in situ* hybridization (FISH) studies demonstrated that, in pro-B cells, the D_H-proximal and distal V_H genes are both close to the 3′ end of the *Igh*,[39] and chromosome conformation capture 4C-seq suggested that they interact flexibly therein.[40] The 3′ CBEs contact all V_H genes,[12,41] facilitated by "bouncing" trajectories of V_H genes in a constrained viscoelastic environment.[38] Multi-probe FISH suggested hierarchical folding of the *Igh* locus is dependent on key B cell transcription factors,[42] and a 5C study described three major subdomains linked by extremely long-range interactions.[43] To date, there is consensus that the 3′ end forms a subdomain defined by interactions between the 3′ CBEs, 3′ RR, Eμ, and IGCR1 and includes the proximal V_H genes, which recombine without locus contraction.[36,44–46] However, locus contraction and long-range interactions between middle and distal V_H genes and the D-J region are thought to be mediated either by a continuum of dynamic loops[12,38,40] or by the formation of overarching stable subdomains.[42,43] These contrasting models emerged from alternative methods above, and translating them into a comprehensive model that elucidates the looping of the middle and distal V_H genes that ensures balanced V_H gene usage has been a major challenge. Furthermore, recent discoveries of DNA loops extruded by cohesin between multiple V_H region CTCF sites convergent with CTCF sites in the IGCR1 and the 3′ CBEs,[47–49] together with RAG endonuclease scanning facilitated by convergent complementary RSSs,[50,51] shows that loop extrusion plays a widespread role but raises further questions about the size, frequency, and complexity of DNA loops within the *Igh* locus. Resolution of these questions to provide a clear picture of *Igh* locus structure requires an unbiased high-resolution map of all-to-all interactions of the *Igh* elements in pro-B cells poised for recombination.

Here, we develop a tiled Capture Hi-C (CHi-C) for the immunoglobulin (Ig) loci and other B lineage-defining loci and produce the first enriched all-to-all map of interactions between *Igh* elements, providing the most comprehensive and highest resolution picture to date. We show that the main mediators of interactions between the 3′ end of the locus and the V_H genes are the 3′ CBEs superanchor and the IGCR1 insulator. Contrary to previous models of a DJ-centric *Igh* structure, the distal V_H genes form a large subdomain with which the 3′ CBEs and the IGCR1 interact. Polymer modeling yields thousands of individual structures, revealing that *Igh* conformations are a highly heterogeneous ensemble, which we propose underpins the diversity of the antibody repertoire. Furthermore, CHi-C revealed developmental-stage-specific genome-wide contacts *in trans* between the *Ig* loci and key genes driving B cell development, indicating that a network of interchromosomal contacts between key B cell genes may contribute to their coordinated regulation.

RESULTS

Generation of a high-resolution all-to-all interaction map of the *Igh* locus

The *Igh* locus has over 50% repetitive sequence and 195 similar V_H genes,[3] together leading to lower read coverage than the rest of the genome in next-generation sequencing (NGS), making it a challenging target for conventional Hi-C approaches (Figure S1A). To produce a comprehensive all-to-all, high-resolution, and unbiased map of chromatin interactions within the *Igh* locus, we applied CHi-C to determine genomic contacts involving the *Igh* from Hi-C libraries prepared from *ex vivo* pro-B cells from $Rag1^{-/-}$ mice. These contacts were enriched using short biotinylated RNA baits transcribed from every HindIII restriction fragment end within bacterial artificial chromosomes (BACs) covering the Ig loci and other selected regions (Figures 1A, 1B, S1B, S1G, and S1H). CHi-C achieved approximately 30-fold read coverage enrichment over Hi-C libraries (Figures 1C and S1C; Table S1), which was instrumental in capturing intralocus interactions to unprecedented depth. Importantly, the baits generated from 21 consecutive BACs covering the 2.8-Mb *Igh* locus (Figure S1D) enriched the Hi-C material with uniform efficiency (Figures S1E and S1F).

To validate the assay, we show a known interaction between a baited Hist1 locus and a non-baited Hist1 locus 1 Mb upstream, with no interaction detected with the intervening baited Vmrn

CellPress
OPEN ACCESS

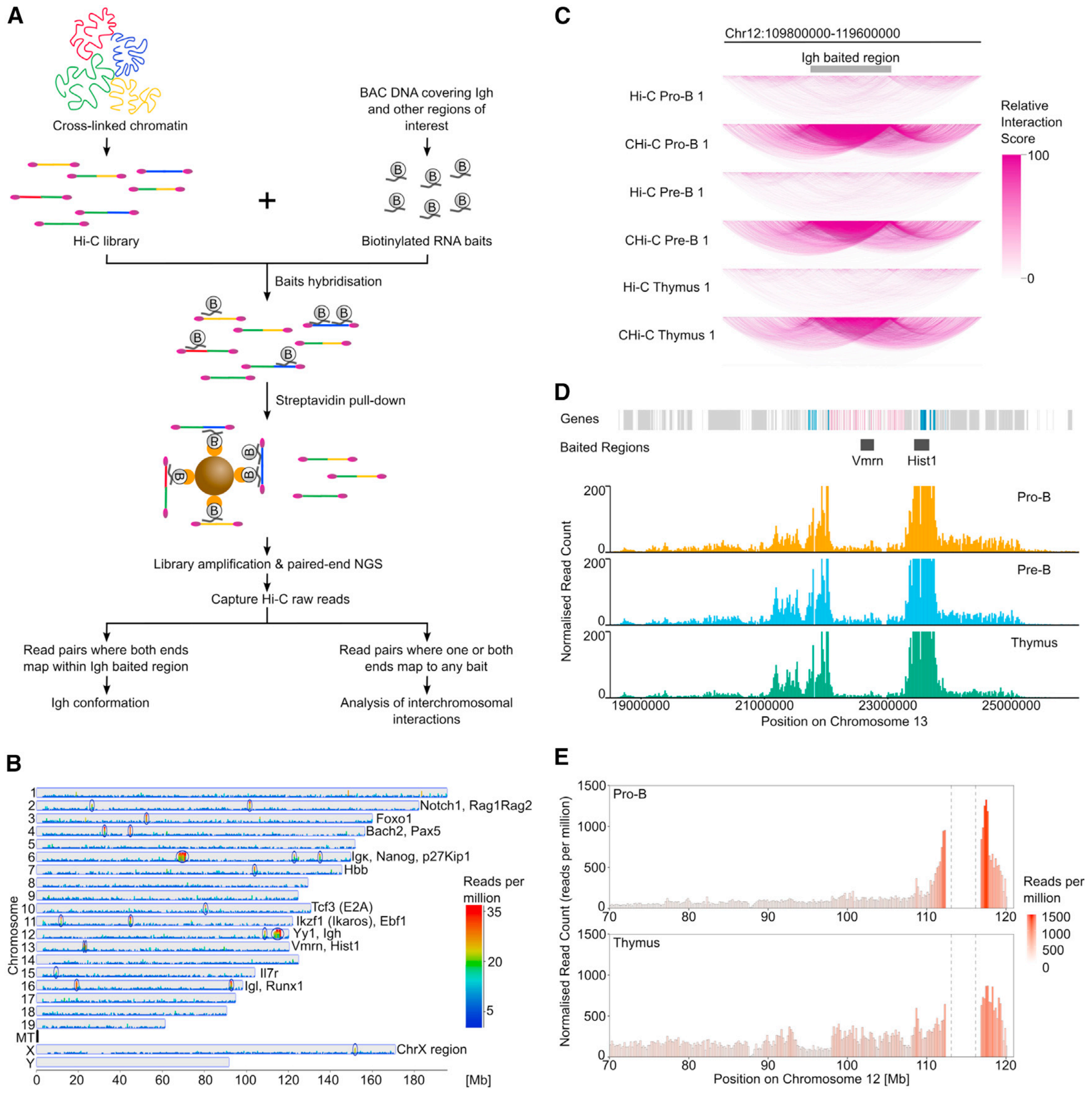

Figure 1. Capture Hi-C method

(A) Capture Hi-C (CHi-C) workflow. Top left: cross-linked chromatin was used to generate Hi-C libraries. Top right: BAC DNA was digested, sonicated, and *in vitro* transcribed to yield a library of biotinylated RNA baits. The Hi-C and RNA bait libraries were hybridized together and baited fragments captured with streptavidin beads. Captured sequences were amplified and paired-end sequenced. The resulting CHi-C libraries yielded intralocus *Igh* reads (bottom left) and interchromosomal reads (bottom right).

(B) Genome-wide view of read coverage in HiCUP-processed CHi-C Rag1$^{Mom-/-}$ pro-B 1 dataset. Reads were quantified in 100-bp bins and normalized per million reads. Read coverage is enriched at baited regions (blue circles).

(C) Read enrichment over the *Igh* locus in CHi-C datasets. Five million randomly sampled reads from HiCUP-processed Hi-C and CHi-C datasets were quantified in 200-kb bins and visualized in the WashU Epigenome Browser. Arcs show interactions. Arc color indicates the number of interactions. One representative replicate is shown for each dataset type.

(D) Previously reported interactions between the *histone 1* (*Hist1*) clusters on chromosome 13 were detected in all CHi-C samples. A virtual 4C from the Hist1 baited region was performed on one and both ends in Scribler-processed datasets. *Hist1* genes are in blue. Vomeronasal (Vmrn) genes are in pink.

(E) The *Igh* locus interacts more frequently with regions outside the locus in *cis* in thymus than in pro-B cells. A virtual 4C was conducted from the baited *Igh* region in Scribler-processed datasets. Biological replicates were averaged.

See also Figure S1 and Table S1.

locus (Figure 1D). We also show that the *Igh* has more frequent intralocus interactions in pro-B cells, where it is active and contracted, compared to thymus, where it is less contracted (Figure 1E), in agreement with previous reports.[40]

Igh harbors two key anchors mediating multiple CTCF-driven long-range interactions

We produced intralocus interaction maps of the *Igh* in pro-B cells from two different Rag1$^{-/-}$ models (Figures 2A and S2B,

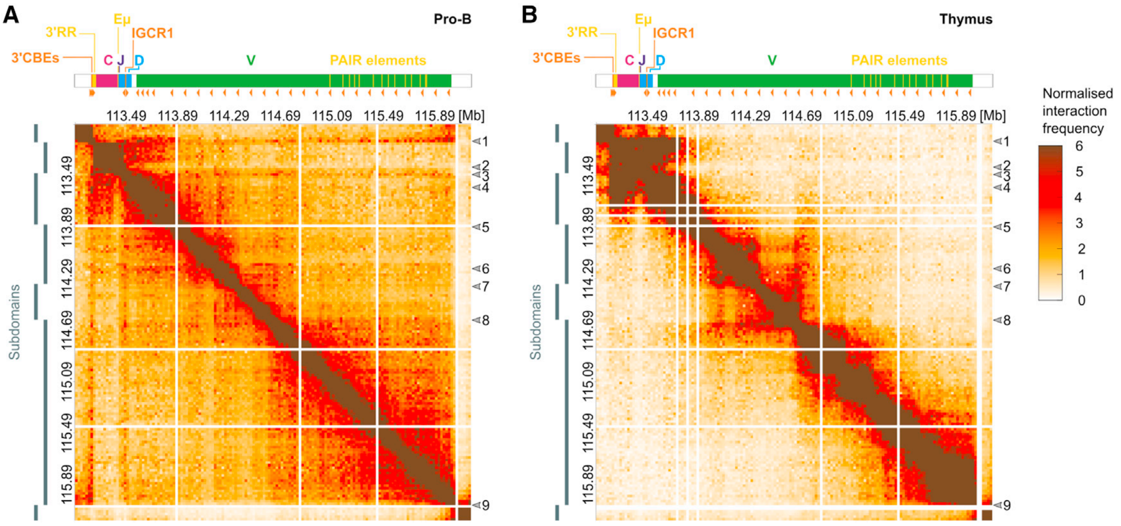

Figure 2. All-to-all interaction matrices of the *Igh locus*

Interaction matrices at 20-kb resolution for the *Igh* baited region for (A) pro-B and (B) thymus datasets. Each matrix entry is a coverage normalized interaction frequency value for the pairwise interaction between two 20-kb bins. Matrices from two biological replicates have been averaged to produce matrices (A) and (B). White lines are bins with too-low read coverage excluded from analysis by hypergeometric optimization of motif enrichment (HOMER). Arrow 1, 3′ CBEs; arrow 2, Eμ; arrow 3, IGCR1; arrow 4, most proximal V_H genes; arrow 5, 5′ of 7183 V_H gene family; arrow 6, 5′ of S107 V_H gene family; arrow 7, 3′ J606 V_H genes; arrow 8, start of distal subdomain; arrow 9, end of distal subdomain. The positions of subdomains in pro-B cells determined using HiCseg are indicated by gray rectangles. See also Figures S2 and S3 and Table S2.

respectively) and in wild-type thymocytes (Figure 2B) at 20-kb resolution. These were complemented by distance-corrected matrices (Figures S2C and S2D). The J_H, D_H, and V_H genes and regulatory elements within each 20-kb bin are listed in Table S2.

The contact matrix in pro-B cells revealed substantial enrichment for interactions of two regulatory elements, the 3′ CBEs and the IGCR1, (Figure 2A, arrows 1 and 3, S2B, and S2C) with the entire V_H region (Figure 2A, arrows 4–9), visualized by two red stripes of high interaction frequency. Together with a high frequency of other interactions throughout the *Igh* locus (Figure 2A), which exceeds that expected from genomic proximity (Figure S2C), this suggests that pro-B cells are a heterogeneous ensemble and individual cells harbor distinct *Igh* conformations. The high density of the data suggests that individual *Igh* loci undergo looping of multiple different V_H genes, and this might underpin the high diversity of V(D)J recombination products. The pattern of interactions in pro-B cells strongly suggests that they are mediated by CTCF. The multiple sites comprising the 3′ CBEs, a proposed superanchor,[12] are in convergent orientation with ~110 upstream CTCF sites in the V_H region, as is one of the two CBEs in the IGCR1,[8,52] while the other is convergent with the 3′ CBEs (Figure 2A).[4,53] The anchor-like interaction profiles of the 3′ CBEs and the IGCR1 support recent reports of CTCF-mediated loop extrusion between these elements and the V_H region.[47–49]

The 3′ CBEs and the IGCR1 interact frequently with each other (Figures 2A, S2B, S3A, and S3D, arrows 1 and 3) and with the genes between these elements, forming a highly looped 3′ subdomain, supported by 2D segmentation of the HiC data using the HiCseg TAD caller (Figure 2A, S2A, and S2C). This subdomain nevertheless interacts frequently with the next subdomain, which contains the 3′ end of the V_H region (Figures 2A, arrow 4, and S3E), including the most highly recombining genes 7183.2.3 (V_H81X) and Q52.2.4.[4,53] Frequent contacts continue over the proximal V_Hs to the end of the V_H7183 gene family at 113.89 Mb (Figure 2A, arrow 5). Together these two subdomains may be nested in a larger subdomain.

The 3′ RR, an enhancer important for class switch recombination (CSR),[54,55] interacts with the 3′ CBEs but does not contain CTCF sites and does not interact frequently with the V_H region (Figures 2A and S3B). Rather, it interacts with the neighboring bins containing constant genes that will subsequently participate in CSR. The latter are not involved in frequent contacts with other *Igh* elements and may be looping out of the 3D structure of the locus (Figure 2A, between arrows 1 and 2).

Surprisingly, relatively few interactions were captured from the Eμ enhancer with the rest of the *Igh* locus (Figures 2A, arrow 2, and S3C). Eμ is the only *Igh* enhancer required for V(D)J recombination,[56–58] is continuously transcribed in pro-B cells,[59] and promotes permissive chromatin in the *Igh* locus.[29] It has also been implicated in intralocus interactions and locus compaction,[42,60] but this remains subject to debate.[40] Nevertheless, since all parts of the *Ig* loci were evenly covered with baits (Figure S1D), and CHi-C and Hi-C libraries exhibited the same read coverage pattern across the *Igh* locus (Figures S1E and S1F), we consider that the low number of interactions with the Eμ enhancer within the *Igh* locus, although surprising, is a validated finding. To confirm this, we scrutinized relevant published Hi-C[12,41,48] and 4C[40] datasets and found that these also demonstrated reduced interactions of Eμ with the *Igh* locus. Our findings here suggest that Eμ interactions may be too infrequent and too short lived to be captured.

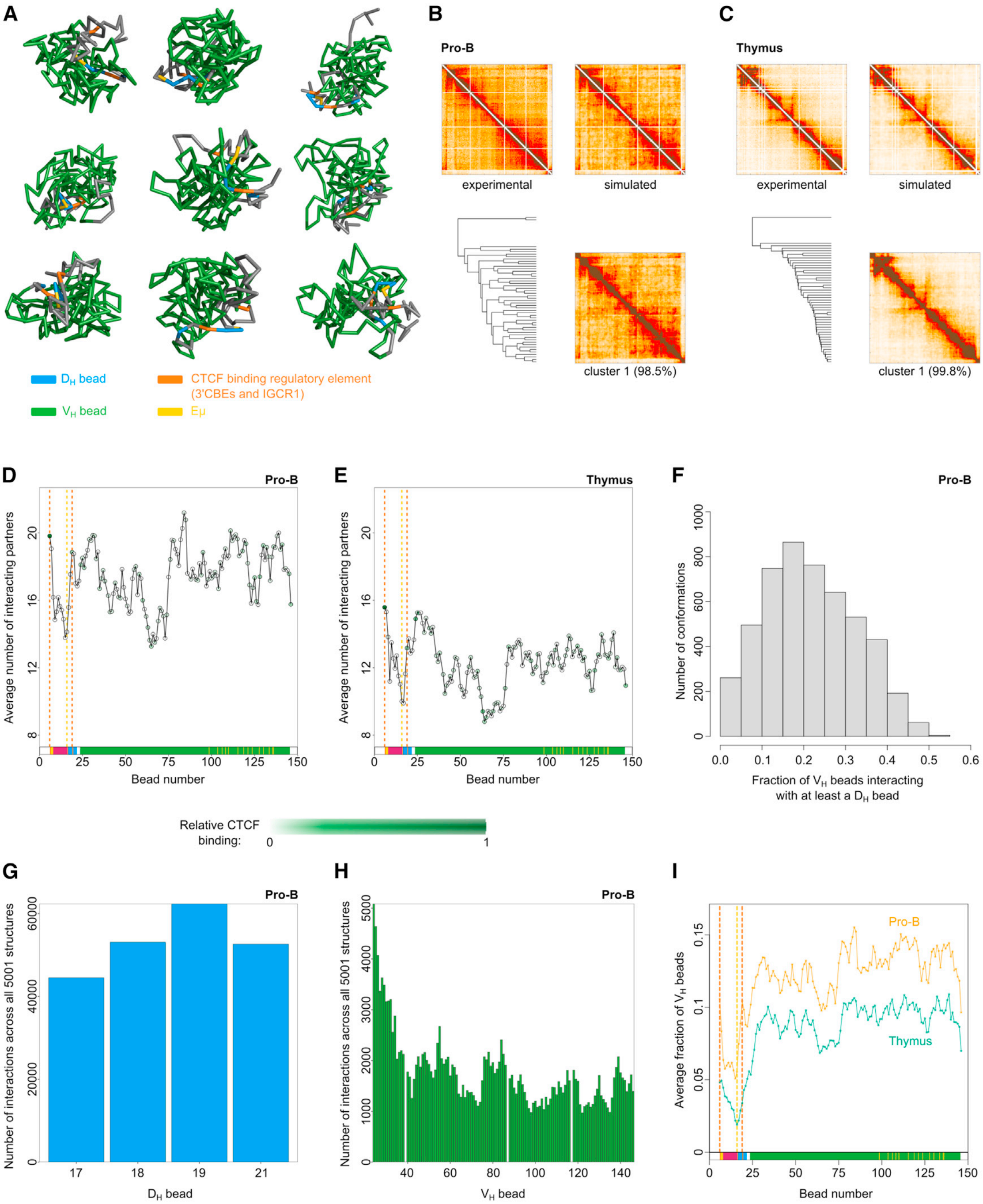

Figure 3. The individual spatial conformations of the Igh locus are a heterogeneous ensemble in pro-B cells

(A) A random selection of nine out of 5,001 simulated conformations of the *Igh locus* in pro-B cells.

(B and C) Experimental and simulated heatmaps are shown for (B) pro-B and (C) thymus. Single structures generated by the polymer model were clustered using the root-mean-square difference in bead-to-bead distances as a dissimilarity score and the heatmap corresponding to the largest cluster is shown (B) and (C).

(D and E) The average number of interacting partners (bead-to-bead distance <1.5a) across all conformations was calculated for each bead in (D) pro-B cells and (E) thymus. CTCF-binding level is indicated by the color of the data point. Vertical dotted lines indicate the position of the 3′ CBEs (left, orange), Eμ (middle, yellow), and IGCR1 (right, orange).

(F) For each conformation in pro-B cells, the fraction of V_H beads interacting with at least one D_H bead was determined. In most conformations, over 20% of V_H beads contact a D_H bead.

(legend continued on next page)

The next two subdomains include the majority of the interspersed middle V_H gene families and the discrete J606 gene family, starting at 114.35 (Figures 2A, arrow 7, and S3F), respectively.

A striking feature of the *Igh* spatial organization in pro-B cells was a large 5′ subdomain encompassing most of the distal V_H genes (Figure 2A, between arrows 8 and 9), with interactions throughout this domain occurring more frequently than expected from genomic distance (Figure S2C). It starts at 114.61 Mb, just upstream of the J606 and V_H10 gene families (Figure S3G), and ends after the last V_H gene at 116.03 Mb. The enrichment of interactions within the distal V_H subdomain is exemplified by the interaction profile of the bin containing J558.71pg.172 and J558.72.173 V_H genes (Figure S3H).

In wild-type thymocytes, the long-range interactions of the 3′ CBEs and IGCR1 with all V_H genes were absent (Figure 2B and S2D). However, the 3′ subdomain, previously shown by 3C,[60] was already present. This indicates early locus organization, consistent with preparation for *Igh* D_H to J_H recombination, which occurs on up to 50% of alleles in thymic T cells.[61,62] Frequent local short-range contacts along the V_H region were also detected, which were very similar to V_H contacts observed by FISH in Rag-deficient TCRb transgenic thymocytes.[62] We used wild-type thymocytes because Rag-deficient thymi lack CD3 T cells, and we infer that localized V_H structure is established during thymocyte development, independently of *Igh* D_H to J_H recombination.

Collectively, these findings reveal important roles for both the 3′ CBEs and the IGCR1 in *Igh* locus structure. Their bipartite interactions create a functional 3′ subdomain (Figure 2A, second gray bar), while their frequent interactions with all V_H genes in pro-B cells and their anchor-like interaction patterns suggest an overarching role in dynamic V_H region structure, mediated by CTCF.

These insights into the organization of the entire *Igh* locus provided by CHi-C, at the highest resolution to date, unite the seemingly divergent models based on a flexible continuum of loops[40] and three subdomains.[43] CHi-C has readily detected both the rich looping landscape between the V_H genes and the 3′ CBEs and IGCR1 anchors, as well as the presence of 3′ and 5′ subdomains.

Polymer modeling reveals a heterogeneous ensemble of *Igh* single conformations

Hi-C provides a view of genome organization averaged over millions of single-cell conformations. However, it does not provide direct access to the cell-to-cell variability in chromosome folding. Thus, whether the *Ig* loci exhibit considerable individual conformational diversity in order to ensure participation of multiple V_H, D_H, and J_H gene segments in recombined V(D)J products cannot be determined directly from Hi-C-based approaches. To understand the cell-to-cell variability of genome folding at the *Igh* locus, we applied polymer modeling to extract individual conformations from ensemble CHi-C data. The variability of the structures was parameterized using the CHi-C experimental data. We and others have shown that this polymer modeling approach correctly reproduces cell-to-cell variation in chromosome structure measured experimentally.[63–65]

We employed coarse-grained polymer models where each monomer (bead) corresponds to 20-kb bins in the *Igh* baited region. Interaction energies between monomers were inferred to reproduce the experimental heatmaps (Figure S4A). The optimal model was then used to generate an ensemble of representative single-cell conformations (Figure 3A). By clustering similar conformations from the polymer model in terms of root-mean-square displacement, we showed that conformations were highly heterogeneous and no dominant cluster of similar conformations exists in either pro-B cells or thymocytes (Figures 3B, 3C, S4B, and S4C). The average number of beads that each *Igh* bead contacted ($\leq$1.5a; a = 20-kb heatmap bin) in pro-B cells was 13–22, while in thymocytes it was 8–16 (Figures 3D, 3E, S4D, and S4E). This indicates that the conformations in pro-B cells are more compact and have many more interactions.

In line with population-averaged CHi-C contact patterns, in pro-B cells, the 3′ CBEs (bead 6) and the IGCR1 (bead 19) were among the most interactive beads at the 3′ end of the locus, whereas beads 9–16 containing the constant genes and the Eμ were the least interactive (Figures 3D, 3E, S4D, and S4E). In the V_H region, proximal and distal V_H beads were more interactive than middle V_H beads (beads 61–74). In the thymus, the pattern of interactivity of each bead was similar to that in pro-B cells, albeit with overall reduced numbers of interacting partners and a small bias in favor of proximal V_H genes.

On average, 20% of the 123 V_H-gene-containing beads interacted with at least one D bead in any single structure in pro-B cells (Figure 3F). These V-D interactions were distributed evenly among the four D_H beads, including bead 21, which is positioned 5′ of the IGCR1 (Figure 3G). They were also distributed throughout the V_H beads, albeit with a proximal bias (Figure 3H). These data show that the 60-kb D_H region contacts multiple V_H genes simultaneously, in agreement with previous findings that multiple V_H genes contact the DJ_H region in a confined space.[38] Individual V_H beads interacted with 10%–15% of other V_H beads on average in pro-B cells in every single conformation (Figure 3I). Interestingly, the V_H beads exhibited a similar pattern of contacts with other V_H beads in the thymus, albeit with decreased frequencies (Figure 3I). This suggests a pattern of pre-existing folding in the V_H region that becomes more frequent upon locus contraction. This is reflected by the 3′ CBEs and the IGCR1 beads in the thymus contacting the V_H beads much less frequently than in pro-B cells. The Eμ exhibited very low interaction frequency with V_H beads in both cell types (Figure 3I), confirming our previous observations. We did not observe a correlation between V-D interaction probability and V_H gene recombination frequency, either when

(G and H) The number of V_H-D_H interactions across the 5,001 pro-B conformations per D_H bead (G) and V_H bead (H).

(I) The fraction of V_H gene beads contacted by each bead in each conformation, averaged across all 5,001 conformations for pro-B (orange) and thymus (green). Vertical dotted lines as for (D) above.

See also Figure S4.

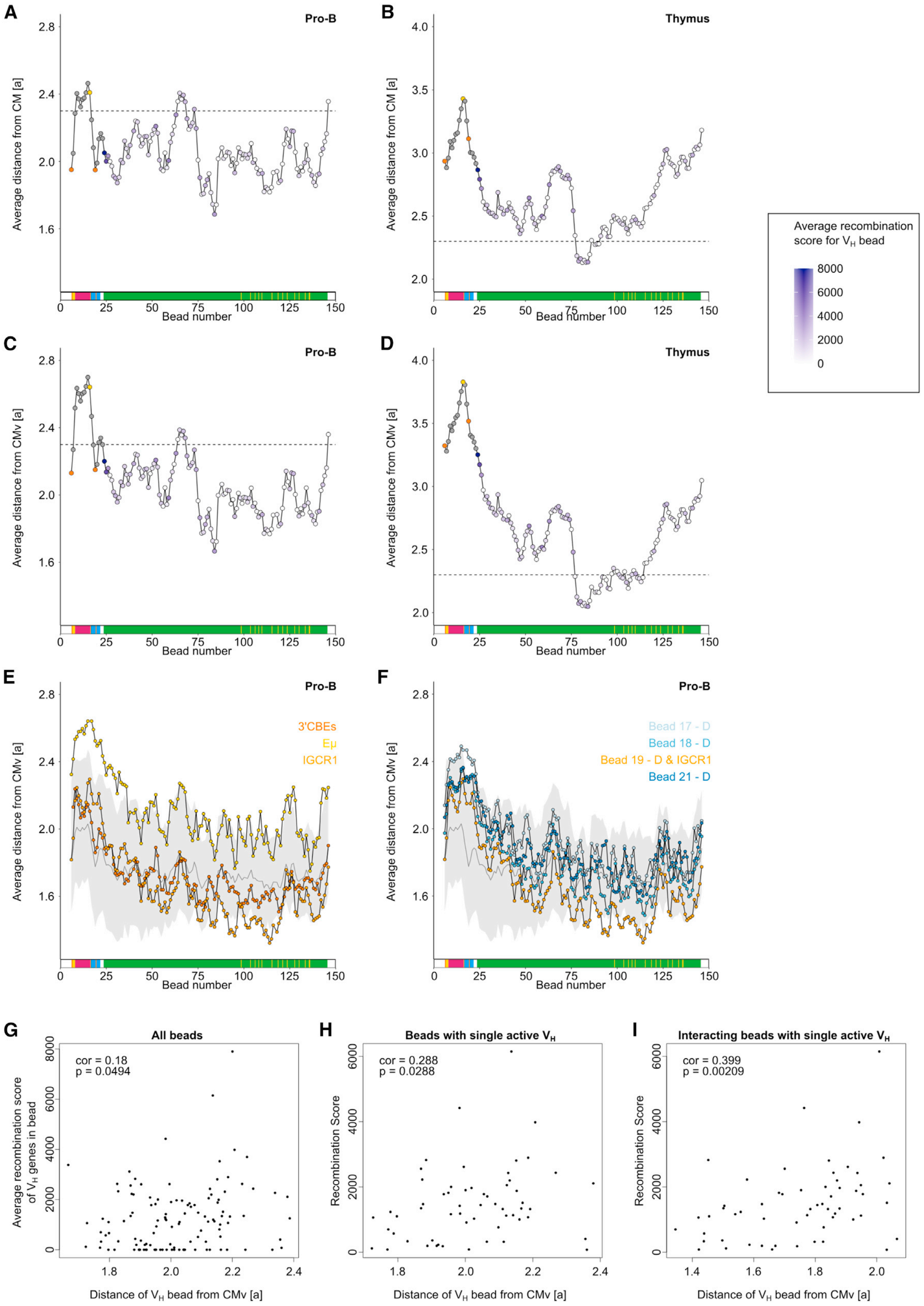

(legend on next page)

looking at all V_H genes within every bead or when considering only beads containing a single actively recombining V_H gene (Figures S4F and S4G). This suggests that the frequency of V-D interaction events is sufficient not to be a rate-limiting step in recombination of individual V_H genes, even those furthest from the DJ_H region in linear DNA sequence. Additional features, including local chromatin state at V_H genes, influence efficiency of recombination.[4,66] Our findings of diverse, highly interactive individual structures indicate that all V_H genes have equal opportunity to interact with the D_H region, and thus poor recombination efficiency of individual V_H genes primarily reflects suboptimal local V_H chromatin state.

CTCF binding in the V_H gene beads did not significantly affect their interaction probability with a D bead (Figure S4H) or recombination score (Figures S4I and S4J), despite different patterns of local and intergenic binding in the 3′ and 5′ regions, respectively. Rather, the converging elements at the 3′ end of the locus bound by CTCF (3′ CBEs and IGCR1) show an anchor-like interaction pattern with all V_H genes via more than 100 converging CTCF sites in the V_H region, suggesting that CTCF binding is a major contributor to the equal opportunity for all to recombine, but additionally, local binding of CTCF or Pax5 is required for efficient recombination.[4]

Interactions within the *Igh* locus are focused over distal V_H genes

The current model of *Igh* structure proposes that interactions are focused on the recombination center, where the RAG complex first binds D_H and J_H genes in the 3′ subdomain and D_H-J_H recombination takes place,[67] with V_H genes looping toward this focal point. A notable advantage of polymer modeling of individual *Igh* structures is that we could investigate where in the single structures the V-D contacts could take place. To this end, we used as reference the center of mass (CM) and the CM of V_H genes (CMv) in the *Igh* structures in pro-B cells (V_H genes are in beads 24–146) (Figures 4A–4D). This is related to the "average" position of V_H genes in single structures. First, we observed that, in pro-B cells, the 3′ CBEs and IGCR1 were the closest non-V_H elements to the CMv, with C, J, and Eμ being the farthest (Figure 4C). Next, we looked more closely at the geography of V-D interactions within single structures with respect to the CM. Interaction with the 3′ CBEs or IGCR1 reduced V_H bead distance from CMv, whereas interaction with Eμ increased distance (Figure 4E). Despite the reduction in distance from CMv for V_H beads interacting with bead 19, which contains D genes in addition to the IGCR1, interaction with the other D beads did not substantially affect V_H bead localization; indeed, if anything, these interactions occurred slightly further than average from the CMv (Figure 4F). Overall, the distance of V_H-gene-containing beads from the CMv was not correlated with V_H gene recombination score (Figures 4G and 4H). However, when we considered only single simulated structures in which a bead containing a single V_H is engaged in a V-D interaction, there was a positive correlation with recombination frequency (Figure 4I). This suggests that V_H genes positioned toward the periphery of the V_H domain may have a greater opportunity to engage in a V-D interaction that leads to recombination.

In summary, the simulation data revealed that there is no single or highly prevalent conformation of the *Igh* locus in 5,000 structures, thus revealing that the vast majority of looping landscapes in single B cells are unique. This extraordinary variety is very likely to underpin antibody diversity. Moreover, V-D interactions resulting in recombination tend to occur toward the periphery of the compact V_H region, and V_H usage frequency and V-D interactions are not highly correlated. Together with a high frequency of V-D interactions (20% of V_H beads interacted with a D_H bead in each single structure), these findings indicate that this is not a rate-limiting step overall and further strengthens the "equal opportunity for all" model.

A network of interchromosomal interactions enriched in B lineage genes may drive B cell development

There is growing evidence supporting functional contacts between genomic regions on different chromosomes, detected by chromosome conformation capture and equivalent methods.[68–75] However, others have struggled to detect such contacts during lymphocyte development, highlighting technical challenges impeding reliable identification of *trans* interactions.[76]

The enrichment afforded by CHi-C enabled investigation not only of interactions within the baited *Igh* locus but also of genome-wide contacts of the *Igh* and other enriched regions (Figure 1A). To identify *trans* contacts of the *Igh* locus, we extracted read pairs with at least one end mapping to a baited region and the other end mapping to a different chromosome. We performed virtual 4Cs (V4C) from *Igh*, *Igk*, *Igl*, *Pax5*, *Foxo1*, *Ebf1*, *Runx1*, *Bach2*, and *Il7r* in Rag1$^{-/-}$ pro-B, Rag/81X pre-B, and thymocytes and quantified the number of *trans* reads in 500-kb bins genome-wide (Table S3). This large bin size was necessary because interchromosomal contacts are much less frequent than *cis* interactions and quantification at higher resolution reduced signal-to-noise ratio. The V4C from the *Igh* viewpoint showed high signal-to-background ratio with sharp interaction peaks

Figure 4. Distance from the CM reveals *Igh* structure is focused on the V distal region

(A–D) Average distance from CM (a) across all conformations was calculated for each bead in pro-B cells (A) and thymus (B). Similarly, average distance from CMv (a) across all conformations was calculated for each bead in pro-B cells (C) and thymus (D). The color of V_H bead data points indicates the average recombination score of V_H genes in the bead. The positions of the 3′ CBEs (left, orange), Eμ (yellow), and IGCR1 (right, orange) are indicated; other non-V_H beads are gray.

(E and F) Average distance from CMv (a) for each bead in the subset of structures in which it is interacting with each of the beads named top right (color corresponds to the color of the points). Gray line shows the median distance from CMv across the subsets of structures in which a given bead is interacting with each of the other beads; gray ribbon indicates the range encompassing 75% of these subsets of structures.

(G–I) Correlation between distance of V_H bead from CMv and recombination score[4,66]). (G) For all V_H beads, the correlation between distance of V_H bead from CMv and the average recombination score of all V_H genes in that bead was determined. (H) For V_H beads containing a single active V_H gene, correlation between distance of V_H bead from CMv and the recombination score of the V_H gene. (I) As in (H), but instead of using all conformations, the average distance in a subset of conformations where the V_H is implicated in a V_H-D_H interaction was used. cor, Spearman's correlation coefficient, >0.4 equivalent to moderately strong correlation; p value < 0.05 statistically significant.

CellPress
OPEN ACCESS

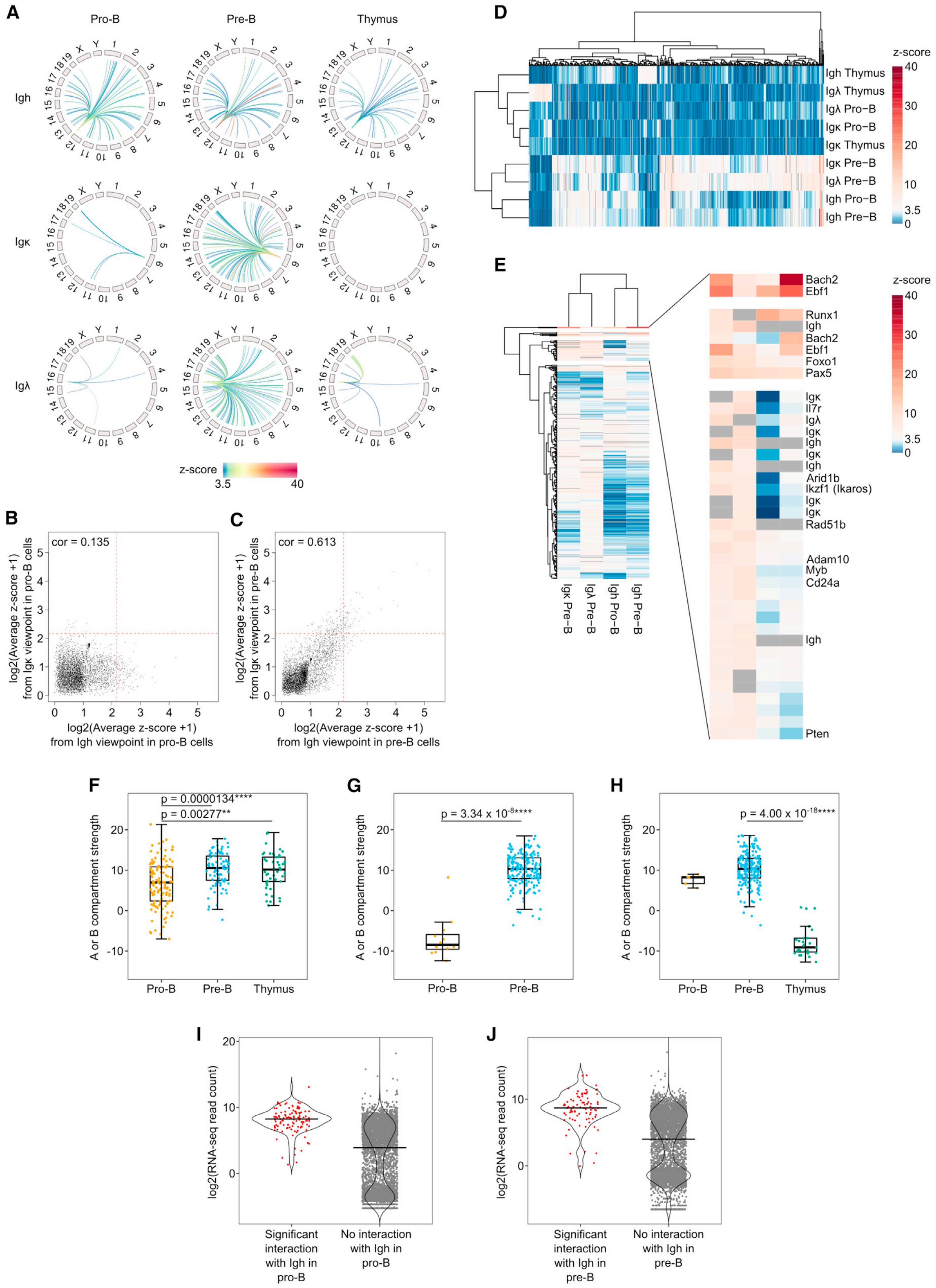

(legend on next page)

(Figures S5A and S5B). V4C from this viewpoint in Rag/81X pre-B cells (Figure S5B) identified the previously unknown integration site of the V_H81X transgene at around chr16:82,950,000–83,000,000.

To call statistically significant *trans* interactions, we performed *Z* score analysis with a cutoff of 3.5 (Table S3). In Rag1$^{-/-}$ pro-B cells, the *Igh* participated in 116 interchromosomal interactions (Figures 5A, S5C, and S5F), whereas the *Igk* and *Igl* loci made few contacts (Figures 5A and S5D–S5F).

Strikingly, the bins contacted most frequently in *trans* by the *Igh* viewpoint in pro-B cells contained genes crucial for B cell development, including *Runx1*, *Ebf1*, *Pax5*, *Aff3*, *Cux1*, *Foxp1*, *Pik3r1*, *Foxo1*, *Bach2*, *Zfp36L2*, *Hmgb1*, *Runx3*, *Lef1*, and *Akt3* (Figure 5A; Table S3). In contrast, *Hist1* (Figure 1D) was not contacted. Importantly, the read counts for baited-to-baited and baited-to-non-baited interactions were in the same range, indicating no detection bias in favor of baited-to-baited *trans* interaction reads. Accordingly, for the *Igh* viewpoint in pro-B cells, only five other baited regions (*Runx1*, *Ebf1*, *Pax5*, *Foxo1*, and *Bach2*) were among the 116 statistically significant interaction partners. These key B lineage-associated transcription factor genes, in addition to *Il7r*, themselves participated in numerous *trans* interactions in pro-B cells (Table S3). Importantly, *Igh* did not contact the *Igk* and *Igl* loci in pro-B cells, suggesting spatial separation of active and inactive *Ig* loci.

In Rag/81X pre-B cells, the light-chain loci become active and gain numerous *trans* interactions, including with the *Igh* locus (Figure 5A, S5D, S5E, and S5G). Twenty-eight interaction partners were shared between all three *Ig* loci, and *Igk* had 29 and 82 additional shared contacts with *Igh* and *Igl*, respectively (Figure S5G). Accordingly, *Z* scores for *trans* interactions correlated poorly between *Igk* and *Igh* in pro-B cells (Figure 5B) but very strongly in pre-B cells (Figure 5C). Most viewpoints retained a majority of their contacts and gained new contacts in pre-B cells. Accordingly, the *trans* interactions made by the *Igh* locus overlapped significantly in pro-B and pre-B cells (Figure S5C), and *Igh* retained 18 of its top 20 pro-B cell interactions. Moreover, in pre-B cells, 16 of these overlapped with *Igk* contacts (Figures 5D, 5E, S5G, S5I, and S6). This suggests that, upon activation, the light-chain loci join the interchromosomal interaction network that connects the *Igh* and other viewpoints in pro-B cells.

The interchromosomal contacts in thymocytes were in marked contrast (Figures S5C and S5H): *Igh* only contacted genes expressed in both B and T cells, including Runx1 and Pik3r1[77–79] (Table S3), while *Igl* made very few contacts and *Igk* made none (Figures 5A, S5E, and S5H).

Since the *Ig* loci contacted key B lineage genes frequently, and they in turn had many interchromosomal interactions, we next sought to determine the level of interdependence of these interactions. Figure S5I shows that there are many shared interactions between the *Ig* loci and three key transcription factor genes: *Pax5*, *Ebf1*, and *Foxo1*. Notably, however, the majority of transcription factor interactions do not involve the Ig loci, suggesting a wider network of interchromosomal interactions.

Interestingly, many viewpoints gained very frequent interactions with the *Bach2* locus in pre-B cells, coinciding with its activation at this stage[80] (Figure S5I; Table S3). Similarly, the *Foxo1* gene locus[81] exhibited many significant interactions in thymocytes, including with *Bach2*, *Foxp1*, *Runx1*, *Ly86*, *Il10*, *Cux1*, and *Aff3*, consistent with its role as a key transcription factor in T cell development[82] (Table S3). Crucially, while *Foxo1* interacted with *Pax5* and *Ebf1* in B cells, it lacked these interactions in thymocytes, indicating exquisite lineage specificity of its interactions. Thus, interchromosomal interactions are cell type and developmental stage specific.

Clustering analysis revealed that the *Bach2* and *Ebf1* loci in pre-B cells were the most interactive, and their contact profiles closely resembled those of the *Pax5* and *Foxo1* loci in pre-B, the *Igh* locus in pro-B and pre-B cells, and *Runx1* in all three cell types (Figure S5I).

We next assessed the chromatin environment of regions participating in *trans* contacts by using non-enriched Hi-C datasets to assign active A and inactive B compartments.[18] The vast majority of regions interacting with the *Igh* in pro-B and pre-B cells, or with *Igk* or *Igl* in pre-B cells, resided in A compartments (Figures 5F–5H). The *Pax5* locus was in an A compartment in all three cell types, including thymocytes, where it does not contact the *Ig* loci (Figure S5M). However, the compartment state of other interaction partners including *Ebf1* was developmental stage specific (Figure S5N). The *Igh* locus itself resided in the A compartment in pre-B cells and thymus. Surprisingly this was less evident in pro-B cells (Figure S5J). Bins containing *Igk* and

Figure 5. The *Ig* loci participate in shared, developmental-stage-specific interchromosomal interactions

(A) Statistically significant (*Z* score > 3.5) interchromosomal interactions are plotted for each *Ig* viewpoint (*Igh*, top; *Igκ*, middle; *Igλ*, bottom) in pro-B cells (left), pre-B cells (middle), and thymocytes (right). Gray rectangles denote chromosomes and arcs denote *trans* interactions, with highest *Z* scores in red. Full list of *Z* score values is in Table S3B.

(B and C) Log2(average *Z* score +1) was calculated for each 0.5-Mb bin genome-wide for the *Igh* viewpoint and was compared with the corresponding values for the Igκ viewpoint in (B) pro-B and (C) pre-B datasets. cor, Spearman correlation coefficient. Dotted red lines indicate the threshold of a statistically significant *Z* score.

(D) 0.5-Mb bins were selected for hierarchical clustering if they had a significant interaction with any of the three *Ig* loci at any developmental stage. Clustering, based on average *Z* score values, was performed using the pheatmap package in R.

(E) The 0.5-Mb bins were selected for hierarchical clustering if they had a significant interaction with any of the *Ig* loci at a developmental stage permissive for recombination or recombined. Clustering performed as in (D).

(F–H) For the *Igh* (F), *Igκ* (G), and *Igλ* (H) viewpoints, boxplots show the A or B compartment strength for 0.5-Mb bins that are significant interaction hits at each developmental stage. Values > 0 indicate A compartments and values < 0 indicate B compartments. In (F) and (H), statistically significant differences were determined using a Kruskal-Wallis test followed by post hoc testing for pairwise comparisons using the dunn.test function with Bonferroni correction. For (G) a Mann-
Whitney U test was used.

In pro-B cells (I), and pre-B cells (J) the amount of transcription, measured by log2 (average RNA-seq read count normalized per million; Table S4) was compared between bins participating and not participating in significant interaction with the Igh locus. See also Figures S5 and S6.

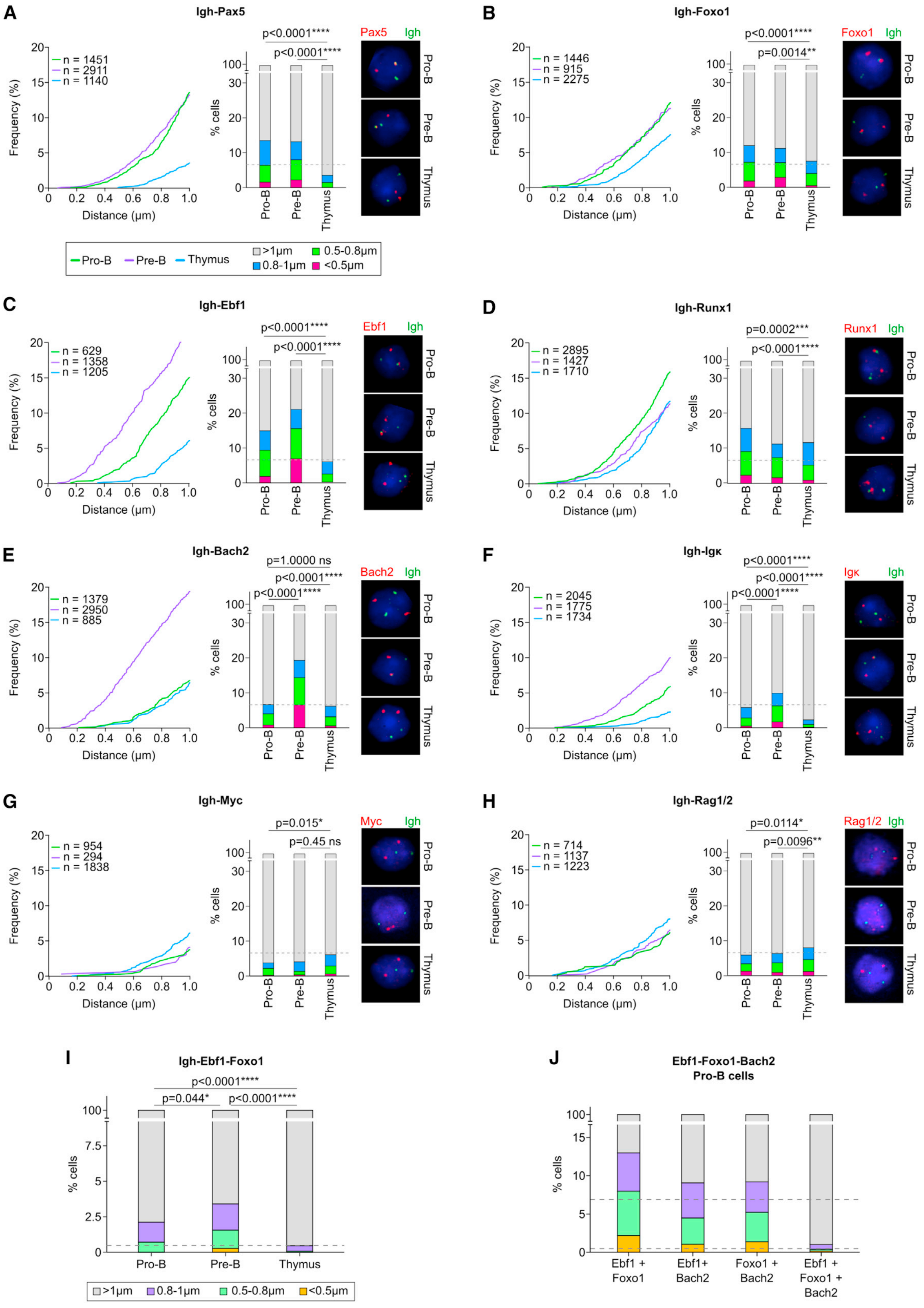

(legend on next page)

Igl resided in A compartments in pre-B cells and in B compartments in pro-B cells and thymus (Figures S5K and S5L); these active or repressive chromatin environments were largely mirrored in the bins they contacted (Figures 5G and 5H), in line with spatial segregation into active and inactive chromatin.[18]

We observed *trans* contacts predominantly between active chromatin compartments. Transcriptional activity has been linked to chromatin contacts.[19,70] Using nuclear RNA sequencing (RNA-seq) to enrich for primary transcripts, we found that the transcriptional activity in 500-kb bins interacting with the *Igh* locus corresponded to a subset of actively transcribed genes (Figures 5I and 5J; Table S4), including those with B cell-specific functions such as *Pax5* and *Ebf1*. However, other more highly expressed housekeeping genes, including *Malat1*, *Hist1*, and *Actb*, did not make *trans* contacts with the *Igh*. Genes including *Bach2*, *Arid1b*, *Adam10*, and *Zfp36l1* were more highly expressed in pre-B cells and this coincided with gain of interactions with the Ig loci. In summary, we have uncovered a developmental-stage-specific interchromosomal network of active B lineage-specific genes.

The *Igh* locus is in spatial proximity to key B lineage genes more frequently in B cells than in thymocytes

To validate the top interchromosomal interactions detected by CHi-C, we performed 3D DNA FISH and imaged contacts of the *Igh* and *Igk* loci with *Pax5*, *Ebf1*, *Foxo1*, *Runx1*, and *Bach2* genes and with each other. *Myc* and *Rag1/2* were negative controls, since these regions did not show *trans* contact by CHi-C. We observed a significantly higher proportion of distances shorter than 1 μm between the *Ig* loci and the genes of interest in Rag1$^{-/-}$ pro-B cells and/or Rag/81X pre-B cells compared with the random probability of association (6.9%) (Figures 6A–6H and S6F–S6I; Table S5). This was not the case in thymocytes, with the exception of *Runx1*. In agreement with CHi-C, the *Igh+Pax5* and *Igh+Foxo1* contacts had similar frequencies in pro-B and pre-B cells (Figures 6A and 6B). The *Igh+Ebf1* contacts showed a larger number of distances <1 μm in pre-B cells than in pro-B cells, consistent with higher *Z* scores of *Ebf1*-containing bins (Figure 6C). *Runx1* interaction with the *Igh* was confirmed in all three cell types (Figure 6D). *Bach2* and *Igk* were in frequent proximity to the *Igh* only in pre-B cells (Figures 6E and 6F). *Igh+Myc* and *Igh+Rag1/2* showed a low frequency of distances <1 μm in all three cell types (Figures 6G and 6H), and independent FISH experiments showed concordant results (Figures S6A and S6B). More detailed analysis of distances <0.5 and <0.3 μm (Table S5) reflected the patterns observed in Figures 6 and S6. Contact partners of the *Igk* showed frequent short distances in pre-B cells but not in pro-B and thymocytes, confirming that *Igk* only participates in *trans* interactions when it is active (Figures 6F, S6F, and S6I). Additionally, we detected significant three-way associations of *Igh*, *Ebf1*, and *Foxo1* in pro-B cells and, with increased frequency, in pre-B cells (Figure 6I). We also detected *Igh*-independent two-way and three-way associations of *Ebf1*, *Foxo1*, and *Bach2* in pro-B cells (Figure 6J), validating *Ig*-independent interactions detected by CHi-C (Figures S5I and S6J). Strikingly, although we only show two examples of three-way associations here, most two-way associations had frequencies of 10%–20%. Igh interactions with just six genes amounted to 80% of alleles (Figure 6). This high frequency is inconsistent with a model of exclusive pairing. Given Igh interacts with many other genes in Chi-C, and the many *Ig*-independent associations we have observed (Figures S5I and S6J), we infer that it is very likely that networks will include many three-way and higher-order interactions.

Overall, FISH experiments validated CHi-C findings that the Ig loci participate in *trans* interactions in a developmental-stage-specific and cell-type-specific manner.

Last, we examined at higher resolution the regions of the *Igh* locus that participated in *trans* interactions. Regions that were most peripheral in simulated single structures (beads 9–16, 113,270,000–113,429,999; and 64–68, 114,370,000–114,469,999; Figure 4A) were most frequently contacted *in trans* (Figure 7A), suggesting that these regions favored *trans* interactions over participation in V(D)J recombination.

In summary, these newly identified interchromosomal associations suggest that the *Ig* loci participate in interactions in *trans* with multiple genes driving B cell development and form highly specific co-regulatory or co-transcriptional spatial networks. Chromatin structure or transcription status alone cannot fully explain these networks, suggesting an additional layer of specificity that may underpin these networks and B cell identity (Figure S7).

DISCUSSION

The diversity of the Ig repertoire is a key pillar of the adaptive immune response. It depends firstly on the variety of V(D)J

Figure 6. The *Igh* locus is in spatial proximity to key B lineage genes more frequently in B cells than in thymocytes
(A–H) Two-color 3D FISH probing of the *Igh* locus and either (A) *Pax5*, (B) *Foxo1*, (C) *Ebf1*, (D) *Runx1*, (E) *Bach2*, (F) *Igκ*, (G) *Myc*, or (H) *Rag1/2* loci was performed in pro-B cells, pre-B cells, and thymocytes. Line graphs show cumulative distributions of distances <1 μm between the Igh locus and genes of interest in three cell types. The closest signal pairs in each cell were used. n = number of nuclei analyzed. Bar graphs show distribution of distances grouped into four brackets (<0.5, 0.5–0.8, 0.8–1, and >1 μm). Dashed line denotes the probability of a random interaction at a distance of <1 μm of 6.9%. The p values were calculated using the Fisher's exact test with Bonferroni correction. Representative images of single nuclei are shown. The nuclear area was stained with DAPI, the *Igh* probes were labeled with Alexa Fluor 488 (green), and probes for genes of interest were labeled with Alexa Fluor 555 (red). The *Igh* (DJ) probe (BAC RP23-109B20) was used for pro-B and thymus, and the *Igh* (V) probe (BAC RP23-70F21) for pre-B (compared in Figure S7; discussed in STAR Methods).
(I) Three-color 3D FISH for the *Igh*, *Ebf1*, and *Foxo1* loci was performed in pro-B cells, pre-B cells, and thymocytes. The closest signal pairs in each cell were filtered to retain only cells in which at least two of the pairwise distances connecting the closest three signals were within 1 μm. Bar graph shows the distribution of distances (<0.5, 0.5–0.8, 0.8–1, and >1 μm). Dashed line denotes the probability of a random tripartite interaction at a distance of <1 μm of 0.48%.
(J) Three-color 3D FISH for the *Ebf1*, *Foxo1*, and *Bach2* loci was performed in pro-B cells as for (I) above. Dashed lines denote the probability of a random bipartite (upper line) or tripartite (lower line) interaction as above.
See also Figure S7 and Table S5.

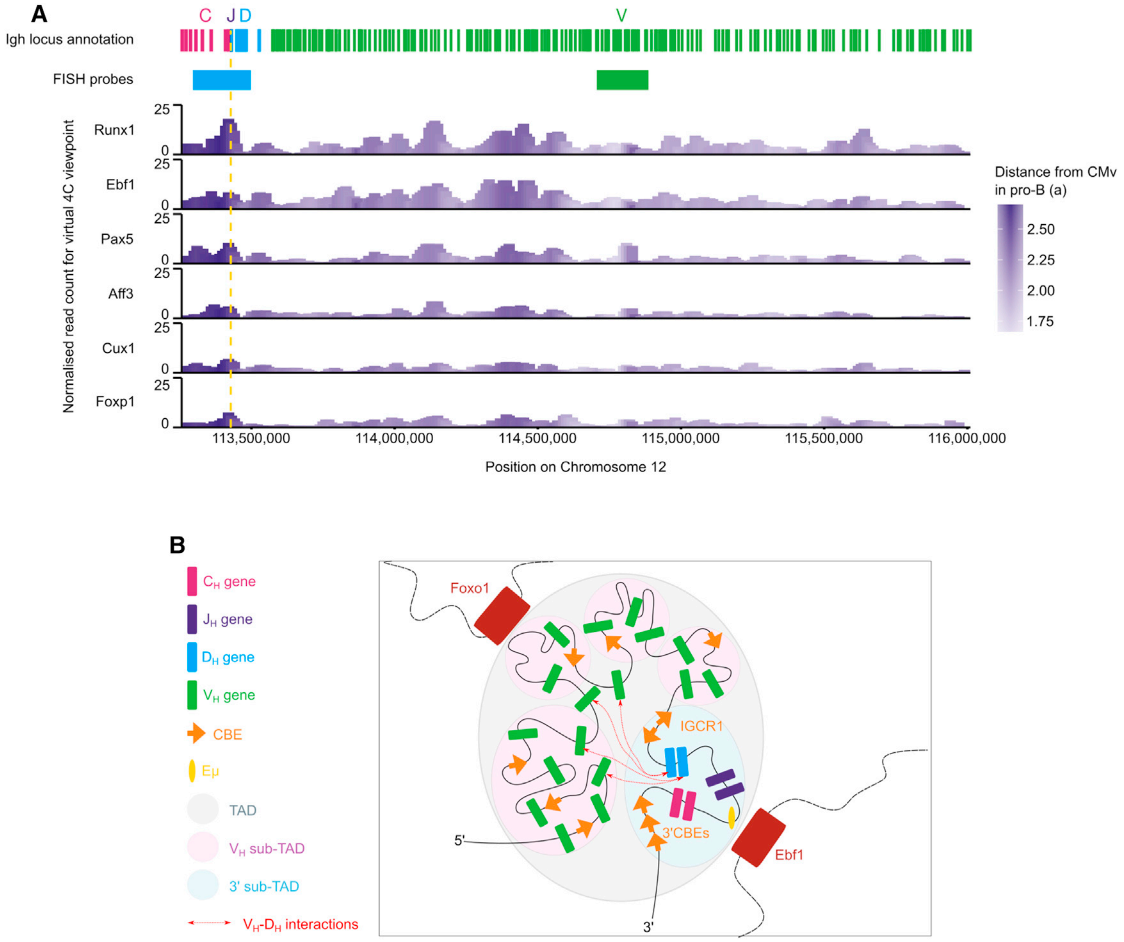

Figure 7. Peripheral regions of the *Igh* locus interact in *trans* with other loci

(A) To identify *trans* contacts of the *Igh* locus, read pairs with at least one end mapping to a baited region and the other end mapping to a different chromosome were extracted. Virtual 4C interaction profiles were generated from selected 0.5-Mb bins among the top hits of interchromosomal interactions with the *Igh* in pro-B cells. Other ends were quantified in 40-kb bins with a 10-kb step over the *Igh* locus. The positions of FISH probes used in Figure 6 and *Igh* gene segments are indicated at the top. Vertical dotted yellow line, Eμ.

(B) Organization of the *Igh* locus into sub-TADS (pink and blue circles) while a dynamic continuum of interaction occurs between the 3′ sub-TAD and the V_H region enabling diverse V_H-to-D_H recombination events (red dotted arrows). The peripheral regions of the *Igh* locus participate in interchromosomal interactions, for example with *Ebf1* and *Foxo1*.

recombination products generated by the pro-B cell population. Long-range chromatin interactions facilitate contacts between over 200 genes and several regulatory regions within the large *Igh* locus, but previous studies of their nature and frequency have been hampered by low coverage, limited viewpoints, or incomplete interrogation of intralocus contacts.[40,43,47,48] Locus enrichment techniques provide unprecedented opportunities to zoom in on Hi-C contacts.[83–85] Here, our enrichment of Hi-C libraries over the *Igh* has alleviated several challenges of probing contacts in this locus, providing an unbiased, high-resolution, all-to-all interaction map. The interaction frequency matrix in Rag1$^{-/-}$ pro-B cells revealed that the 3′ CBEs and the IGCR1 were the focal points of interactions, acting as anchors to facilitate contacts between the 3′ end and the entire V_H region, mediated by multiple convergent CTCF sites, corroborating previous identification of the 3′ CBEs superanchor by Hi-C.[12,41] The 3′ CBEs, 3′ RR, Eμ, J and D genes, and the IGCR1 form a highly looped 3′ subdomain.

Surprisingly, however, the Eμ was the least interactive regulatory element therein. Its uniform lack of contact enrichment with the V_H genes suggests it may loop out of the *Igh* structure or be less internally interactive because it resides in a very open chromatin environment, where it is continually transcribed in association with a multi-protein RNA PolII transcription factory.[86] Such large complexes can exclude solvent to form a separate gel droplet. Alternatively, it may be sequestered by the IGCR1 to insulate the J and D genes from the proximal V_H genes to ensure diverse usage of more distal V_H genes.[9] An Eμ-centric model for locus contraction has been proposed.[60] However, lower interactivity of the Eμ compared to the 3′ CBEs and IGCR1 was evident in other studies.[8,12,40,41,48] Our findings provide structural insights that support the alternative model that disputes a role

for Eμ in *Igh* locus contraction.[40] Nevertheless there is a caveat to this model, which we discuss in section "limitations of the study."

A larger 3′ subdomain stretches from the 3′ CBEs superanchor to the end of proximal V_H genes. This may be further encompassed by a yet larger subdomain containing most of the middle V_H genes. These findings consolidate several previous studies that suggested the existence of 3′ subdomains of varying coordinates.[40,43,60,87]

We observed extensive intradomain V_H looping within the large distal V_H subdomain (114.61–116.05 Mb) in thymic non-B cells, suggesting that local *Igh* V_H region looping is established before the B lineage diverges, despite the *Igh* V region being sequestered at the nuclear periphery in T cells.[32]

These data reconcile previously observed local interactions in the distal V_H subdomain even in the absence of locus contraction,[40] with the locus contraction-dependent (Pax5-dependent) extremely long-range loops between 115.10 and 116.87 Mb (mm9; 113.86–115.64 Mb in mm10 here),[43] and with a set of complementary YY1-dependent long-range loops.[40,42] Thus, our findings unify two opposing models: hierarchical three-domain configuration[42,43,60] and flexible interactions of all V_H genes with the D-J region[40] via the 3′ CBEs superanchor[12] revealing that both occur.

Locus contraction that brings distal V_H genes closer to the 3′ end may be physically mediated by loop extrusion, which, in the *Igh*, is dependent on convergent CTCF sites.[49,88] Moreover the ∼110 CTCF sites in the V_H region could facilitate many different contact possibilities, and disruption of their orientation inhibits recombination of affected V genes,[88] similarly to effects on transcription.[89] The many different structures that we have observed with polymer modeling suggest a model of widespread and multiple loop extrusion events within individual *Igh* loci,[90] facilitated by dynamic CTCF and cohesin-driven folding.[91] Further, our findings support the current model that cohesin- and CTCF-regulated loop extrusion and RAG scanning are related processes.[92] Nevertheless, the interaction matrix profiles and single structures (discussed below) did not reveal interaction patterns that precisely correlated with CTCF-binding sites but, rather, a more diffuse pattern, consistent with a recent study suggesting that the partially extruded looping state was common and the most functionally relevant.[93] Our findings provide supportive evidence for a diffusion-based mechanism,[47] particularly in the distal V_H region, where CTCF loop anchors are far from V_H genes, thus requiring additional processes to bring V_H genes close enough for RAG scanning (Figure 7B). Indeed, partial knockdown of CTCF and Rad21 abrogates preferential Rag scanning facilitated by CTCF sites close to proximal V_H genes, and it enables greater participation of distal V_H genes.[51] This may be because diffusion mechanisms remain intact. Recent modeling suggests that this function may be provided by phase separation, wherein the *Igh* is predicted to be in a weak gel state that enables active local chromatin-based crosslinking to stabilize extruded loops.[49,94] Additionally, large-scale antisense transcription in the distal V region may contribute to stochasticity of activation of V genes, similarly to protocadherin gene clusters.[59,95] Taken together, our findings suggest there must be a co-operative interplay between large-scale chromatin alterations, loop extrusion, and diffusion to achieve the multitude of unique *Igh* structures that we have observed.

Stage-specific activation of loop formation also depends on other factors, including PAX5 and YY1.[96,97] Lack of these transcription factors diminishes locus contraction and V(D)J recombination more profoundly than mutations in *Igh* regulatory elements.[40,42,44,45,56,60] CTCF is bound to the *Igh* throughout B and T cell development[6,53] and needs additional cues to exert its interaction-mediating effects. These are provided in part by dynamic expression of cohesin, recruited to Ig loci in a stage-specific manner during B cell development.[6] Additionally, the long-range looping of distal V_H genes is PAX5 dependent,[43] and it has been proposed that PAX5 mediates subdomain formation and that YY1 juxtaposes these subdomains to further compact the locus.[42] In addition to direct binding to the *Igh* locus, indirect effects, including genome-wide facilitation of loop extrusion by PAX5 through repression of cohesin release factor, Wapl,[48] may promote *Igh* locus contraction. Our findings support this model because individual polymers show that looping is not CTCF focused.

All of the above advances have strived to find static average conformations of the *Igh* locus within a population. Single-cell Hi-C supports 3D DNA folding of the genome[98,99], but current resolution is not sufficient to evaluate individual loci. Here, by applying polymer modeling to CHi-C data, we have provided a first glimpse of single conformations of the *Igh* locus that may be present in single cells. We show that the thousands of individual structures we have deconvolved from CHi-C are highly variable. Each individual *Igh* locus forms multiple loops, which collectively generate unique structures within every *Igh* allele. This again argues in favor of a flexible, rather than a more ordered, structure. We studied $Rag1^{-/-}$ pro-B cells, where the *Igh* loci are likely more similar since they are both in a poised state,[37] whereas the two *Igh* alleles are thought to have different conformations in wild-type pro-B cells undergoing recombination,[100] and thus, we would expect even greater variety in wild-type *Igh* alleles.

The simulations revealed that each V_H bead is involved in approximately the same number of contacts with the D beads when summed across all 5,001 simulated conformations. In every single *Igh* locus structure there are ∼30 simultaneous V-D interactions. This finding necessitates a step change in our thinking about how the V_H and D_H regions interact. The current concept is of a more bipartite interaction model,[38] where the D_H region interacts dynamically with whichever single V_H gene bouncing back and forth stochastically finds its way to the right environment. Our findings suggest a much greater intensity of interactions, with many V_H genes vying for interactions with available D_H genes, providing a much greater variety of stochastic opportunities. Since there are only 10 D_H genes, these interactions must also be very dynamic, as previously proposed.[38] The interaction frequency between the V_H and the D_H elements did not show strong positive linear correlation with individual VH gene recombination frequencies. This is similar to the dynamics in the *Tcrb* locus.[101] Overall, these results support the equal opportunity for all model, where no V_H gene is restricted or favored for interaction with a D_H gene for recombination, but with the additional dimension that, within that proximal space,

dynamic exchange of V_H and D_H genes may occur. This is a permissive, rather than a deterministic, feature, since V_H genes recombine with vastly different frequencies, dictated not by their linear position but by their local chromatin features.[4,66]

Our data suggest that the D_H region does not reside centrally in a cavity within the V_H gene cluster. Rather, the V_H genes form the CM of the *Igh* locus and a V-D interaction is more likely to occur near the periphery of the V_H region. This indicates that the recombination center (i.e., where the RAG complex binds and D-J_H recombination initially takes place)[67] is not interchangeable with the CM, as previously proposed, and calls into question the widely held view that the V_H genes loop toward the "central" DJ region. Rather, the direction of travel appears to be in the opposite direction; i.e., central V_H genes loop toward the V_H gene CM periphery to engage with D genes.

A new model of lineage-determining genomic interaction networks

To date, only a few examples of interchromosomal interactions have been reported,[20] precluding in-depth analysis of their functional significance or wider prevalence. The *Igh*, *Igk*, and *Igl* colocalize in transcription factories in plasma cells[102] and a few *trans* interactions of the *Igh* locus have been reported in mature B cells.[73,103–105] We have demonstrated that CHi-C can detect highly specific interchromosomal interactions between multiple baited viewpoints and the rest of the genome. We have generated a comprehensive picture of these interactions genome-wide and have shown that the Ig loci participate in frequent non-random interactions with genes driving B cell development, including *Ebf1*, *Pax5*, *Foxo1*, *Runx1*, and *Bach2*. These *trans* contacts form a developmental-stage-specific network containing the *Igh* in pro-B cells, which is joined by the light-chain loci in pre-B cells. The striking frequency of B lineage-specific genes in this network suggests a layer of lineage-specific holistic nuclear organization not previously observed. Indeed, although we have focused our exploration and validation of interchromosomal networks on the Ig loci, because this aligned with the our intrachromosomal studies, we show here that the B lineage transcription factor genes exhibit higher numbers of interactions, many independent of the Ig loci, and many with their own target genes, and thus, we propose that these, rather than the Ig loci, may be the focal point of an interaction network(s). What drives this network? We suggest coordinated activity of the master B lineage regulators above.

We found significant overlap between *trans*-interacting genes and translocations involving the Ig loci described in early B cell malignancies. Examples include Pax5, Ebf1, Aff3, Cd79b, Irf2bp2, Clec2d, Irf8, Nfkb2, and Foxp1 (reviewed in Somasundaram et al.[106]). These findings indicate that participation in a shared network of active genes may predispose these interaction partners to aberrant translocation events.[103]

It is an attractive model that interchromosomal contacts are driven by active transcription, supported by studies showing that genes loop out of discrete chromosome territories to be transcribed in shared transcription factories. Indeed, the *trans* contacts observed here involved highly transcribed genes. However, although they involved genes from every chromosome, not all highly transcribed genes interacted with the baited viewpoints, but they interacted principally with those with functional roles in lymphocyte development. This suggests that significant interchromosomal interactions are driven by additional lineage-specific epigenetic mechanisms. The interchromosomal interaction network identified here may play a functional role in connecting co-regulated or co-acting genes. For example, Ebf1 and Foxo1 bind many of the same targets,[107,108] and Pax5, Ebf1, and Runx1 have been proposed to work both in synergy[109] and in opposing fashion[110] on shared target genes. Several of these factors activate each other.[111] Our findings reveal a mechanism by which they may achieve this complex task. Physically co-localizing genes that are coordinately regulated, including the genes encoding these regulatory factors themselves, may maximize co-operative promoter binding by a shared ensemble of transcription factors.[19] The mechanisms that bring multiple genes together in *trans* and the functional implications of these interactions in B cell development remain to be determined. However, we speculate that such coordination orchestrates rapid B lineage developmental progression, and this orchestration of genomic interactions may be a widespread process in many tissues. Together, our findings of multiple unique Ig locus structures and widespread interchromosomal interactions reveal that B cell development is driven by complex genomic alterations.

Limitations of the study

(1) These studies were conducted on a Rag-deficient background, in which D to J recombination has not occurred. Thus, we cannot exclude the possibility that D to J recombination may perturb *Igh* locus structure in ways not observed here. Nevertheless, comparative studies of several parameters of *Igh* locus structure, including active histone modifications, non-coding RNA transcription, DNA looping and contraction, and diffusion dynamics, together agree that the structure of the *Igh* in the Rag-deficient background is consistent with opening up of the *Igh* V region being independent of the D to J recombination process.

(2) We used wild-type rather than Rag-deficient thymocytes for technical reasons. While we are convinced that chromatin re-organization of the V region happens in parallel with, and independently of, D to J recombination, we cannot exclude the possibility that the *Igh* D to J recombination that can occur on a substantial minority of *Igh* alleles in thymocytes may confer an altered structure via a different mechanism to that employed in Rag-deficient pro-B cells.

(3) We have proposed that *Igh* intralocus interactions involving Eμ are infrequent due to the exclusion of Eμ in a transcription factory and/or its outward focus on interchromosomal interactions. It has also been proposed[94] that Eμ is contained within a phase-separated DJ gel droplet, although it may not be closely associated therein. Nevertheless, we cannot exclude the possibility that Eμ interacts with the V region in a separate gel droplet that is refractory to detection in Hi-C fixation conditions.

(4) We have attempted to provide experimental evidence to support the simulated *Igh* structures. We performed

single-cell Hi-C in Rag-deficient pro-B cells, but, due to the highly repetitive nature of the *Igh* locus, coverage and resolution were too low to accurately deconvolute individual *Igh* alleles. This restriction also hampered high-resolution structured illumination microscopy/stochastic optical reconstruction microscopy (SIM/STORM) of Ig loci.

(5) While we provide multiple pairwise examples of gene associations with Ig loci by DNA FISH, and one example of a three-way interaction, more examples of three-way interactions would strengthen our model of gene interaction networks. Nevertheless, an Ig-loci-independent three-way example supports our model of a wider B lineage-specific interaction network.

STAR★METHODS

Detailed methods are provided in the online version of this paper and include the following:

- KEY RESOURCES TABLE
- RESOURCE AVAILABILITY
  - Lead contact
  - Materials availability
  - Data and code availability
- EXPERIMENTAL MODEL AND STUDY PARTICIPANT DETAILS
  - Mice
- METHOD DETAILS
  - Primary cells
  - Hi-C
  - Capture HiC
  - Polymer modeling
  - 3D DNA fluorescence *in situ* hybridization (FISH)
- QUANTIFICATION AND STATISTICAL ANALYSIS
  - Hi-C and Capture Hi-C analysis
  - Polymer modeling: Clustering of conformations
  - Inter-chromosomal interaction network in Cytoscape
  - RNA-seq
  - Other datasets used

SUPPLEMENTAL INFORMATION

Supplemental information can be found online at https://doi.org/10.1016/j.celrep.2023.113074.

ACKNOWLEDGMENTS

We thank Philipp Voigt for critical reading of the manuscript; Hashem Koohy, Jonathan Cairns, Peter Chovanec, and Bhupinder Virk for assistance with bioinformatics; Kristina Tabbada for assistance with sequencing; and Simon Walker for assistance with microscopy. We are grateful to Rudi Hendriks, Erasmus MC, Rotterdam for providing the Rag81X mouse strain. O.M. (1426107) and C.R. (1947339) were supported by PhD studentships from the Medical Research Council, UK. S.J.C. was supported by an Investigator Grant (Leadership, GNT1198014) awarded by the National Health and Medical Research Council (NHMRC). Research in L.G.'s lab was funded by the Novartis Foundation, the European Research Council (grant no. 759366, BioMeTre), Marie Skłodowska-Curie Innovative Training Networks (grant nos. 813327 ChromDesign and 813282 PEP-NET) under the European Union's Horizon 2020 Research and Innovation Program, and the Swiss National Science Foundation (grant no. 310030_192642). Research in A.E.C.'s laboratory was supported by grants from the Biotechnology and Biological Sciences Research Council (BBS/E/B/000C0404, BBS/E/B/000C0405, BBS/E/B/000C0427, BBS/E/B/000C0428). The Babraham Institute provides funds, through the BBSRC, for open access publication fees.

AUTHOR CONTRIBUTIONS

M.J.T.S., S.S., S.J.C., and A.E.C. conceptualized the study. S.S., M.J.T.S., and D.J.B. devised the method. O.M., C.H.R., L.S.M., B.M.J., M.J.T.S., and D.J.B. performed experiments. Y.Z. performed modeling. O.M., C.H.R., Y.Z., L.S.M., S.W.W., A.S.-P., F.K., S.A., and C.V. analyzed data. P.F., L.G., and A.E.C. secured funding and supervised research. O.M., C.H.R., L.S.M., and A.E.C. wrote the manuscript with input from all other authors.

DECLARATION OF INTERESTS

P.F. and S.S. are co-founders and shareholders of Enhanc3D Genomics Ltd.

INCLUSION AND DIVERSITY

We worked to ensure sex balance in the selection of non-human subjects. We worked to ensure diversity in experimental samples through the selection of the genomic datasets. While citing references scientifically relevant for this work, we also actively worked to promote gender balance in our reference list. We support inclusive, diverse, and equitable conduct of research.

Received: February 27, 2023
Revised: June 28, 2023
Accepted: August 18, 2023
Published: September 6, 2023

REFERENCES

1. Schatz, D.G., and Swanson, P.C. (2011). V(D)J recombination: mechanisms of initiation. Annu. Rev. Genet. *45*, 167–202. https://doi.org/10.1146/annurev-genet-110410-132552.
2. Hewitt, S.L., Yin, B., Ji, Y., Chaumeil, J., Marszalek, K., Tenthorey, J., Salvagiotto, G., Steinel, N., Ramsey, L.B., Ghysdael, J., et al. (2009). RAG-1 and ATM coordinate monoallelic recombination and nuclear positioning of immunoglobulin loci. Nat. Immunol. *10*, 655–664. https://doi.org/10.1038/ni.1735.
3. Johnston, C.M., Wood, A.L., Bolland, D.J., and Corcoran, A.E. (2006). Complete sequence assembly and characterization of the C57BL/6 mouse Ig heavy chain V region. J. Immunol. *176*, 4221–4234.
4. Bolland, D.J., Koohy, H., Wood, A.L., Matheson, L.S., Krueger, F., Stubbington, M.J.T., Baizan-Edge, A., Chovanec, P., Stubbs, B.A., Tabbada, K., et al. (2016). Two Mutually Exclusive Local Chromatin States Drive Efficient V(D)J Recombination. Cell Rep. *15*, 2475–2487. https://doi.org/10.1016/j.celrep.2016.05.020.
5. Gillies, S.D., Morrison, S.L., Oi, V.T., and Tonegawa, S. (1983). A tissue-specific transcription enhancer element is located in the major intron of a rearranged immunoglobulin heavy chain gene. Cell *33*, 717–728. https://doi.org/10.1016/0092-8674(83)90014-4.
6. Degner, S.C., Wong, T.P., Jankevicius, G., and Feeney, A.J. (2009). Cutting edge: developmental stage-specific recruitment of cohesin to CTCF sites throughout immunoglobulin loci during B lymphocyte development. J. Immunol. *182*, 44–48.
7. Featherstone, K., Wood, A.L., Bowen, A.J., and Corcoran, A.E. (2010). The mouse immunoglobulin heavy chain V-D intergenic sequence contains insulators that may regulate ordered V(D)J recombination. J. Biol. Chem. *285*, 9327–9338. https://doi.org/10.1074/jbc.m109.098251.
8. Guo, C., Yoon, H.S., Franklin, A., Jain, S., Ebert, A., Cheng, H.-L., Hansen, E., Despo, O., Bossen, C., Vettermann, C., et al. (2011).

CTCF-binding elements mediate control of V(D)J recombination. Nature *477*, 424–430. https://doi.org/10.1038/nature10495.

9. Qiu, X., Kumari, G., Gerasimova, T., Du, H., Labaran, L., Singh, A., De, S., Wood, W.H., III, Becker, K.G., Zhou, W., et al. (2018). Sequential Enhancer Sequestration Dysregulates Recombination Center Formation at the IgH Locus. Mol. Cell *70*, 21–33.e6. https://doi.org/10.1016/j.molcel.2018.02.020.
10. Garrett, F.E., Emelyanov, A.V., Sepulveda, M.A., Flanagan, P., Volpi, S., Li, F., Loukinov, D., Eckhardt, L.A., Lobanenkov, V.V., and Birshtein, B.K. (2005). Chromatin Architecture near a Potential 3′ End of the Igh Locus Involves Modular Regulation of Histone Modifications during B-Cell Development and In Vivo Occupancy at CTCF Sites. Mol. Cell Biol. *25*, 1511–1525. https://doi.org/10.1128/mcb.25.4.1511-1525.2005.
11. Volpi, S.A., Verma-Gaur, J., Hassan, R., Ju, Z., Roa, S., Chatterjee, S., Werling, U., Hou, H., Will, B., Steidl, U., et al. (2012). Germline deletion of Igh 3’ regulatory region elements hs 5, 6, 7 (hs5-7) affects B cell-specific regulation, rearrangement, and insulation of the Igh locus. J. Immunol. *188*, 2556–2566. https://doi.org/10.4049/jimmunol.1102763.
12. Benner, C., Isoda, T., and Murre, C. (2015). New roles for DNA cytosine modification, eRNA, anchors, and superanchors in developing B cell progenitors. Proc. Natl. Acad. Sci. USA *112*, 12776–12781. https://doi.org/10.1073/pnas.1512995112.
13. Hnisz, D., Shrinivas, K., Young, R.A., Chakraborty, A.K., and Sharp, P.A. (2017). A Phase Separation Model for Transcriptional Control. Cell *169*, 13–23. https://doi.org/10.1016/j.cell.2017.02.007.
14. Bickmore, W.A., and van Steensel, B. (2013). Genome Architecture: Domain Organization of Interphase Chromosomes. Cell *152*, 1270–1284. https://doi.org/10.1016/j.cell.2013.02.001.
15. Rowley, M.J., and Corces, V.G. (2018). Organizational principles of 3D genome architecture. Nat. Rev. Genet. *19*, 789–800. https://doi.org/10.1038/s41576-018-0060-8.
16. Bolzer, A., Kreth, G., Solovei, I., Koehler, D., Saracoglu, K., Fauth, C., Müller, S., Eils, R., Cremer, C., Speicher, M.R., et al. (2005). Three-Dimensional Maps of All Chromosomes in Human Male Fibroblast Nuclei and Prometaphase Rosettes. PLoS Biol. *3*, e157. https://doi.org/10.1371/journal.pbio.0030157.
17. Cremer, T., and Cremer, M. (2010). Chromosome territories. Cold Spring Harb. Perspect. Biol. *2*, a003889. https://doi.org/10.1101/cshperspect.a003889.
18. Lieberman-Aiden, E., van Berkum, N.L., Williams, L., Imakaev, M., Ragoczy, T., Telling, A., Amit, I., Lajoie, B.R., Sabo, P.J., Dorschner, M.O., et al. (2009). Comprehensive Mapping of Long-Range Interactions Reveals Folding Principles of the Human Genome. Science *326*, 289–293. https://doi.org/10.1126/science.1181369.
19. Schoenfelder, S., Sexton, T., Chakalova, L., Cope, N.F., Horton, A., Andrews, S., Kurukuti, S., Mitchell, J.A., Umlauf, D., Dimitrova, D.S., et al. (2010). Preferential associations between co-regulated genes reveal a transcriptional interactome in erythroid cells. Nat. Genet. *42*, 53–61. https://doi.org/10.1038/ng.496.
20. Bashkirova, E., and Lomvardas, S. (2019). ScienceDirect Olfactory receptor genes make the case for inter-chromosomal interactions. Curr. Opin. Genet. Dev. *55*, 106–113. https://doi.org/10.1016/j.gde.2019.07.004.
21. Dixon, J.R., Selvaraj, S., Yue, F., Kim, A., Li, Y., Shen, Y., Hu, M., Liu, J.S., and Ren, B. (2012). Topological domains in mammalian genomes identified by analysis of chromatin interactions. Nature *485*, 376–380. https://doi.org/10.1038/nature11082.
22. Nora, E.P., Lajoie, B.R., Schulz, E.G., Giorgetti, L., Okamoto, I., Servant, N., Piolot, T., van Berkum, N.L., Meisig, J., Sedat, J., et al. (2012). Spatial partitioning of the regulatory landscape of the X-inactivation centre. Nature *485*, 381–385. https://doi.org/10.1038/nature11049.
23. Galupa, R., and Heard, E. (2017). Topologically Associating Domains in Chromosome Architecture and Gene Regulatory Landscapes during Development, Disease, and Evolution. Cold Spring Harb. Symp. Quant. Biol. *82*, 267–278. https://doi.org/10.1101/sqb.2017.82.035030.
24. Rao, S.S.P., Huntley, M.H., Durand, N.C., Stamenova, E.K., Bochkov, I.D., Robinson, J.T., Sanborn, A.L., Machol, I., Omer, A.D., Lander, E.S., et al. (2014). A 3D Map of the Human Genome at Kilobase Resolution Reveals Principles of Chromatin Looping. Cell *159*, 1665–1680. https://doi.org/10.1016/j.cell.2014.11.021.
25. Zhan, Y., Mariani, L., Barozzi, I., Schulz, E.G., Blüthgen, N., Stadler, M., Tiana, G., and Giorgetti, L. (2017). Reciprocal insulation analysis of Hi-C data shows that TADs represent a functionally but not structurally privileged scale in the hierarchical folding of chromosomes. Genome Res. *27*, 479–490. https://doi.org/10.1101/gr.212803.116.
26. Lin, Y.C., Benner, C., Mansson, R., Heinz, S., Miyazaki, K., Miyazaki, M., Chandra, V., Bossen, C., Glass, C.K., and Murre, C. (2012). Global changes in the nuclear positioning of genes and intra- and interdomain genomic interactions that orchestrate B cell fate. Nat. Immunol. *13*, 1196–1204. https://doi.org/10.1038/ni.2432.
27. Zhang, Y., McCord, R.P., Ho, Y.-J., Lajoie, B.R., Hildebrand, D.G., Simon, A.C., Becker, M.S., Alt, F.W., and Dekker, J. (2012). Spatial Organization of the Mouse Genome and Its Role in Recurrent Chromosomal Translocations. Cell *148*, 908–921. https://doi.org/10.1016/j.cell.2012.02.002.
28. Morshead, K.B., Ciccone, D.N., Taverna, S.D., Allis, C.D., and Oettinger, M.A. (2003). Antigen receptor loci poised for V(D)J rearrangement are broadly associated with BRG1 and flanked by peaks of histone H3 dimethylated at lysine 4. Proc. Natl. Acad. Sci. USA *100*, 11577–11582. https://doi.org/10.1073/pnas.1932643100.
29. Chakraborty, T., Perlot, T., Subrahmanyam, R., Jani, A., Goff, P.H., Zhang, Y., Ivanova, I., Alt, F.W., and Sen, R. (2009). A 220-nucleotide deletion of the intronic enhancer reveals an epigenetic hierarchy in immunoglobulin heavy chain locus activation. J. Exp. Med. *206*, 1019–1027. https://doi.org/10.1084/jem.20081621.
30. Johnson, K., Angelin-Duclos, C., Park, S., and Calame, K.L. (2003). Changes in histone acetylation are associated with differences in accessibility of V(H) gene segments to V-DJ recombination during B-cell ontogeny and development. Mol. Cell Biol. *23*, 2438–2450. https://doi.org/10.1128/mcb.23.7.2438-2450.2003.
31. Chakraborty, T., Chowdhury, D., Keyes, A., Jani, A., Subrahmanyam, R., Ivanova, I., and Sen, R. (2007). Repeat organization and epigenetic regulation of the DH-Cmu domain of the immunoglobulin heavy-chain gene locus. Mol. Cell *27*, 842–850. https://doi.org/10.1016/j.molcel.2007.07.010.
32. Kosak, S.T., Skok, J.A., Medina, K.L., Riblet, R., Beau, M.M.L., Fisher, A.G., and Singh, H. (2002). Subnuclear compartmentalization of immunoglobulin loci during lymphocyte development. Science *296*, 158–162. https://doi.org/10.1126/science.1068768.
33. Rother, M.B., Palstra, R.-J., Jhunjhunwala, S., van Kester, K.A.M., van IJcken, W.F.J., Hendriks, R.W., van Dongen, J.J.M., Murre, C., and van Zelm, M.C. (2016). Nuclear positioning rather than contraction controls ordered rearrangements of immunoglobulin loci. Nucleic Acids Res. *44*, 175–186. https://doi.org/10.1093/nar/gkv928.
34. Johnson, K., Pflugh, D.L., Yu, D., Hesslein, D.G.T., Lin, K.-I., Bothwell, A.L.M., Thomas-Tikhonenko, A., Schatz, D.G., and Calame, K. (2004). B cell–specific loss of histone 3 lysine 9 methylation in the VH locus depends on Pax5. Nat. Immunol. *5*, 853–861. https://doi.org/10.1038/ni1099.
35. Bertolino, E., Reddy, K., Medina, K.L., Parganas, E., Ihle, J., and Singh, H. (2005). Regulation of interleukin 7-dependent immunoglobulin heavy-chain variable gene rearrangements by transcription factor STAT5. Nat. Immunol. *6*, 836–843. https://doi.org/10.1038/ni1226.
36. Fuxa, M., Skok, J., Souabni, A., Salvagiotto, G., Roldan, E., and Busslinger, M. (2004). Pax5 induces V-to-DJ rearrangements and locus contraction of the immunoglobulin heavy-chain gene. Genes Dev. *18*, 411–422. https://doi.org/10.1101/gad.291504.

37. Sayegh, C.E., Sayegh, C., Jhunjhunwala, S., Riblet, R., and Murre, C. (2005). Visualization of looping involving the immunoglobulin heavy-chain locus in developing B cells. Genes Dev. *19*, 322–327. https://doi.org/10.1101/gad.1254305.

38. Lucas, J.S., Zhang, Y., Dudko, O.K., and Murre, C. (2014). 3D Trajectories Adopted by Coding and Regulatory DNA Elements: First-Passage Times for Genomic Interactions. Cell *158*, 339–352. https://doi.org/10.1016/j.cell.2014.05.036.

39. Jhunjhunwala, S., van Zelm, M.C., Peak, M.M., and Murre, C. (2009). Chromatin architecture and the generation of antigen receptor diversity. Cell *138*, 435–448. https://doi.org/10.1016/j.cell.2009.07.016.

40. Medvedovic, J., Ebert, A., Tagoh, H., Tamir, I.M., Schwickert, T.A., Novatchkova, M., Sun, Q., Huis In 't Veld, P.J., Guo, C., Yoon, H.S., et al. (2013). Flexible Long-Range Loops in the VH Gene Region of the Igh Locus Facilitate the Generation of a Diverse Antibody Repertoire. Immunity *39*, 229–244. https://doi.org/10.1016/j.immuni.2013.08.011.

41. Vian, L., Pekowska, A., Rao, S.S.P., Kieffer-Kwon, K.-R., Jung, S., Baranello, L., Huang, S.-C., Khattabi, L.E., Dose, M., Pruett, N., et al. (2018). The Energetics and Physiological Impact of Cohesin Extrusion. Cell *173*, 1165–1170.e20. https://doi.org/10.1016/j.cell.2018.03.072.

42. Gerasimova, T., Guo, C., Ghosh, A., Qiu, X., Montefiori, L., Verma-Gaur, J., Choi, N.M., Feeney, A.J., and Sen, R. (2015). A structural hierarchy mediated by multiple nuclear factors establishes IgH locus conformation. Genes Dev. *29*, 1683–1695. https://doi.org/10.1101/gad.263871.115.

43. Montefiori, L., Wuerffel, R., Roqueiro, D., Lajoie, B., Guo, C., Gerasimova, T., De, S., Wood, W., Becker, K.G., Dekker, J., et al. (2016). Extremely Long-Range Chromatin Loops Link Topological Domains to Facilitate a Diverse Antibody Repertoire. Cell Rep. *14*, 896–906. https://doi.org/10.1016/j.celrep.2015.12.083.

44. Hesslein, D.G.T., Pflugh, D.L., Chowdhury, D., Bothwell, A.L.M., Sen, R., and Schatz, D.G. (2003). Pax5 is required for recombination of transcribed, acetylated, 5' IgH V gene segments. Genes Dev. *17*, 37–42. https://doi.org/10.1101/gad.1031403.

45. Liu, H., Schmidt-Supprian, M., Shi, Y., Hobeika, E., Barteneva, N., Jumaa, H., Pelanda, R., Reth, M., Skok, J., and Rajewsky, K. (2007). Yin Yang 1 is a critical regulator of B-cell development. Genes Dev. *21*, 1179–1189. https://doi.org/10.1101/gad.1529307.

46. Reynaud, D., Demarco, I.A., Reddy, K.L., Schjerven, H., Bertolino, E., Chen, Z., Smale, S.T., Winandy, S., and Singh, H. (2008). Regulation of B cell fate commitment and immunoglobulin heavy-chain gene rearrangements by Ikaros. Nat. Immunol. *9*, 927–936. https://doi.org/10.1038/ni.1626.

47. Qiu, X., Ma, F., Zhao, M., Cao, Y., Shipp, L., Liu, A., Dutta, A., Singh, A., Braikia, F.-Z., De, S., et al. (2020). Altered 3D chromatin structure permits inversional recombination at the IgH locus. Sci. Adv. *6*, eaaz8850. https://doi.org/10.1126/sciadv.aaz8850.

48. Hill, L., Ebert, A., Jaritz, M., Wutz, G., Nagasaka, K., Tagoh, H., Kostanova-Poliakova, D., Schindler, K., Sun, Q., Bönelt, P., et al. (2020). Wapl repression by Pax5 promotes V gene recombination by Igh loop extrusion. Nature *584*, 142–147. https://doi.org/10.1038/s41586-020-2454-y.

49. Rogers, C.H., Mielczarek, O., and Corcoran, A.E. (2021). Dynamic 3D Locus Organization and Its Drivers Underpin Immunoglobulin Recombination. Front. Immunol. *11*, 633705. https://doi.org/10.3389/fimmu.2020.633705.

50. Hu, J., Zhang, Y., Zhao, L., Frock, R.L., Du, Z., Meyers, R.M., Meng, F.-L., Schatz, D.G., and Alt, F.W. (2015). Chromosomal Loop Domains Direct the Recombination of Antigen Receptor Genes. Cell *163*, 947–959. https://doi.org/10.1016/j.cell.2015.10.016.

51. Ba, Z., Lou, J., Ye, A.Y., Dai, H.-Q., Dring, E.W., Lin, S.G., Jain, S., Kyritsis, N., Kieffer-Kwon, K.-R., Casellas, R., et al. (2020). CTCF orchestrates long-range cohesin-driven V(D)J recombinational scanning. Nature *586*, 305–310. https://doi.org/10.1038/s41586-020-2578-0.

52. Ciccone, D.N., Namiki, Y., Chen, C., Morshead, K.B., Wood, A.L., Johnston, C.M., Morris, J.W., Wang, Y., Sadreyev, R., Corcoran, A.E., et al. (2019). The murine IgH locus contains a distinct DNA sequence motif for the chromatin regulatory factor CTCF. J. Biol. Chem. *294*, 13580–13592. https://doi.org/10.1074/jbc.ra118.007348.

53. Loguercio, S., Barajas-Mora, E.M., Shih, H.-Y., Krangel, M.S., and Feeney, A.J. (2018). Variable Extent of Lineage-Specificity and Developmental Stage-Specificity of Cohesin and CCCTC-Binding Factor Binding Within the Immunoglobulin and Cell Receptor Loci. Front. Immunol. *9*, 2802–2819. https://doi.org/10.3389/fimmu.2018.00425.

54. Dariavach, P., Williams, G.T., Campbell, K., Pettersson, S., and Neuberger, M.S. (1991). The mouse IgH 3'-enhancer. Eur. J. Immunol. *21*, 1499–1504. https://doi.org/10.1002/eji.1830210625.

55. Pinaud, E., Khamlichi, A.A., Morvan, C.L., Drouet, M., Nalesso, V., Bert, M.L., and Cogné, M. (2001). Localization of the 3′ IgH Locus Elements that Effect Long-Distance Regulation of Class Switch Recombination. Immunity *15*, 187–199. https://doi.org/10.1016/s1074-7613(01)00181-9.

56. Perlot, T., Alt, F.W., Bassing, C.H., Suh, H., and Pinaud, E. (2005). Elucidation of IgH intronic enhancer functions via germ-line deletion. Proc. Natl. Acad. Sci. USA *102*, 14362–14367. https://doi.org/10.1073/pnas.0507090102.

57. Afshar, R., Pierce, S., Bolland, D.J., Corcoran, A., and Oltz, E.M. (2006). Regulation of IgH gene assembly: role of the intronic enhancer and 5'DQ52 region in targeting DHJH recombination. J. Immunol. *176*, 2439–2447.

58. Bolland, D.J., Wood, A.L., Afshar, R., Featherstone, K., Oltz, E.M., and Corcoran, A.E. (2007). Antisense intergenic transcription precedes Igh D-to-J recombination and is controlled by the intronic enhancer Emu. Mol. Cell Biol. *27*, 5523–5533. https://doi.org/10.1128/mcb.02407-06.

59. Bolland, D.J., Wood, A.L., Johnston, C.M., Bunting, S.F., Morgan, G., Chakalova, L., Fraser, P.J., and Corcoran, A.E. (2004). Antisense intergenic transcription in V(D)J recombination. Nat. Immunol. *5*, 630–637. https://doi.org/10.1038/ni1068.

60. Guo, C., Gerasimova, T., Hao, H., Ivanova, I., Chakraborty, T., Selimyan, R., Oltz, E.M., and Sen, R. (2011). Two Forms of Loops Generate the Chromatin Conformation of the Immunoglobulin Heavy-Chain Gene Locus. Cell *147*, 332–343. https://doi.org/10.1016/j.cell.2011.08.049.

61. Born, W., White, J., Kappler, J., and Marrack, P. (1988). Rearrangement of IgH genes in normal thymocyte development. J. Immunol. *140*, 3228–3232.

62. Kumari, G., Gerasimova, T., Du, H., De, S., Wood, W.H., III, Becker, K.G., and Sen, R. (2018). Misregulation of the IgH Locus in Thymocytes. Front. Immunol. *9*, 565–612. https://doi.org/10.3389/fimmu.2018.02426.

63. Giorgetti, L., Galupa, R., Nora, E.P., Piolot, T., Lam, F., Dekker, J., Tiana, G., and Heard, E. (2014). Predictive polymer modeling reveals coupled fluctuations in chromosome conformation and transcription. Cell *157*, 950–963. https://doi.org/10.1016/j.cell.2014.03.025.

64. Zenk, F., Zhan, Y., Kos, P., Löser, E., Atinbayeva, N., Schächtle, M., Tiana, G., Giorgetti, L., and Iovino, N. (2021). HP1 drives de novo 3D genome reorganization in early Drosophila embryos. Nature *593*, 289–293. https://doi.org/10.1038/s41586-021-03460-z.

65. Barbieri, M., Chotalia, M., Fraser, J., Lavitas, L.-M., Dostie, J., Pombo, A., and Nicodemi, M. (2012). Complexity of chromatin folding is captured by the strings and binders switch model. Proc. Natl. Acad. Sci. USA *109*, 16173–16178. https://doi.org/10.1073/pnas.1204799109.

66. Lion, M., Muhire, B., Namiki, Y., Tolstorukov, M.Y., and Oettinger, M.A. (2020). Alterations in chromatin at antigen receptor loci define lineage progression during B lymphopoiesis. Proc. Natl. Acad. Sci. USA *127*, 201914923. https://doi.org/10.1073/pnas.1914923117.

67. Ji, Y., Resch, W., Corbett, E., Yamane, A., Casellas, R., and Schatz, D.G. (2010). The in vivo pattern of binding of RAG1 and RAG2 to antigen receptor loci. Cell *141*, 419–431. https://doi.org/10.1016/j.cell.2010.03.010.

68. Dryden, N.H., Broome, L.R., Dudbridge, F., Johnson, N., Orr, N., Schoenfelder, S., Nagano, T., Andrews, S., Wingett, S., Kozarewa, I., et al. (2014). Unbiased analysis of potential targets of breast cancer susceptibility loci by Capture Hi-C. Genome Res. *24*, 1854–1868. https://doi.org/10.1101/gr.175034.114.

69. Schoenfelder, S., Sugar, R., Dimond, A., Javierre, B.-M., Armstrong, H., Mifsud, B., Dimitrova, E., Matheson, L., Tavares-Cadete, F., Furlan-Magaril, M., et al. (2015). Polycomb repressive complex PRC1 spatially constrains the mouse embryonic stem cell genome. Nat. Genet. *47*, 1179–1186. https://doi.org/10.1038/ng.3393.

70. Quinodoz, S.A., Ollikainen, N., Tabak, B., Palla, A., Schmidt, J.M., Detmar, E., Lai, M.M., Shishkin, A.A., Bhat, P., Takei, Y., et al. (2018). Higher-Order Inter-chromosomal Hubs Shape 3D Genome Organization in the Nucleus. Cell *174*, 744–757.e24. https://doi.org/10.1016/j.cell.2018.05.024.

71. Maass, P.G., Barutcu, A.R., Weiner, C.L., and Rinn, J.L. (2018). Interchromosomal Contact Properties in Live-Cell Imaging and in Hi-C. Mol. Cell *69*, 1039–1045.e3. https://doi.org/10.1016/j.molcel.2018.02.007.

72. Monahan, K., Horta, A., and Lomvardas, S. (2018). LHX2- and LDB1-mediated trans interactions regulate olfactory receptor choice. Nature *565*, 448–453. https://doi.org/10.1038/s41586-018-0845-0.

73. Bortnick, A., He, Z., Aubrey, M., Chandra, V., Denholtz, M., Chen, K., Lin, Y.C., and Murre, C. (2020). Plasma Cell Fate Is Orchestrated by Elaborate Changes in Genome Compartmentalization and Inter-chromosomal Hubs. Cell Rep. *31*, 107470. https://doi.org/10.1016/j.celrep.2020.03.034.

74. Tan, L., Xing, D., Daley, N., and Xie, X.S. (2019). Three-dimensional genome structures of single sensory neurons in mouse visual and olfactory systems. Nat. Struct. Mol. Biol. *26*, 297–307. https://doi.org/10.1038/s41594-019-0205-2.

75. Payne, A.C., Chiang, Z.D., Reginato, P.L., Mangiameli, S.M., Murray, E.M., Yao, C.-C., Markoulaki, S., Earl, A.S., Labade, A.S., Jaenisch, R., et al. (2020). In situ genome sequencing resolves DNA sequence and structure in intact biological samples. Science *371*, eaay3446. https://doi.org/10.1126/science.aay3446.

76. Johanson, T.M., Coughlan, H.D., Lun, A.T.L., Bediaga, N.G., Naselli, G., Garnham, A.L., Harrison, L.C., Smyth, G.K., and Allan, R.S. (2018). Genome-wide analysis reveals no evidence of trans chromosomal regulation of mammalian immune development. PLoS Genet. *14*, e1007431. https://doi.org/10.1371/journal.pgen.1007431.

77. Lorsbach, R.B., Moore, J., Ang, S.O., Sun, W., Lenny, N., and Downing, J.R. (2004). Role of RUNX1 in adult hematopoiesis: analysis of RUNX1-IRES-GFP knock-in mice reveals differential lineage expression. Blood *103*, 2522–2529. https://doi.org/10.1182/blood-2003-07-2439.

78. Deau, M.-C., Heurtier, L., Frange, P., Suarez, F., Bole-Feysot, C., Nitschke, P., Cavazzana, M., Picard, C., Durandy, A., Fischer, A., et al. (2014). A human immunodeficiency caused by mutations in the PIK3R1 gene. J. Clin. Invest. *124*, 3923–3928. https://doi.org/10.1172/jci75746.

79. Juntilla, M.M., Wofford, J.A., Birnbaum, M.J., Rathmell, J.C., and Koretzky, G.A. (2007). Akt1 and Akt2 are required for $\alpha\beta$ thymocyte survival and differentiation. Proc. Natl. Acad. Sci. USA *104*, 12105–12110. https://doi.org/10.1073/pnas.0705285104.

80. Muto, A., Hoshino, H., Madisen, L., Yanai, N., Obinata, M., Karasuyama, H., Hayashi, N., Nakauchi, H., Yamamoto, M., Groudine, M., et al. (1998). Identification of Bach2 as a B-cell-specific partner for small maf proteins that negatively regulate the immunoglobulin heavy chain gene 3' enhancer. EMBO J. *17*, 5734–5743. https://doi.org/10.1093/emboj/17.19.5734.

81. Dengler, H.S., Baracho, G.V., Omori, S.A., Bruckner, S., Arden, K.C., Castrillon, D.H., Depinho, R.A., and Rickert, R.C. (2008). Distinct functions for the transcription factor Foxo1 at various stages of B cell differentiation. Nat. Immunol. *9*, 1388–1398. https://doi.org/10.1038/ni.1667.

82. Kerdiles, Y.M., Beisner, D.R., Tinoco, R., Dejean, A.S., Castrillon, D.H., DePinho, R.A., and Hedrick, S.M. (2009). Foxo1 links homing and survival of naive T cells by regulating L-selectin, CCR7 and interleukin 7 receptor. Nat. Immunol. *10*, 176–184. https://doi.org/10.1038/ni.1689.

83. Kolovos, P., Georgomanolis, T., Koeferle, A., Larkin, J.D., Brant, L., Nikolicć, M., Gusmao, E.G., Zirkel, A., Knoch, T.A., van Ijcken, W.F., et al. (2016). Binding of nuclear factor κB to noncanonical consensus sites reveals its multimodal role during the early inflammatory response. Genome Res. *26*, 1478–1489. https://doi.org/10.1101/gr.210005.116.

84. Oudelaar, A.M., Beagrie, R.A., Gosden, M., de Ornellas, S., Georgiades, E., Kerry, J., Hidalgo, D., Carrelha, J., Shivalingam, A., El-Sagheer, A.H., et al. (2020). Dynamics of the 4D genome during in vivo lineage specification and differentiation. Nat. Commun. *11*, 2722. https://doi.org/10.1038/s41467-020-16598-7.

85. Sima, J., Chakraborty, A., Dileep, V., Michalski, M., Klein, K.N., Holcomb, N.P., Turner, J.L., Paulsen, M.T., Rivera-Mulia, J.C., Trevilla-Garcia, C., et al. (2019). Identifying cis Elements for Spatiotemporal Control of Mammalian DNA Replication. Cell *176*, 816–830.e18. https://doi.org/10.1016/j.cell.2018.11.036.

86. Stubbington, M.J.T., and Corcoran, A.E. (2013). Non-coding transcription and large-scale nuclear organisation of immunoglobulin recombination. Curr. Opin. Genet. Dev. *23*, 81–88. https://doi.org/10.1016/j.gde.2013.01.001.

87. Degner, S.C., Verma-Gaur, J., Wong, T.P., Bossen, C., Iverson, G.M., Torkamani, A., Vettermann, C., Lin, Y.C., Ju, Z., Schulz, D., et al. (2011). CCCTC-binding factor (CTCF) and cohesin influence the genomic architecture of the Igh locus and antisense transcription in pro-B cells. Proc. Natl. Acad. Sci. USA *108*, 9566–9571. https://doi.org/10.1073/pnas.1019391108.

88. Dai, H.-Q., Hu, H., Lou, J., Ye, A.Y., Ba, Z., Zhang, X., Zhang, Y., Zhao, L., Yoon, H.S., Chapdelaine-Williams, A.M., et al. (2021). Loop extrusion mediates physiological Igh locus contraction for RAG scanning. Nature *573*, 600–606. https://doi.org/10.1038/s41586-020-03121-7.

89. Guo, Y., Xu, Q., Canzio, D., Shou, J., Li, J., Gorkin, D.U., Jung, I., Wu, H., Zhai, Y., Tang, Y., et al. (2015). CRISPR Inversion of CTCF Sites Alters Genome Topology and Enhancer/Promoter Function. Cell *162*, 900–910. https://doi.org/10.1016/j.cell.2015.07.038.

90. Crippa, M., Zhan, Y., and Tiana, G. (2020). Effective model of loop extrusion predicts chromosomal domains. Phys. Rev. E *102*, 032414. https://doi.org/10.1103/physreve.102.032414.

91. Mach, P., Kos, P.I., Zhan, Y., Cramard, J., Gaudin, S., Tünnermann, J., Marchi, E., Eglinger, J., Zuin, J., Kryzhanovska, M., et al. (2022). Cohesin and CTCF control the dynamics of chromosome folding. Nat. Genet. *54*, 1907–1918. https://doi.org/10.1038/s41588-022-01232-7.

92. Lin, S.G., Ba, Z., Alt, F.W., and Zhang, Y. (2018). RAG Chromatin Scanning During V(D)J Recombination and Chromatin Loop Extrusion are Related Processes. Adv. Immunol. *139*, 93–135. https://doi.org/10.1016/bs.ai.2018.07.001.

93. Gabriele, M., Brandão, H.B., Grosse-Holz, S., Jha, A., Dailey, G.M., Cattoglio, C., Hsieh, T.-H.S., Mirny, L., Zechner, C., and Hansen, A.S. (2022). Dynamics of CTCF- and cohesin-mediated chromatin looping revealed by live-cell imaging. Science *376*, 496–501. https://doi.org/10.1126/science.abn6583.

94. Khanna, N., Zhang, Y., Lucas, J.S., Dudko, O.K., and Murre, C. (2019). Chromosome dynamics near the sol-gel phase transition dictate the timing of remote genomic interactions. Nat. Commun. *10*, 2771. https://doi.org/10.1038/s41467-019-10628-9.

95. Canzio, D., Nwakeze, C.L., Horta, A., Rajkumar, S.M., Coffey, E.L., Duffy, E.E., Duffié, R., Monahan, K., O'Keeffe, S., Simon, M.D., et al. (2019). Antisense lncRNA Transcription Mediates DNA Demethylation to Drive Stochastic Protocadherin α Promoter Choice. Cell *177*, 639–653.e15. https://doi.org/10.1016/j.cell.2019.03.008.

96. Hewitt, S.L., Chaumeil, J., and Skok, J.A. (2010). Chromosome dynamics and the regulation of V(D)J recombination. Immunol. Rev. *237*, 43–54. https://doi.org/10.1111/j.1600-065x.2010.00931.x.

97. Bossen, C., Mansson, R., and Murre, C. (2012). Chromatin topology and the regulation of antigen receptor assembly. Annu. Rev. Immunol. *30*, 337–356. https://doi.org/10.1146/annurev-immunol-020711-075003.

98. Nagano, T., Lubling, Y., Stevens, T.J., Schoenfelder, S., Yaffe, E., Dean, W., Laue, E.D., Tanay, A., and Fraser, P. (2013). Single-cell Hi-C reveals cell -to-cell variability in chromosome structure. Nature *502*, 59–64. https://doi.org/10.1038/nature12593.

99. Nagano, T., Lubling, Y., Varnái, C., Dudley, C., Leung, W., Babran, Y., Mendelson Cohen, N., Wingett, S., Fraser, P., and Tanay, A. (2017). Cell-cycle dynamics of chromosomal organization at single-cell resolution. Nature *547*, 61–67. https://doi.org/10.1038/nature23001.

100. Roldan, E., Fuxa, M., Chong, W., Martinez, D., Novatchkova, M., Busslinger, M., and Skok, J.A. (2005). Locus "decontraction" and centromeric recruitment contribute to allelic exclusion of the immunoglobulin heavy-chain gene. Nat. Immunol. *6*, 31–41. https://doi.org/10.1038/ni1150.

101. Gopalakrishnan, S., Majumder, K., Predeus, A., Huang, Y., Koues, O.I., Verma-Gaur, J., Loguercio, S., Su, A.I., Feeney, A.J., Artyomov, M.N., et al. (2013). Unifying model for molecular determinants of the preselection Vβ repertoire. Proc. Natl. Acad. Sci. USA *110*, E3206–E3215. https://doi.org/10.1073/pnas.1304048110.

102. Park, S.-K., Xiang, Y., Feng, X., and Garrard, W.T. (2014). Pronounced cohabitation of active immunoglobulin genes from three different chromosomes in transcription factories during maximal antibody synthesis. Genes Dev. *28*, 1159–1164. https://doi.org/10.1101/gad.237479.114.

103. Osborne, C.S., Chakalova, L., Mitchell, J.A., Horton, A., Wood, A.L., Bolland, D.J., Corcoran, A.E., and Fraser, P. (2007). Myc dynamically and preferentially relocates to a transcription factory occupied by Igh. PLoS Biol. *5*, e192. https://doi.org/10.1371/journal.pbio.0050192.

104. Hakim, O., Resch, W., Yamane, A., Klein, I., Kieffer-Kwon, K.-R., Jankovic, M., Oliveira, T., Bothmer, A., Voss, T.C., Ansarah-Sobrinho, C., et al. (2012). DNA damage defines sites of recurrent chromosomal translocations in B lymphocytes. Nature *484*, 69–74. https://doi.org/10.1038/nature10909.

105. Rocha, P.P., Micsinai, M., Kim, J.R., Hewitt, S.L., Souza, P.P., Trimarchi, T., Strino, F., Parisi, F., Kluger, Y., and Skok, J.A. (2012). Close Proximity to Igh Is a Contributing Factor to AID-Mediated Translocations. Mol. Cell *47*, 873–885. https://doi.org/10.1016/j.molcel.2012.06.036.

106. Somasundaram, R., Prasad, M.A.J., Ungerbäck, J., and Sigvardsson, M. (2015). Transcription factor networks in B-cell differentiation link development to acute lymphoid leukemia. Blood *126*, 144–152. https://doi.org/10.1182/blood-2014-12-575688.

107. Lin, Y.C., Jhunjhunwala, S., Benner, C., Heinz, S., Welinder, E., Mansson, R., Sigvardsson, M., Hagman, J., Espinoza, C.A., Dutkowski, J., et al. (2010). A global network of transcription factors, involving E2A, EBF1 and Foxo1, that orchestrates B cell fate. Nat. Immunol. *11*, 635–643. https://doi.org/10.1038/ni.1891.

108. Mansson, R., Welinder, E., Ahsberg, J., Lin, Y.C., Benner, C., Glass, C.K., Lucas, J.S., Sigvardsson, M., and Murre, C. (2012). Positive intergenic feedback circuitry, involving EBF1 and FOXO1, orchestrates B-cell fate. Proc. Natl. Acad. Sci. USA *109*, 21028–21033. https://doi.org/10.1073/pnas.1211427109.

109. Maier, H., Ostraat, R., Gao, H., Fields, S., Shinton, S.A., Medina, K.L., Ikawa, T., Murre, C., Singh, H., Hardy, R.R., et al. (2004). Early B cell factor cooperates with Runx1 and mediates epigenetic changes associated with mb-1 transcription. Nat. Immunol. *5*, 1069–1077. https://doi.org/10.1038/ni1119.

110. Somasundaram, R., Jensen, C.T., Tingvall-Gustafsson, J., Åhsberg, J., Okuyama, K., Prasad, M., Hagman, J.R., Wang, X., Soneji, S., Strid, T., et al. (2021). EBF1 and PAX5 control pro-B cell expansion via opposing regulation of the Myc gene. Blood *137*, 3037–3049. https://doi.org/10.1182/blood.2020009564.

111. Nutt, S.L., and Kee, B.L. (2007). The transcriptional regulation of B cell lineage commitment. Immunity *26*, 715–725. https://doi.org/10.1016/j.immuni.2007.05.010.

112. Choi, N.M., Loguercio, S., Verma-Gaur, J., Degner, S.C., Torkamani, A., Su, A.I., Oltz, E.M., Artyomov, M., and Feeney, A.J. (2013). Deep Sequencing of the Murine Igh Repertoire Reveals Complex Regulation of Nonrandom V Gene Rearrangement Frequencies. J. Immunol. https://doi.org/10.4049/jimmunol.1301279.

113. Spanopoulou, E., Roman, C.A., Corcoran, L.M., Schlissel, M.S., Silver, D.P., Nemazee, D., Nussenzweig, M.C., Shinton, S.A., Hardy, R.R., and Baltimore, D. (1994). Functional immunoglobulin transgenes guide ordered B-cell differentiation in Rag-1-deficient mice. Genes Dev. *8*, 1030–1042.

114. Mombaerts, P., Iacomini, J., Johnson, R.S., Herrup, K., Tonegawa, S., and Papaioannou, V.E. (2003). RAG-1-deficient mice have no mature B and T lymphocytes. Cell *68*, 869–877.

115. Martin, F., Chen, X., and Kearney, J.F. (1997). Development of VH81X transgene-bearing B cells in fetus and adult: sites for expansion and deletion in conventional and CD5/B1 cells. Int. Immunol. *9*, 493–505.

116. Kim, D., Langmead, B., and Salzberg, S.L. (2015). HISAT: a fast spliced aligner with low memory requirements. Nat. Methods *12*, 357–360. https://doi.org/10.1038/nmeth.3317.

117. Eden, E., Navon, R., Steinfeld, I., Lipson, D., and Yakhini, Z. (2009). GOrilla: a tool for discovery and visualization of enriched GO terms in ranked gene lists. BMC Bioinf. *10*, 48. https://doi.org/10.1186/1471-2105-10-48.

118. Shannon, P., Markiel, A., Ozier, O., Baliga, N.S., Wang, J.T., Ramage, D., Amin, N., Schwikowski, B., and Ideker, T. (2003). Cytoscape: A Software Environment for Integrated Models of Biomolecular Interaction Networks. Genome Res. *13*, 2498–2504. https://doi.org/10.1101/gr.1239303.

119. Langmead, B., and Salzberg, S.L. (2012). Fast gapped-read alignment with Bowtie 2. Nat. Methods *9*, 357–359. https://doi.org/10.1038/nmeth.1923.

120. Nagano, T., Várnai, C., Schoenfelder, S., Javierre, B.-M., Wingett, S.W., and Fraser, P. (2015). Comparison of Hi-C results using in-solution versus in-nucleus ligation. Genome Biol. *16*, 175. https://doi.org/10.1186/s13059-015-0753-7.

121. Bolland, D.J., King, M.R., Reik, W., Corcoran, A.E., and Krueger, C. (2013). Robust 3D DNA FISH Using Directly Labeled Probes. J. Vis. Exp. https://doi.org/10.3791/50587.

122. Wingett, S., Ewels, P., Furlan-Magaril, M., Nagano, T., Schoenfelder, S., Fraser, P., and Andrews, S. (2015). HiCUP: pipeline for mapping and processing Hi-C data. F1000Res. *4*, 1310. https://doi.org/10.12688/f1000research.7334.1.

123. Belton, J.-M., McCord, R.P., Gibcus, J.H., Naumova, N., Zhan, Y., and Dekker, J. (2012). Hi-C: a comprehensive technique to capture the conformation of genomes. Methods *58*, 268–276. https://doi.org/10.1016/j.ymeth.2012.05.001.

124. Imakaev, M., Fudenberg, G., McCord, R.P., Naumova, N., Goloborodko, A., Lajoie, B.R., Dekker, J., and Mirny, L.A. (2012). Iterative correction of Hi-C data reveals hallmarks of chromosome organization. Nat. Methods *9*, 999–1003. https://doi.org/10.1038/nmeth.2148.

125. Saldanha, A.J. (2004). Java Treeview—extensible visualization of microarray data. Bioinformatics *20*, 3246–3248. https://doi.org/10.1093/bioinformatics/bth349.

126. ENCODE Project Consortium (2012). An Integrated Encyclopedia of DNA Elements in the Human Genome. Nature *489*, 57–74. https://doi.org/10.1038/nature11247.

127. Lex, A., Gehlenborg, N., Strobelt, H., Vuillemot, R., and Pfister, H. (2014). UpSet: Visualization of Intersecting Sets. IEEE Trans. Vis. Comput. Graph. *20*, 1983–1992. https://doi.org/10.1109/tvcg.2014.2346248.

128. Zhang, Y., Liu, T., Meyer, C.A., Eeckhoute, J., Johnson, D.S., Bernstein, B.E., Nusbaum, C., Myers, R.M., Brown, M., Li, W., et al. (2008). Model-based analysis of ChIP-Seq (MACS). Genome Biol. *9*, R137. https://doi.org/10.1186/gb-2008-9-9-r137.

CellPress
OPEN ACCESS

STAR★METHODS

KEY RESOURCES TABLE

REAGENT or RESOURCE	SOURCE	IDENTIFIER
**Antibodies**		
TER-119 Monoclonal Antibody, Biotin	eBioscience	Clone TER-119; Cat# 13-5921-82; RRID: AB_466797
CD11b Monoclonal Antibody, Biotin	eBioscience	Clone M1/70; Cat# 13-0112-82; RRID:AB_466359
Ly-6G/Gr-1 Monoclonal Antibody, Biotin	eBioscience	Clone RB6-8C5; Cat# 13-5931-82; RRID: AB_466800
RAT ANTI MOUSE Ly-6C:Biotin	AbD Serotec	Clone ER-MP20; Cat# MCA2389B; RRID: AB_844550
CD3e Monoclonal Antibody, Biotin	eBioscience	Clone 145-2C11; Cat# 13-0031-82; RRID: AB_466319
BV421 Rat Anti-Mouse CD45R/B220	Biolegend	Clone RA3-6B2; Cat# 103239; RRID: AB_10933424
PerCP-Cy5.5 Rat Anti-Mouse CD19	BD Biosciences	Clone 1D3; Cat# 551001; RRID: AB_394004
CD43-FITC	BD Biosciences	Clone S7; Cat# 553270; RRID: AB_394747
CD25-PE	BD Biosciences	Clone PC61; Cat# 553866; RRID:AB_395101
CD8-FITC	eBioscience	Clone 53–6.7; Cat# 11-0081-82; RRID:AB_464915
CD4-PercpCy5.5	eBioscience	Clone RM45; Cat# 45-0042-82; RRID:AB_1107001
**Chemicals, peptides, and recombinant proteins**		
Dynabeads MyOne Streptavidin C1 beads	Life Technologies	Cat# 65001
Ampure XP beads	Beckman Coulter	Cat# A63881
**Critical commercial assays**		
Quant-iT PicoGreen dsDNA Assay Kit	Invitrogen	Cat# P7589
NucleoBond BAC 100 kit	Macherey-Nagel	Cat# 740579
QiaQuick PCR purification kit	Qiagen	Cat# 28104
T7 MegaScript kit	Life Technologies	Cat# AM1334
Ambion MEGAclear kit	Life Technologies	Cat# AM1908
KAPA Library Quantification Kit	Roche	Cat# KK4824
Qubit dsDNA BR Assay kit	Life Technologies	Cat# Q32850
**Deposited data**		
Raw and analyzed data	This paper	GEO: GSE208602
H3K4me3 ChIP-seq pro-B	Bolland et al.[4]	GEO: GSE80155
CTCF ChIP-seq pro-B	Choi et al.[112]	GEO: GSE47766
**Experimental models: Organisms/strains**		
Mouse: $Rag1^{tmBal}$	David Baltimore	Spanopoulou et al.[113]
Mouse: $Rag1^{tmMom}$	Peter Mombaerts	Mombaerts et al.[114]
Mouse: V_H81X^{Tg}	John Kearney/Rudi Hendriks	Martin et al.[115]
**Oligonucleotides**		
See Table S6A		N/A
**Recombinant DNA**		
See Table S6B		N/A
**Software and algorithms**		
Seqmonk	The Babraham Institute	https://www.bioinformatics.babraham.ac.uk/projects/seqmonk/
HOMER version 4.7	UCSD	http://homer.ucsd.edu/homer/
HiCUP version 0.7.2	The Babraham Institute	https://www.bioinformatics.babraham.ac.uk/projects/hicup/
HISAT2		Kim et al.[116]
GOrilla		Eden[117]
Cytoscape		Shannon[118]
Trim Galore	The Babraham Institute	https://www.bioinformatics.babraham.ac.uk/projects/trim_galore/

(Continued on next page)

Continued

REAGENT or RESOURCE	SOURCE	IDENTIFIER
Bowtie2 version 2.3.2		Langmead and Salzberg[119]
HiCseg version 1.1	R package	https://cran.r-project.org/web/packages/HiCseg/index.html

RESOURCE AVAILABILITY

Lead contact

Further information and requests for resources and reagents should be directed to and will be fulfilled by the lead contact, Anne Corcoran (anne.corcoran@babraham.ac.uk).

Materials availability

This study did not generate new unique reagents.

Data and code availability

- The Hi-C, Capture Hi-C and nuclear RNAseq raw sequencing files generated in this study, as well as processed files, have been deposited with GEO, accession number GEO: GSE208602. This paper analyses existing, publicly available data. The accession numbers for these datasets are listed in the key resources table.
- This paper does not report original code.
- Any additional information required to reanalyze the data reported in this paper is available from the lead contact upon request.

EXPERIMENTAL MODEL AND STUDY PARTICIPANT DETAILS

Mice

Wild type mice were on a C57BL/6 background. Two Rag knockout strains were used: $Rag^{-/-Mom}$ ($Rag1^{tmMom}$) mice[114] and $Rag^{-/-Bal}$ ($Rag1^{tmBal}$) mice,[113] collectively referred to as $Rag^{-/-}$. Both $Rag^{-/-}$ strains were on a C57BL/6 background. Mice harboring a Vh81X transgene in BALB/c[115] were crossed with $Rag^{-/-Mom}$ mice, yielding Rag/81X mice, with sufficient backcrossing to assume a C57BL/6 background.

Mice were maintained in accordance with Babraham Institute Animal Welfare and Ethical Review Body under Project License 80/2529 and P9B90446F approved by the UK Home Office. Recommended ARRIVE reporting guidelines were followed. Mice were bred and maintained in the Babraham Institute Biological Services Unit under Specific Opportunistic Pathogen Free (SOPF) conditions. After weaning, mice were maintained in individually ventilated cages (2–5 mice per cage). Mice were fed CRM (P) VP diet (Special Diet Services) *ad libitum*, and millet, sunflower or poppy seeds at cage-cleaning as environmental enrichment. Health status was monitored closely and any mouse with clinical signs of ill-health or distress persisting for more than three days was culled. Treatment with antibiotics was not permitted to avoid interference with immune function. Thus, all mice remained 'sub-threshold' under UK Home Office severity categorization. Mice (all mixed sex) were taken at 6–12 weeks old and killed according to Schedule 1 of the Animals (Scientific Procedures) Act 1986.

METHOD DETAILS

Primary cells

Following CO_2 asphyxiation and cervical dislocation, mouse bone marrow was flushed from femurs and tibias, and the cell suspension was collected by centrifugation and resuspended in ice-cold PBS.

For Hi-C library preparation bone marrow cells from $Rag^{-/-}$ and Rag/81X mice were stained with magnetic microbeads conjugated to anti-CD19 antibodies (Miltenyi Biotec) and separated on a large selection (LS) MACS columns (Miltenyi Biotec) according to manufacturer's instructions.

For 3D DNA FISH bone marrow cells were MACS depleted of non-B cells using biotinylated antibodies: TER119 (eBioscience; 1:400), CD11b (eBioscience; 1:1600), GR1 (eBioscience; 1:1600), LY6c (AbD Serotec; 1:400) and CD3e (eBioscience; 1:800) and separated using magnetic beads (Miltenyi Biotec) on a large selection (LS) MACS columns (Miltenyi Biotec) according to manufacturer's instructions. The depleted flow-through cells were stained with B220-BV421 (Biolegend; 1:200), CD19-PercpCy5.5 (BD Pharmingen; 1:400), CD43-FITC (BD Pharmingen; 1:200) and CD25-PE (BD Pharmingen) on ice for 45 min, washed and sorted on a FACSAria machine (Becton Dickinson) as follows: $Rag^{-/-}$ pro-B cells: $B220^+$, $CD19^+$, $CD43^+$; Rag/81X pre-B cells: $B220^+$, $CD19^+$, $CD43^-$, $CD25^+$.

For nuclear RNA-seq bone marrow cells from $Rag^{-/-}$ and Rag/81X mice were isolated as described for 3D DNA FISH, except that for pro-B cells, MACS enrichment with magnetic microbeads conjugated to anti-CD19 antibodies was performed prior to FACS sorting instead of depletion.

Thymi from wild type mice were disrupted with forceps, flushed through a 70 μm cell strainer and cells were collected by centrifugation and resuspended in ice-cold PBS. For Hi-C library preparation cells from four whole thymi were pooled to form one biological replicate. For 3D DNA FISH cells from one thymus were stained with CD8-FITC (eBioscience) and CD4-PercpCy5.5 (eBioscience) on ice for 45 min, washed with PBS and sorted on a FACSAria machine for double-positive ($CD4^+$, $CD8^+$) T cells.

Hi-C

Hi-C with in-nucleus ligation was performed as described previously[24,120] with minor modifications. Two biological replicates for each $Rag^{-/-Mom}$, $Rag^{-/-Bal}$, Rag/81X and thymocytes were generated. 20-50x10^6 cells were cross-linked for 10 min at room temperature (RT) in 37 mL of DMEM supplemented with 10% FBS (Invitrogen) and containing a final concentration of 2% formaldehyde (Agar Scientific). The reaction was quenched with 1M glycine added to a final concentration of 0.125M and incubation for 5 min at RT followed by 15 min incubation on ice. Cells were centrifuged at 1500 rpm at 4°C for 10 min, washed in ice-cold 1x PBS and snap frozen in liquid nitrogen then stored at −80°C.

Fixed cell pellet was thawed and resuspended in lysis buffer (10 mM Tris-HCl pH = 8, 0.2% NP-40, 10 mM NaCl, 1 protease inhibitor cocktail tablet (Roche complete, EDTA free), H2O) and incubated on ice for 30 min with occasional mixing. Cells were centrifuged and the cell pellet was resuspended in 1 mL of ice-cold 1.25X NEB2 buffer. Samples were split into four 250 μL aliquots and a further 108 μL of ice-cold 1.25x NEB2 buffer was added to each aliquot. To remove proteins not directly cross-linked to DNA, 11 μL of 10% SDS was added to each aliquot and they were incubated at 37°C for 1 h with shaking at 950 rpm. To quench the SDS, 75 μL of 10% Triton X-100 was added and incubated for a further 1 h at 37°C.

The cross-linked chromatin was digested with 1500U of HindIII (New England Biolabs) and incubated overnight at 37°C with shaking at 950 rpm.

Restriction fragment ends in cross-linked digested chromatin were filled-in and biotinylated by the addition of 6 μL 10x NEB Buffer 2, 2 μL nuclease-free water, 37.5 μL of 0.4 mM biotin-14-dATP (Life Technologies), 1.5 μL each of 10 mM dCTP, dGTP, dTTP and 50U of Klenow DNA polymerase I large fragment (New England Biolabs). Reactions were incubated at 37°C for 75 min. Samples were mixed by shaking at 700 rpm for 5 s every 30 s.

541 μL of ligation reaction mix (100 μL of 10x NEB ligation buffer, 10 μL of 100x BSA, 25U of T4 DNA ligase (Invitrogen) and 429 μL of H_2O) was added to biotinylated samples and incubated at 16°C for 4 h, then at RT for 30 min.

Cross-links were reversed by adding 600 μg of Proteinase K (Qiagen) to each sample and incubated overnight at 65°C. 100 μg of RNaseA (Qiagen) was added to each sample and incubated at 37°C for 1 h. All steps up to this point were carried out in undisrupted nuclei.

DNA was purified by a phenol (Sigma-Aldrich) extraction followed by two phenol-chloroform (Sigma-Aldrich) extractions and precipitated with 0.1 volume of 3M NaOAc and 2.5 volume of 100% ethanol at −20°C overnight. DNA was centrifuged at 3,500 rpm at 4°C for 30 min and washed three times with 70% ethanol, then resuspended in TLE. DNA concentration was measured using Quant-iT PicoGreen dsDNA Assay Kit (Invitrogen) according to manufacturer's instructions.

Biotin was removed from non-ligated restriction fragments in 100 μL reactions containing 5 μg of DNA, 10 μg of BSA, 1x NEB Buffer 2, 0.1 mM dATP and 15U of T4 DNA polymerase (New England Biolabs) and incubated at 20°C for 4 h. The enzymatic reaction was stopped by addition of 2 μL of 0.5M EDTA pH 8.0 and DNA purified by phenol-chloroform extraction followed by ethanol precipitation.

DNA Pellets were resuspended in 130 μL of nuclease-free water per 10 μg of material and sheared by sonication using an E220 ultrasonicator (Covaris) according to manufacturer's instructions using the following settings: 10% duty factor, 140W peak incident power, 200 cycles per burst, 55 s treatment time. Sheared ends were repaired by adding 6.5U of Klenow (New England Biolabs), 65U of T4 DNA polynucleotide kinase (New England Biolabs), 19.5U of T4 DNA polymerase (New England Biolabs), 1x T4 ligase buffer (Invitrogen) and 0.25 mM each of dATP, dCTP, dGTP and dTTP in 170 μL reactions and incubated at RT for 30 min. The DNA was purified using a PCR Purification Kit (Qiagen) according to manufacturer's instructions and eluted from the columns with 30 μL of TLE (Tris Low EDTA: 10 mM Tris-HCl pH 8.0, 0.1 mM EDTA) twice to give final volume of 60 μL per every 10 μg of material.

3′ A overhangs were added to the repaired ends of DNA fragments in 50 μL reactions containing 10 μg of Hi-C material, 1x NEB Buffer 2, 0.25 mM dATP and 17.5U of Klenow fragment 3′→5′ exo- (New England Biolabs) and incubated at 37°C for 30 min, followed by enzyme inactivation at 65°C for 20 min.

Fragments 200 bp-600 bp long were selected by double-sided SPRI bead selection using Ampure XP beads (Beckman Coulter) according to the manufacturer's instructions. First selection was performed with ×0.6 volumes (60 μL) of beads to remove high molecular weight DNA fragments. The unbound fraction was used to perform the second selection with ×0.9 volumes of beads (additional 30 μL of beads). Bound DNA of desired sizes was eluted from the beads with 50 μL of TLE. All DNA from one biological replicate was pooled at this stage and DNA concentration was measured using Quant-iT PicoGreen dsDNA Assay Kit (Incitrogen) according to the manufacturer's instructions. The yield was between 10 and 30 μg per library.

Fragments containing biotinylated ligation junctions were pulled-down using streptavidin-coated beads. The libraries were divided into 5 μg aliquots and mixed with 150 μL of Dynabeads MyOne Streptavidin C1 beads (Life Technologies) and processed according to the manufacturer's instructions. The binding step was performed with beads suspended in 300 μL of 2x binding and wash buffer

(5 mM Tris-HCl pH = 8, 0.5 mM EDTA, 1M NaCl) and 300 μL of Hi-C material in TLE. Beads were finally washed with 200 μL of 2x T4 ligase buffer and then resuspended in 50 μL 1x T4 ligase buffer.

Paired-end sequencing adapters TruPE (Table S6A) were annealed together to produce double-stranded adapters by mixing 15 μL of both adapters (each at 100 μM) with 70 μL of nuclease-free water and heated to 90°C for 5 min followed by 15 min at 70°C and then cooled to RT on the bench. These are non-standard short adapters and do not contain barcodes. The barcodes were added later, during the Capture Hi-C library generation.

4 μL of annealed adapters and 1400U of T4 DNA ligase (New England Biolabs) were added to the streptavidin bead suspension containing biotinylated Hi-C material and incubated at RT for 2 h. Beads with Hi-C material were recovered using a magnetic separator, washed twice with 400 μL of wash buffer (5 mM Tris-HCl pH = 8, 0.5 mM EDTA, 1M NaCl, 0.05% Tween), once with 200 μL of wash buffer without Tween, once with 200 μL of 1x NEB Buffer 2, once with 60 μL of 1x NEB Buffer 2 and resuspended in 40 μL of 1x NEB Buffer 2. Aliquots from the same biological replicate were pooled.

Test PCR reactions were set up to determine the optimal number of cycles to amplify the Hi-C libraries. 2.5 μL of bead suspension were used as template with 0.3 μM each TruPE PCR primer 1.0.33 and TruPE PCR primer 2.0.33 (Table S6A), 1x Phusion Buffer, 0.6U of Phusion polymerase, and 0.25 mM each of dATP, dCTP, dGTP and dTTP in 25 μL reactions. An initial denaturation step of 98°C for 30 s was followed by a varying number of cycles (typically 7, 9 and 12) of 98°C for 10 s, 65°C for 30 s and 72°C for 30 s, followed by final extension for 7 min at 72°C.

The remaining bead-bound Hi-C material was split into 2.5 μL aliquots for use as template in PCR reactions as described above with the appropriate number of cycles. After library amplification, beads were immobilized on a magnetic separator and the supernatants containing the amplified library from the same biological replicate were pooled. DNA was purified and PCR primers removed using ×0.9 volumes of Ampure XP beads (Beckman Coulter) following the manufacturer's instructions and eluted in 100 μL of TLE.

Hi-C libraries were analyzed using Bioanalyzer High-Sensitivity DNA Assay (Agilent) by the Next Generation Sequencing Facility at the Babraham Institute.

Capture HiC

Capture baits

Baits were designed to cover all three immunoglobulin loci as well as several selected genes important in B cell development and control regions. The *Igh* locus baits were made using 21 overlapping BACs and covered all HindIII fragment ends in the 3.1 Mb genomic region chr12:113074084-116172457, which encompasses the 2.8 Mb *Igh* locus chr12:113221856-116010765 and an additional 148 kb downstream and 162 kb upstream.

BAC DNA isolation. BACs (BACPAC Resources and Source BioScience) used for Capture Hi-C bait generation (Table S6B).

A single bacterial colony was l grown in 4 mL of LB with 25 mg/L chloramphenicol at 37°C for 8 h while shaking at 200 rpm. The culture was transferred into a flask containing 200 mL of LB with 25 mg/L chloramphenicol and grown at 37°C overnight at 200 rpm. BAC DNA was extracted from bacterial cultures using NucleoBond BAC 100 kit (Macherey-Nagel) according to the manufacturer's instructions. Purified BAC DNA was resuspended in elution buffer (Qiagen) and heated while shaking to ensure the DNA was solubilized. The BR dsDNA Qubit assay (ThermoFisher) was used to determine DNA concentration.

Baits generation. Typically 5 BACS were processed simultaneously. 5 μg each BAC DNA was digested with 2U/μL (40U) of HindIII, overnight at 37°C, followed by additional 1U/μL for 2 h, purified by phenol/chloroform/isoamylalcohol (Sigma) extraction and then chloroform extraction and ethanol precipitated at −80°C. The DNA was collected by centrifugation at 14000 rpm for 30 min at 4°C. The pellet was washed with 70% ethanol and resuspended in 25 μL 10 mM Tris pH 7.5. DNA concentration was determined by NanoDrop spectrophotometer.

Adapters containing T7 promoter sequence were produced by annealing two oligonucleotides (Table S6A) as described above for TruPE adapters. Adapters were then ligated to digested BAC DNA in 150 μL reactions with 4800U of T4 DNA ligase (New England Biolabs) and annealed T7 promoters (20 μM) at 3.1 μL per 10 μg DNA. Reactions were incubated at 16°C overnight and then inactivated at 65°C for 10 min.

To generate 200 bp bait fragments, the BAC DNA mix was sheared by sonication using an E220 ultrasonicator (Covaris) with the following parameters: 10% duty factor, 175 W peak incident power, 200 cycles per burst, 180 s treatment time. Sheared ends were repaired as follows: Two end repair reactions were set up for each set of baits with the remaining DNA split between the two reactions. Each 160 μL reaction contained 60 U T4 polynucleotide kinase (New England BioLabs), 18 U T4 DNA polymerase (New England BioLabs), 6.5 U DNA Polymerase I, Large (Klenow) Fragment (New England BioLabs) and 0.16 mM dTNPs, in 1x T4 ligase buffer (New England BioLabs). DNA was purified by QiaQuick PCR purification kit (Qiagen) in accordance with the manufacturer's instructions, and eluted in 50ul then two columns combined per reaction; 4 columns per set of baits. DNA was purified as described above for Hi-C material, and size-selected for 180 bp-300 bp fragments using ×0.7 and ×1.0 volumes of SPRI beads.

Biotinylated RNA baits were generated by using size-selected BAC DNA fragments as a template in 20 μL *in vitro* transcription reactions using T7 MegaScript kit (Life Technologies). Each reaction contained 1x reaction buffer, up to 1μg template DNA, 2.5 mM biotin-UTP (Roche), 3.75 mM rUTP, 5.63 mM each rATP, rCTP, rGTP and 2 μL of T7 RNA polymerase. Reactions were incubated at 37°C overnight. Template DNA was removed by incubation with 2U of TURBO DNase (Life Technologies) at 37°C for 15 min.

Biotinylated RNA baits were purified using Ambion MEGAclear kit (Life Technologies) according to the manufacturer's instructions. RNA yield was determined using NanoDrop spectrophotometer. The groups of RNA baits were combined to obtain an equimolar pool containing all the baits to be used in the capture.

The Capture Hi-C baits were sequenced as an RNAseq library to confirm *in vitro* transcription (Figures S1B–S1D).

Capture Hi-C procedure

500ng of Hi-C material were dried using a SpeedVac vacuum concentrator (Thermo Scientific) at 45°C until dry and resuspended in 3.5 μL of H_2O, then mixed with 2.5 μg of mouse Cot-1 DNA (Life Technologies), 2.5 μg of sheared salmon sperm DNA (Life Technologies) and 45.5 μM of each of four blocking oligos (Table S6A) in a 8.25 μL reaction and incubated at 95°C for 5 min followed by 5 min at 65°C. 13 μL of 2x hybridization buffer (11.15x SSPE, 11.15x Denhardts, 11.15 mM EDTA, 0.223% SDS) were pre-warmed to 65°C. 500 ng of biotinylated RNA baits in 5.5 μL volume were mixed with 30U of SUPERase-In (Life Technologies) and pre-warmed to 65°C for 2 min. Hi-C material was mixed with hybridization buffer and baits and incubated at 65°C for 24 h in a thermocycler with a heated lid.

60 μL of Dynabeads MyOne Streptavidin T1 beads (Life Technologies) were washed 3 times in binding buffer (1M NaCl, 10 mM Tris-HCl pH 7.5 and 1 mM EDTA) and resuspended in 200 μL binding buffer. Beads were combined with the hybridization reaction and incubated at RT for 30 min to bind fragments from the Hi-C library that hybridized to the biotinylated baits. Beads were recovered using a magnetic separator and washed once with 500 μL of 1x SSC/0.1% SDS for 15 min at RT, followed by three washes of 10 min each at 65°C with 500 μL of pre-warmed 0.1x SSC/0.1% SDS. Finally beads were quickly washed with 200 μL of 1x NEB Buffer 2 at RT and resuspended in 30 μL of NEB Buffer 2.

Test PCRs were performed to determine the optimal number of cycles as described above for Hi-C and the final PCR was performed to amplify the Capture Hi-C libraries. In this step, the PCR primers used added barcoded adapters to each library (Table S6A). Barcoded adapters were also added to pre-capture Hi-C libraries.

Fragments of desired length (200 bp-600 bp) were selected by double-sided size selection using Ampure XP beads (Beckman Coulter) according to the manufacturer's instructions using ×0.5 volumes of beads, followed by ×1.0 volume of beads. DNA of desired sizes was eluted from the beads with 20 μL of TLE. A further ×1.0 volume SPRI size selection was performed. Capture Hi-C libraries were analyzed using Bioanalyzer High-Sensitivity DNA Assay (Agilent) and KAPA Library Quantification Kit (KAPA Biosystems) to check for product size, library concentration and adapter incorporation.

Polymer modeling

We applied polymer modeling to deconvolve the Hi-C data and extract the cell-to-cell variability of genome folding at Igh locus.[63] We adopted a coarse-grained description of chromatin and used a beads-on-a-string representation of polymer where each monomer corresponds to 20 kb chromatin. The distance between consecutive beads is defined by the length *a* (arbitrary unit). Each bead can interact with any other bead through a spherical-well potential with interaction radius equal to 1.5a and hard-core radius of 0.6a. We optimized the pairwise interaction energies (depth of the spherical-well) between monomers to reproduce the experimental heatmap as previously described,[63] see Figure S4A. Using the optimal model, we extracted 5001 3D representative conformations and studied the folding properties of single conformations. The source code used to perform the simulations can be found here https://github.com/zhanyinx/Zenk_Zhan_et_al_Nature2021/tree/master/simulations/cis/Montegrappa-1.2.

3D DNA fluorescence *in situ* hybridization (FISH)

3D DNA FISH was carried out on FACS-sorted *ex vivo* $Rag^{-/-Mom}$ pro-B cells, Rag/81X pre-B cells and DP thymocytes using directly-labeled fluorescent BAC probes as previously described[121] with minor modifications. Briefly, 2 μg of BAC DNA were nick-translated, incorporated with aminoallyl-dUTP (Ambion) and labeled with an Alexa Fluor 555, 488 or 647 fluorescent dye. Cells were placed on Poly-L-lysine coated slides (Sigma Aldrich) by dropping a droplet of 200,000 cells in the middle of the slide. Cells were left for 3 min at RT to settle. Cells were fixed by submerging the slides in 4% paraformaldehyde (Sigma Aldrich) for exactly 10 min at RT. The fixation was quenched in 155 mM glycine, cells were permeabilized in 0.1% saponin/0.1% Triton in 1x PBS, washed in PBS and stored in 50% glycerol at −20°C for 7 days.

20ng of each fluorescent DNA FISH probe (two probes per slide) were ethanol precipitated with addition of 2 μg of Cot-1 mouse DNA (Invitrogen) and 9.7 μg of salmon sperm DNA (Sigma). The probe mix pellet was resuspended in 5 μL of formamide (Sigma Aldrich) and mixed with 5 μL of 20% dextran sulfate in 2x SSC.

The slides were taken out of 50% glycerol, washed in PBS and calibrated in 20% glycerol/1x PBS. Slides were washed twice in 1x PBS, incubated in 0.1M HCl for 30 min and washed in 1x PBS. Cells were permeabilized with 0.5% saponin/0.5% Triton/1x PBS for 30 min and washed twice in PBS. Slides were equilibrated in 50% formamide/2x SSC for at least 10 min, briefly washed in PBS to wash off excess formamide, pat dried, and 10 μL of precipitated probe mix was pipetted onto a coverslip, which was then inverted and placed onto the cells on the slide and sealed around with Fixogum rubber cement (Marabu). To hybridize the probe, the slide was heated on a hot plate at 78°C for exactly 2 min and incubated in a humid box in the dark at 37°C for at least 16 h. The rubber cement was peeled off and slides placed in 2x SSC until the coverslip slid off. To remove unspecifically bound probe the slide was incubated in 50% formamide/2x SSC at 45°C for 15 min, then in 0.2x SSC at 63°C for 15 min, followed by 5 min in 2xSSC at 45°C, 5 min in 2x SSC at RT and 5 min in PBS at RT. Cell nuclei were stained with DAPI (5 μg/mL in 2x SSC) (Invitrogen) for exactly 2 min and washed in PBS. For final fixation, slides were incubated in 3.7% formaldehyde/1x PBS for exactly 5 min, then quenched with 155 mM glycine for

at least 30 min and washed in PBS. A 22 × 50 mm coverslip with a drop of ProLong Diamond (Thermo Fisher) mounting medium was mounted onto the slide, sealed with nail varnish and stored at 4°C overnight.

Fluorescent signal acquisition using Metacyte and analysis in Metafer

Fluorescent signals were acquired by MetaSytems Metacyte imaging system using a Zeiss Axio Imager Z2 microscope. Metacyte imaged fields of view with multiple cells, capturing the fluorescent signals across 15 focal planes every 0.5 μm in all nuclei in view, averaging 5 nuclei per field and capturing a total of 300–800 fields. Fluorescent signals were acquired using wavelengths of 488 nm (green), 555 nm (red), 647 nm (far red) and 358 nm (DAPI).

Metafer software analyzed every captured nucleus by identifying the number of fluorescent signals and recording their position coordinates (analysis parameters for spot selection: absolute spot area 0.25 μm^2, max spot area 0.1 μm^2, min spot distance 0.1 μm^2, min spot intensity 30%). Nuclei with a number of spots other than two per color were rejected. Cartesian coordinates of each signal in 3D were exported from Metafer and distances between all signal pairs or trios were calculated. The shortest distances in each cell were taken for further analysis. We measured the diameters of all analyzed nuclei. Nuclear volumes in $Rag^{-/-}$ pro-B cells and Rag/81X pre-B cells were similar. The average volume was 60.3 μm^3 (assuming spherical shape). However, nuclear volumes in thymocytes were significantly smaller and volume adjustment of factor 1.0601699 has been applied to normalize spot distances in thymocytes.

The differences between $Rag^{-/-}$, Rag/81X and thymocytes in the number of nuclei with two closest fluorescent signals closer than 1 μm were analyzed using Fisher's exact test with Bonferroni correction for multiple testing. The differences between all distances separating two closest fluorescent signals in three cell types: $Rag^{-/-}$, Rag/81X and thymocytes; were analyzed using Kruskal-Wallis test with Dunn's correction for multiple testing.

To determine the probability of random association of two loci closer than 1 μm, we first determined that in a sphere with r = 1 μm, and therefore volume $v = 4/3\pi 1^3 = 4.187$ μm^3, two loci have 100% probability to be within 1 μm from each other or closer (when either copy of each locus in a diploid genome is considered). Thus, the probability of a random association of two loci from either allele within 1 μm radius in an average spherical nucleus of 60 μm^3 volume is p = 4.187/60 = 0.069. Therefore, if two loci associate within 1 μm radius in fewer than 6.9% of cells, this association could be random. The theoretical probability of two signals to be within 0.3 μm at random is 0.18% and within 0.5 μm is 0.87%. For three loci to simultaneously associate at random the probability is 6.9%*6.9% = 0.48%.

To label the *Igh* locus, a BAC probe RP23-109B20 over the D-J region (referred to as 'Igh(DJ)') was used in experiments in $Rag^{-/-}$ pro-B cells and thymocytes. In FISH experiments in Rag/81X pre-B cells a BAC probe RP23-70F21 in the V region (referred to as 'Igh(V)') was used, because the *Igh*(DJ) probe also detected the Vh81X transgene on chromosome 16 giving three fluorescent signals per nucleus. To check how this might impact our results, we tested the difference in distances measured between the *Igh* and *Ebf1*, *Foxo1* and *Runx1* using the Igh(V) probe compared to the distances measured using the *Igh*(DJ) probe in pro-B cells (Figures S7C and S7E). In Figure 7A we showed that the *trans* hits contact the Igh predominantly around the Eμ-D-J region and around 114.37–114.47 Mb in the V_H region. Whereas the *Igh*(V) probe detects sequences at 114.71–114.81 Mb and as might be expected, the comparison showed increased distances for *Igh*(V)+*Foxo1* and Igh(V)+*Runx1* compared to *Igh*(DJ)+*Foxo1* and *Igh*(DJ)+*Runx1* in pro-B cells. This means that the distances between the Eμ-J-D region and *Foxo1* as well as *Runx1* in Rag/81X pre-B cells might in fact be slightly shorter than the measurements detected using the *Igh*(V) probe, which would further strengthen the significance of our findings. Interestingly, for *Igh*(V)+*Ebf1* in pro-B cells there was an increase in very short distances (below 0.5 μm) (Figure S7C) and indeed, the *Ebf1* appears to preferentially interact with the *Igh* in the V_H region (Figure 7A). However, the overall number of distances below 1 μm for *Igh*(V)+*Ebf1* was equal for the *Igh*(DJ) and the *Igh*(V) probes. To label the *Igk* locus, the BAC probes RP24-179E20 and RP23-124O23 were used.

QUANTIFICATION AND STATISTICAL ANALYSIS

Hi-C and Capture Hi-C analysis

Hi-C and Capture Hi-C libraries were sequenced as 100 bp paired-end reads on Illumina HiSeq2500. The Hi-C User Pipeline (HiCUP, version 0.7.2)[122] was used to identify the Hi-C ligation junctions, trim the reads if they extended beyond the junction, map each of the paired reads separately to mm10 mouse reference genome using Bowtie2 (version 2.3.2),[119] filter out uninformative di-tags,[123] deduplicate and assess the *cis*:*trans* ratio in Hi-C and Capture Hi-C datasets (Table S1).

Capture Hi-C data from each of three sequencing runs was processed separately and di-tags belonging to the same biological replicate were combined and additionally de-duplicated. The capture efficiency was calculated as the proportion of di-tags in the final Capture Hi-C library with at least one end mapping to the baited regions. The level of enrichment of the baited sequences in Capture Hi-C datasets compared to Hi-C was calculated by dividing the number of reads per million covering baited regions in Capture Hi-C by the number of reads per million covering baited regions in Hi-C.

Generation of custom genome datasets

Capture Hi-C read pairs were filtered into two categories: (1) read pairs with both ends mapping to the baited regions (custom genome) and (2) read pairs with one end mapping to the baited regions (Figure S1E). Read pairs with none of the ends mapping to the baited regions were not considered in further analysis. Read pairs falling into category (1) had their coordinates shifted by the start of the baited sequence and custom genome dat files were produced to contain coordinates of custom 'mini chromosomes',

CellPress
OPEN ACCESS

whose starts and ends matched the starts and ends of the baited regions, using the Scribler script in HiCUP. Using the hicup2homer conversion script in HiCUP[122] the custom genome Capture Hi-C BAM files were converted to 'HiC summary' file format accepted by HOMER.[26]

Matrix normalization in HOMER

HOMER (version 4.7) software[26] was used to produce normalized interaction frequency matrices at 20 kb resolution for the Igh locus in Rag$^{-/-Mom}$ and Rag$^{-/-Bal}$ pro-B cells, and thymocytes. HOMER employs an iterative correction method for matrix balancing,[124] which corrects for biases inherent to Hi-C experiments. A background model at 20 kb resolution was generated and the *analyzeHiC* function was used to produce interaction matrices with or without distance correction (the -*simpleNorm* option was used to omit distance correction). Output matrices were visualized in Java TreeView.[125]

BACs used to generate baits for the Igh locus covered the region chr12:113074084-116172457, then the region chr12:113090000-116170000 was binned into 156 20 kb non-overlapping bins and used to generate HOMER matrices. The J, D and V genes, and the regulatory elements in the Igh locus falling into each 20 kb bin are listed in Table S1.

Subdomain calling in HiCSeg

Subdomains were defined on the normalized matrix in Rag1$^{-/-Mom}$ pro-B cells using the HiCseg package (v1.1) in R (v4.1.2). The distribution of the data was defined as Gaussian, and the "Dplus" model was used. The subdomain boundaries for 4–8 change-points were compared, and the boundary positions that were consistent between at least two of these were used to define the consensus subdomains. The analysis was repeated on the matrix with the bins corresponding to white lines (in which there was insufficient data to quantify interaction frequency) omitted, which confirmed that the consensus subdomains were identical and were thus not driven by these white lines.

Identification of significant inter-chromosomal interactions

Virtual 4C analysis has been performed by taking as a viewpoint all HindIII fragments in each region of interest baited by BACs (i.e., *Igh* - 3.1 Mb viewpoint covered by BACs, *Igk* - 3.5 Mb, *Igl* – 410 Mb, *Pax5* – 207 kb, *Foxo1* – 150 kb, etc.) The virtual 4Cs were carried out in Seqmonk v1.47.1 (https://www.bioinformatics.babraham.ac.uk/projects/seqmonk/) on raw Capture Hi-C data processed by HiCUP using read pairs with at least one end mapping to the baits (category (1) and (2), but with read pairs in *cis* removed). Other ends of reads mapping to each viewpoint were extracted and quantified in 500 kb bins genome-wide, creating 4C-like datasets. *trans* interactions yield far fewer reads than cis interactions, hence a lower resolution was required. The first 3 Mb of every chromosome's centromeric end was omitted, which is a standard approach when mapping NGS data to avoid these repetitive regions. Modified *Z* score analysis was performed on inter-chromosomal read counts to identify the highest outlier bins with *Z* score > 3.5, representing the most frequent interactions with the viewpoint. An interaction between the viewpoint and a 500 kb bin was considered significant if *Z* score was greater than 3.5 in both biological replicates. For 500 kb bins that satisfied this condition an average *Z* score was calculated. *Trans* interactions in Rag$^{-/-}$ pro-B cells were reported using Rag$^{-/-Mom}$ datasets. The significant hits were checked against blacklisted regions.[126] 4.5 Mb around the Vh81X transgene were also excluded from the *Z* score analysis. Whilst the integration site of the mouse Vh81X transgene in the genome of the Rag/81X mouse was previously unknown, we identified this site on chromosome 16 at a genomic location around 82,950,000–83,000,000 (Figure S5B). The virtual 4C from the *Igh* locus showed a strong 'interaction' peak on chr16 in this region, which was not present in virtual 4Cs from other viewpoints or in Rag$^{-/-}$ pro-B datasets. The transgene is absent from the genome assembly, therefore all reads experimentally coming from the transgene were mapped to the endogenous Igh locus, whereas their other ends interacting in cis were mapped to the transgene's surrounding sequences on chromosome 16, which ultimately allowed for its identification.

Virtual 4Cs from six of the top 14 *Igh trans* hits in Rag$^{-/-}$ pro-B cells were performed on raw reads as reciprocal analysis to identify the most frequently contacted parts of the Igh locus at 40 kb resolution (Figure 7A). *Runx1*, *Ebf1*, and *Pax5* were baited. Others, including *Aff3*, *Cux1* and *Foxp1* were not baited, but can serve as viewpoints for detecting reciprocal interactions with baited regions such as the Igh (other end-to-bait).

Overlaps between interchromosomal interactions were visualized using Venn diagrams and UpSet plots.[127]

A/B compartment analysis

The genome was binned into 500 kb bins and the *runHiCpca.pl* function in HOMER was applied to Hi-C datasets (pre-capture) to identify the first principal component (PC1). A H3K4me3 ChIP-seq dataset (GSE80155) was used to determine the sign on the PC eigenvectors. Default parameters were used except -*active H3K4me3*.

Hierarchical clustering

Hierarchical clustering of *trans* interactions was performed using pheatmap package in R using the default distance function ('Euclidean'), the default clustering function ('complete') and dendrogram clustering was applied to both columns and rows.

Polymer modeling: Clustering of conformations

To investigate whether the V(D)J locus assumed different sub-classes of conformations, we used two measures of structure similarity, (1) the root mean squared difference in bead-to-bead distance for all beads in each structure and (2) overlap of significant bead-to-bead interactions between any two conformations (Figures S4B and S4C). By clustering conformations with similar structure, we showed that V(D)J locus does not fold into defined subclasses of similar conformations, but rather it is made of a large amount of heterogeneous structures, which may underpin the diversity of V(D)J recombination events.

Correlation with recombination frequency

The recombination frequency of V beads was determined by taking the average of recombination frequencies of all active V genes in each 20 kb bead as determined by VDJ-seq.[4] Active V genes were identified by a binomial test on their primary recombination read counts and then used as a binary attribute.[4] Spearman's correlation was calculated between V bead recombination frequency and V-D interaction frequency ($<1.5a$).

Center of mass

Center of mass (CM) of the whole modeled Igh structure and the V gene-only center of mass (CMv) were defined as an average of all bead coordinates (avg x, avg y, avg z).

Inter-chromosomal interaction network in Cytoscape

The output of *Z* score analysis was visualized as a putative *trans* interaction network in Cytoscape.[118] The node labels display genes of interest of putative relevance identified by considering GO terms from GOrilla,[117] by gene expression in pre-B cells according to nucRNA-seq, and by their roles in immune response and lymphocyte development as reported in literature. Some 500 kb bins did not contain any genes of interest and their nodes are unlabeled.

RNA-seq

Nuclear RNA-seq from $Rag^{-/-Mom}$ pro-B cells and Rag/81X pre-B cells was performed as previously described.[4] Reads were trimmed with Trim Galore (https://www.bioinformatics.babraham.ac.uk/projects/trim_galore/), mapped with HISAT2[116] and log2-transformed reads per million were quantified in 0.5 Mb bins using Seqmonk (https://www.bioinformatics.babraham.ac.uk/projects/seqmonk/).

Other datasets used

ChIP-seq datasets for CTCF (GSE47766)[112] and H3K4me3 (GSE80155)[4] were mapped with Bowtie and peaks were called using MACS2 in the narrow peak mode.[128] Quantitation of CTCF peaks was performed in Seqmonk using the base pair quantitation option for 20 kb bins across the Igh locus. H3K4me3 ChIP-seq was used in A and B compartment analysis as described above.

Cell Reports, Volume *42*

Supplemental information

Intra- and interchromosomal contact mapping reveals the *Igh* locus has extensive conformational heterogeneity and interacts with B-lineage genes

Olga Mielczarek, Carolyn H. Rogers, Yinxiu Zhan, Louise S. Matheson, Michael J.T. Stubbington, Stefan Schoenfelder, Daniel J. Bolland, Biola M. Javierre, Steven W. Wingett, Csilla Várnai, Anne Segonds-Pichon, Simon J. Conn, Felix Krueger, Simon Andrews, Peter Fraser, Luca Giorgetti, and Anne E. Corcoran

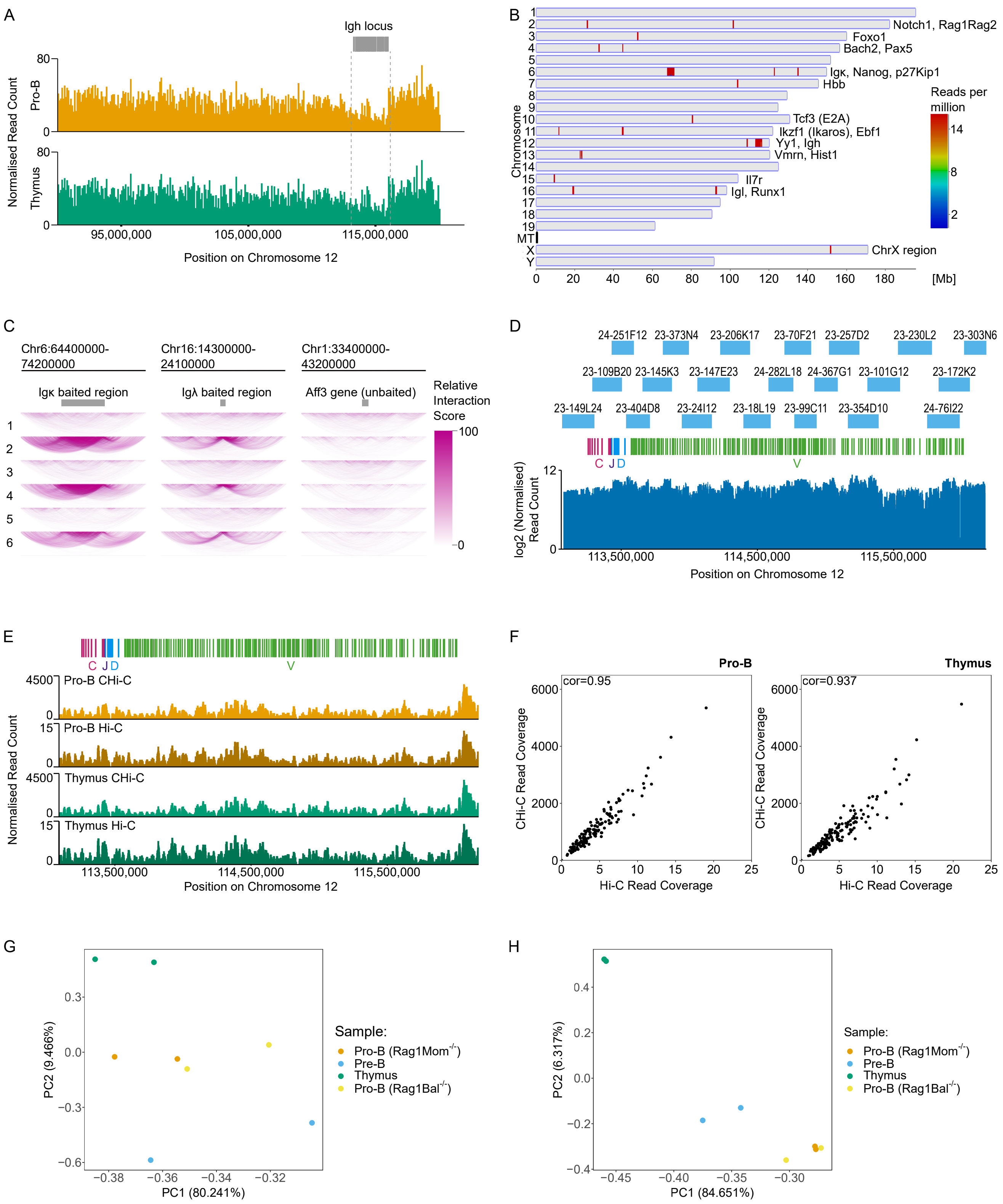
A
Igh locus
Normalised Read Count
Pro-B
Thymus
95,000,000
105,000,000
115,000,000
Position on Chromosome 12
B
Chromosome
Notch1, Rag1Rag2
Foxo1
Bach2, Pax5
Igκ, Nanog, p27Kip1
Hbb
Tcf3 (E2A)
Ikzf1 (Ikaros), Ebf1
Yy1, Igh
Vmrn, Hist1
Il7r
Igl, Runx1
ChrX region
Reads per million
[Mb]
C
Chr6:64400000-74200000
Chr16:14300000-24100000
Chr1:33400000-43200000
Igκ baited region
Igλ baited region
Aff3 gene (unbaited)
Relative Interaction Score
D
log2 (Normalised) Read Count
113,500,000
114,500,000
115,500,000
Position on Chromosome 12
E
Normalised Read Count
Pro-B CHi-C
Pro-B Hi-C
Thymus CHi-C
Thymus Hi-C
Position on Chromosome 12
F
Pro-B
Thymus
cor=0.95
cor=0.937
CHi-C Read Coverage
Hi-C Read Coverage
G
PC2 (9.466%)
PC1 (80.241%)
Sample:
Pro-B (Rag1Mom-/-)
Pre-B
Thymus
Pro-B (Rag1Bal-/-)
H
PC2 (6.317%)
PC1 (84.651%)

Figure S1. Enrichment of Hi-C material by bait pull-down. Related to Figure 1

(A) The Igh has lower read coverage in Hi-C than adjacent regions. Hi-C reads in the region chr12:90,000,000-end were quantitated in 100kb bins, normalised per million reads and biological replicates were averaged.

(B) The baits were validated by RNA-seq. Read count per million in 100bp bins is shown genome-wide.

(C) Interactions of the baited Igκ and Igλ loci and non-baited Aff3 locus in Hi-C and CHi-C datasets. 5 million randomly sampled reads from HiCUP-processed Hi-C and CHi-C datasets were quantified in 200kb bins and visualized in the WashU Epigenome Browser. Arcs show interactions. Arc colour indicates the number of interactions. One representative replicate is shown for each type of dataset: 1 – Hi-C Pro-B 1; 2 – CHi-C Pro-B 1; 3 – Hi-C Pre-B 1; 4 – CHi-C Pre-B 1; 5 – Hi-C Thymus 1; 6 – CHi-C Thymus 1.

(D) Validation of Igh baits by RNA-seq. Log2 read count per million over HindIII sites (-300bp to +300bp) for the Igh baited region (C) are shown. BAC positions are indicated by the light blue rectangles.

(E) CHi-C and Hi-C libraries exhibit the same read coverage pattern across the Igh locus. For pro-B and thymus samples, reads in Hi-C libraries and Scribler-processed CHi-C libraries were quantitated in 10kb bins with 1kb step, normalised per million reads. Biological replicates were averaged.

(F) CHi-C and Hi-C read coverage across the Igh locus is correlated. For pro-B and thymus samples, reads in Hi-C libraries and Scribler-processed CHi-C libraries were quantitated in 20kb windows over the Igh locus, normalised per million reads and biological replicates averaged. cor = spearman correlation coefficients.

(G) PCA shows reproducibility of biological replicates. Scribler-processed CHi-C datasets screened against a blacklist were processed in Homer to generate genome wide interaction matrices at 0.5Mb resolution, correcting for read coverage. The PCA examined normalised interaction frequencies across all replicates for all pairs of 0.5Mb bins genome wide, filtering out pairs of bins where the normalised interaction frequency was zero across all replicates.

(H) PCA shows that all pro-B cell replicates have similar Igh locus conformation.

Homer read coverage corrected interaction matrices at 20kb resolution were generated for the Igh baited region for all replicates. Values corresponding to 'white lines,' where normalised interaction frequency could not be computed, were filtered from the matrices. A PCA was performed to show normalised interaction frequencies across all replicates for all pairs of 20kb bins.

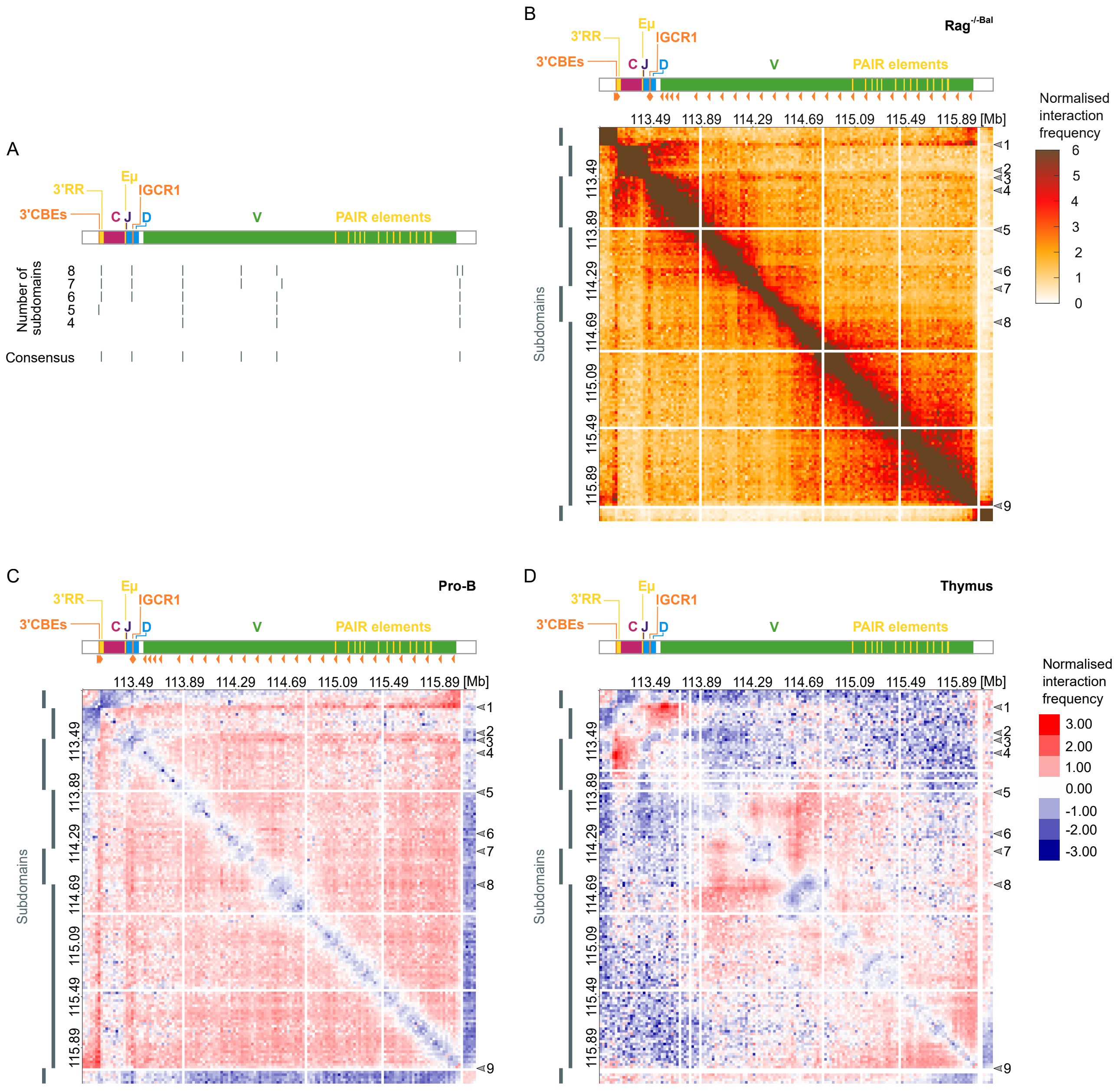

A
3'RR
Eμ
IGCR1
3'CBEs
C J D
V
PAIR elements
Number of subdomains
8
7
6
5
4
Consensus
B
Rag-/-Bal
Normalised interaction frequency
113.49 113.89 114.29 114.69 115.09 115.49 115.89 [Mb]
Subdomains
C
Pro-B
D
Thymus
3.00
2.00
1.00
0.00
-1.00
-2.00
-3.00

Figure S2. Interaction matrices of the Igh locus corrected for read coverage and distance. Related to Figure 2.

(A) Positions of subdomain boundaries determined by HiCseg when different numbers of subdomains are specified. Boundaries identified at least twice were taken as the consensus to define the subdomains.

(B) Read coverage corrected interaction matrix at 20kb resolution for the Igh baited region for $Rag^{-/-Bal}$ pro-B datasets.

(C) Read coverage and distance corrected interaction matrix at 20kb resolution for the Igh baited region for $Rag^{-/-Mom}$ pro-B and (D) thymus datasets.

All matrices were generated in Homer and biological replicates were averaged. White lines are bins with low read coverage excluded from analysis by Homer. Arrow 1 – 3'CBEs; arrow 2 – Eμ; arrow 3 – IGCR1; arrow 4 – most proximal V_H genes; arrow 5 – 5' of 7183 V_H gene family; arrow 6 – 5' of S107 V_H gene family; arrow 7 – 3' J606 V_H genes; arrow 8 – start of distal subdomain; arrow 9 - end of distal subdomain. The positions of subdomains in pro-B datasets determined using HiCseg are indicated by grey rectangles.

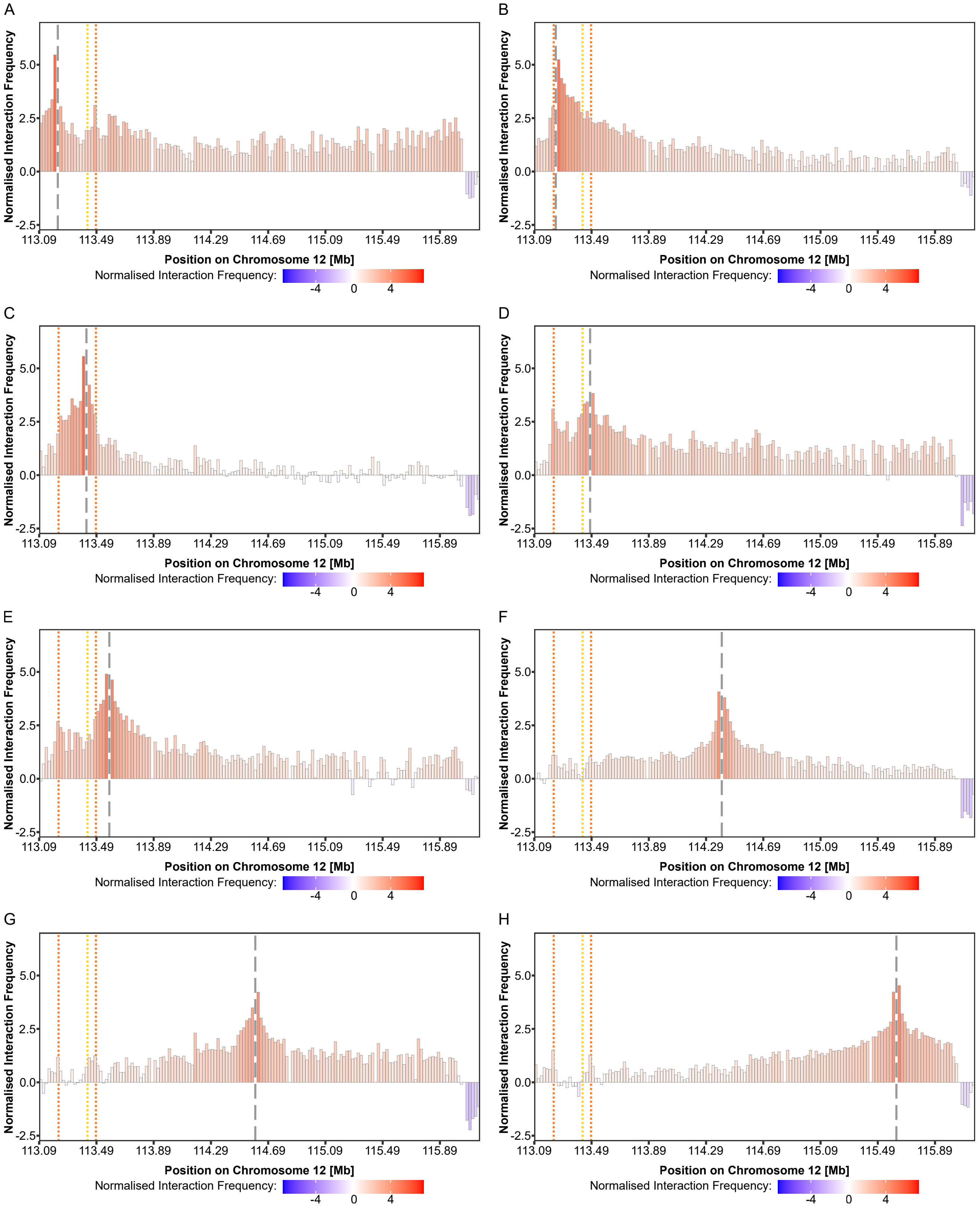

A
B
C
D
E
F
G
H
Normalised Interaction Frequency
5.0
2.5
0.0
-2.5
113.09
113.49
113.89
114.29
114.69
115.09
115.49
115.89
Position on Chromosome 12 [Mb]
Normalised Interaction Frequency:
-4
0
4

Figure S3. Virtual 4C interaction profiles of selected viewpoints in the Igh locus. Related to Figure 2.

Interaction counts were taken from the pro-B read coverage corrected interaction matrix in Figure 2A. The 20kb bin containing the viewpoint has been omitted for clarity and marked with a grey dashed line. Dotted lines indicate the positions of the 3'CBEs (left, orange), Eμ (middle, yellow) and IGCR1 (right, orange). (A) Interaction profile from 113.21-113.23Mb (bin containing 3'CBEs). (B) Interaction profile from 113.23-113.25Mb (bin containing 3'RR). (C) Interaction profile from 113.41-113.43Mb (bin containing Eμ). (D) Interaction profile from 113.47-113.49Mb (bin containing IGCR1). (E) Interaction profile from 113.57-113.59Mb (bin containing most 3' V_H gene). (F) Interaction profile from 114.39-114.41Mb (bin at 3' of J606 V_H gene family). (G) Interaction profile from 114.59-114.61Mb (bin preceding the distal domain). (H) Interaction profile from 115.61-115.63Mb (bin containing J558.71pg.172 and J558.72.173).

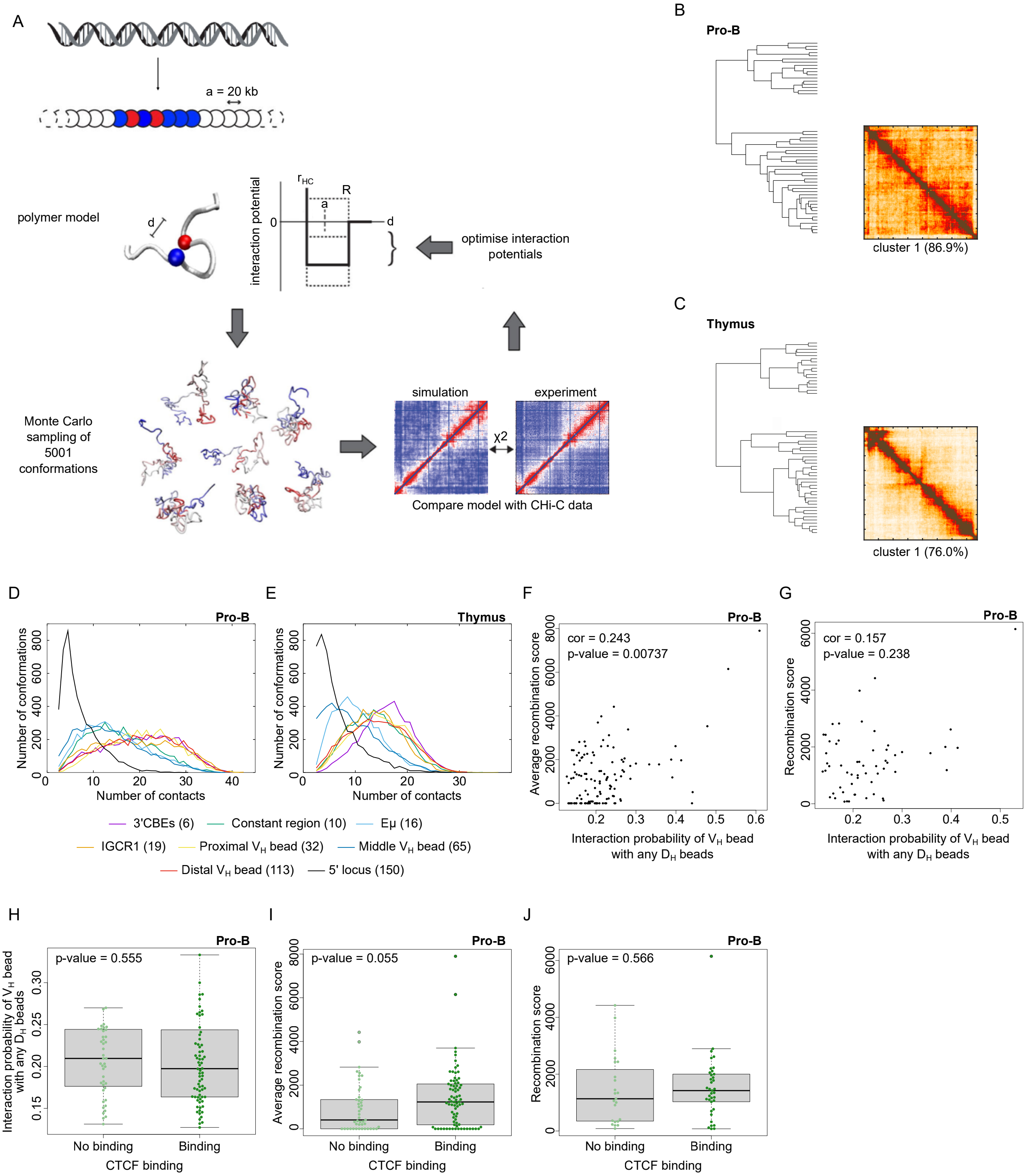

A
a = 20 kb
polymer model
d
interaction potential
r_{HC}
R
a
0
d
optimise interaction potentials
Monte Carlo sampling of 5001 conformations
simulation
experiment
χ2
Compare model with CHi-C data
B
Pro-B
cluster 1 (86.9%)
C
Thymus
cluster 1 (76.0%)
D
Pro-B
Number of conformations
Number of contacts
E
Thymus
Number of conformations
Number of contacts
3'CBEs (6)
Constant region (10)
Eμ (16)
IGCR1 (19)
Proximal V_H bead (32)
Middle V_H bead (65)
Distal V_H bead (113)
5' locus (150)
F
Pro-B
cor = 0.243
p-value = 0.00737
Average recombination score
Interaction probability of V_H bead with any D_H beads
G
Pro-B
cor = 0.157
p-value = 0.238
Recombination score
Interaction probability of V_H bead with any D_H beads
H
Pro-B
p-value = 0.555
Interaction probability of V_H bead with any D_H beads
No binding
Binding
CTCF binding
I
Pro-B
p-value = 0.055
Average recombination score
No binding
Binding
CTCF binding
J
Pro-B
p-value = 0.566
Recombination score
No binding
Binding
CTCF binding

Figure S4. Polymer modelling of Capture Hi-C. Related to Figure 3

(A) The chromatin fibre is treated as a string of beads, where adjacent beads correspond to consecutive 20kb of DNA sequence. There is no separation between beads (top). Schematic of the polymer model for structural deconvolution of CHi-C interaction frequencies (bottom). The model is constrained by the fixed distance between adjacent bead centres ($a=20kb$). Pairs of beads interact via short-range interaction potential (thick line) and each model gives a measure of pairwise distances between beads (d). $R=1.5a$ is a radius of a physical interaction, whereas $r_{HC}=0.6a$ is a hard-core repulsion radius. Monte Carlo sampling simulates the equilibrium ensemble of conformations and contact probabilities are compared to experimental CHi-C interaction frequency map using chi^2 (χ^2) as a dissimilarity score. Interaction potentials are iteratively optimised by further sampling (dotted lines) until simulated equilibrium ensemble converges with the experimental data.

(B and C) Single structures generated by the polymer model for pro-B (B) and thymus (C) datasets were clustered using differences in bead-to-bead contacts between each pair of conformations as a dissimilarity score.

(D and E) The distribution of the number of interacting partners for the 5001 conformations is shown for selected beads of the polymer model in pro-B cells (D) and thymus (E).

(F and G) There is weak correlation between V_H-D_H bead interaction probability and recombination score (provided by (Bolland et al., 2016)

(F) For all V_H beads, the correlation between the V_H-D_H bead interaction probability and the average recombination score of all V genes in that bead was determined. (G) For V_H beads containing a single active V_H gene, the correlation between the V_H-D_H interaction probability and the recombination score of the V_H gene was determined. cor = Spearman's correlation coefficient.

(H) CTCF binding does not impact V_H-D_H bead interactions. Boxplot shows the interaction probability of a given V_H bead with any D_H bead, where V_H beads are grouped depending on whether they bind CTCF or not. A Mann Whitney U test was performed.

(I and J) CTCF binding does not impact V_H-DJ_H recombination frequency. (I) The average recombination score of all V_H genes in a bead was compared between beads that do and do not bind CTCF. (J) For V_H beads containing a single active V_H gene, recombination score of that gene was compared between genes in beads that do and do not bind CTCF. A Mann Whitney U test was used in (I) and (J).

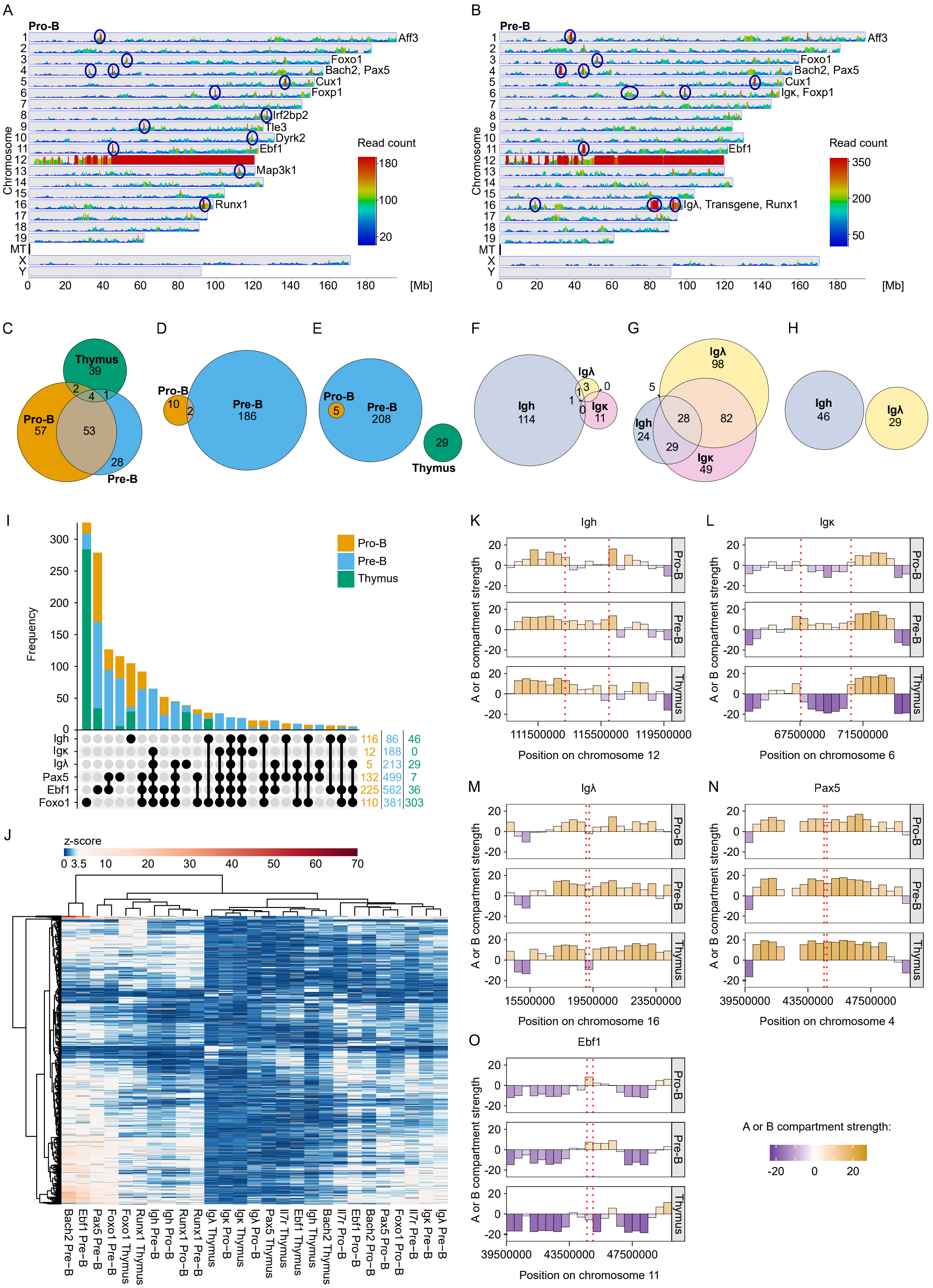

A
Pro-B
Aff3
Foxo1
Bach2, Pax5
Cux1
Foxp1
Irf2bp2
Tle3
Dyrk2
Ebf1
Map3k1
Runx1
Chromosome
Read count
[Mb]
B
Pre-B
Igκ, Foxp1
Igλ, Transgene, Runx1
C
Thymus
Pro-B
Pre-B
D
E
F
Igh
Igλ
Igκ
G
H
I
Frequency
Pro-B
Pre-B
Thymus
Igh
Igκ
Igλ
Pax5
Ebf1
Foxo1
J
z-score
K
Igh
Position on chromosome 12
L
Igκ
Position on chromosome 6
M
Igλ
Position on chromosome 16
N
Pax5
Position on chromosome 4
O
Ebf1
Position on chromosome 11
A or B compartment strength
A or B compartment strength:

Figure S5. Inter-chromosomal contacts of the Ig loci. Related to Figure 5.

(A and B) V4Cs from the Igh baited region were performed on Scribler-processed and blacklisted CHi-C datasets. Other ends were quantified in 0.5Mb bins genome wide and biological replicates were averaged. Genome wide views of interactions from the Igh are shown in (A) pro-B cells and (B) pre-B cells.

(C-E) Venn diagrams show shared significant (z-score>3.5) interchromosomal interactions from the (C) Igh viewpoint; (D) Igκ viewpoint; (E) Igλ viewpoint.

(F-H) The interchromosomal interactions are developmental stage specific and the Ig loci share interaction partners in pre-B cells. Venn diagrams show the shared significant (z-score>3.5) interactions in (F) Pro-B; (G) Pre-B; (G) Thymus.

(I) UpSet plot showing the number of unique and shared interchromosomal interactions between the three Ig loci, Pax5, Ebf1, and Foxo1 at each developmental stage. Numbers to the right indicate the total number of interchromosomal interactions for each viewpoint; only the top 25 intersection patterns (based on the sum across developmental stages) are shown. Note that whilst the bars are stacked to facilitate visualisation, interactions at a given developmental stage will overlap with those at other stages, as shown for the Ig loci in C-D.

(J) Hierarchical clustering shows similarities between interchromosomal interactions from the Ig loci and baited regions implicated in B cell function. 0.5Mb bins were selected for hierarchical clustering if they had a significant interaction with any of the viewpoints of interest (Igh, Igκ, Igλ, Bach2, Ebf1, Foxo1, Notch1, Pax5, Runx1 and Il7r) at any developmental stage. Clustering, based on average z-score values, was performed using the pheatmap package in R. The distance measure "Euclidean" and the clustering method "complete" were used.

(K-O) A or B compartment strength is shown in 0.5Mb bins for 10.5Mb regions centred on loci of interest: (K) Igh, (L) Igκ, (M) Igλ, (N) Pax5 and (O) Ebf1. Values >0 indicate A compartments and values <0 indicate B compartments. The position of the locus of interest is indicated by dotted red lines. Empty bins, seen in (M), arise due to low read coverage precluding calculationof PC1 values in Homer.

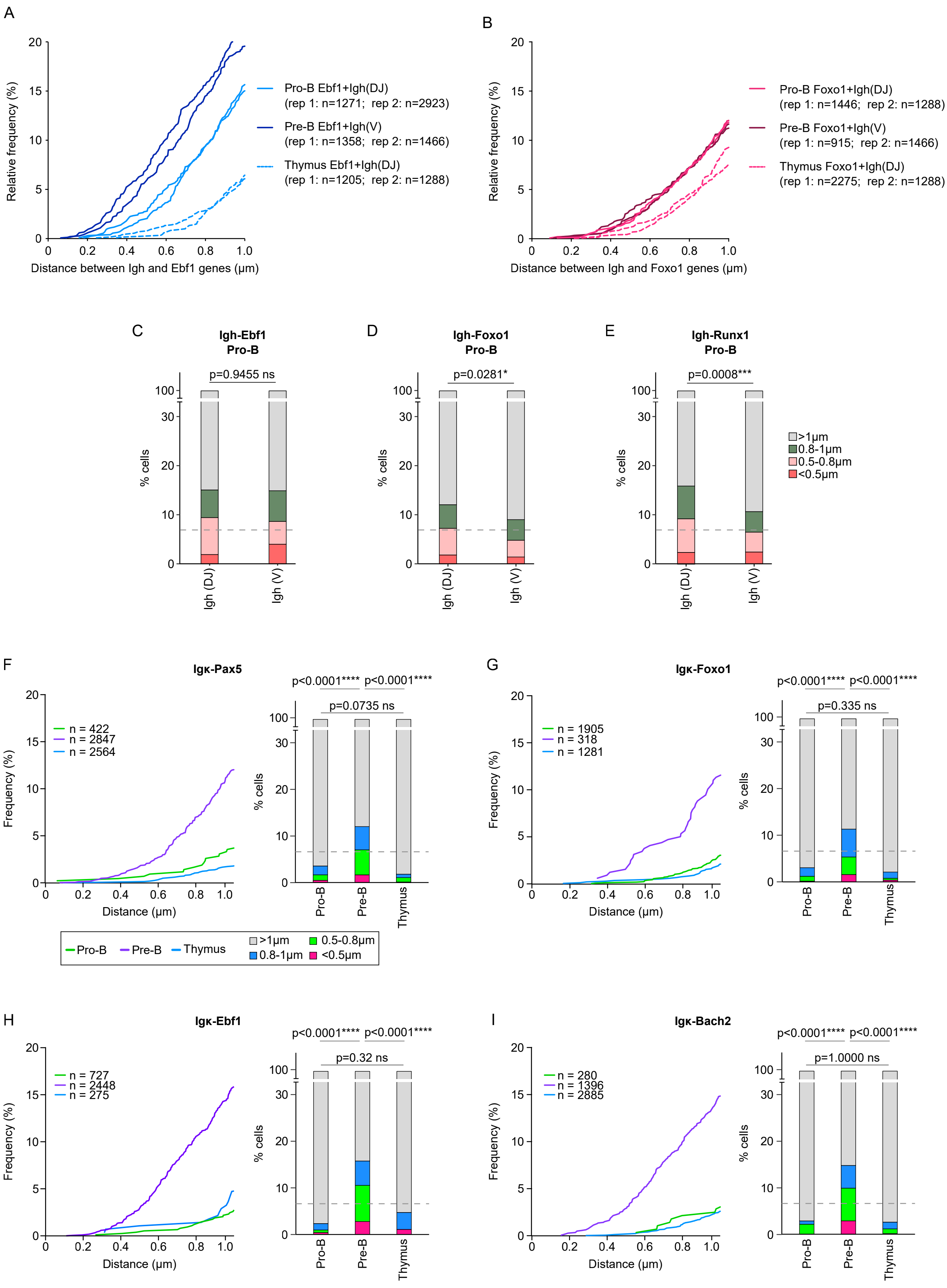

A
Relative frequency (%)
Distance between Igh and Ebf1 genes (μm)
Pro-B Ebf1+Igh(DJ)
(rep 1: n=1271; rep 2: n=2923)
Pre-B Ebf1+Igh(V)
(rep 1: n=1358; rep 2: n=1466)
Thymus Ebf1+Igh(DJ)
(rep 1: n=1205; rep 2: n=1288)
B
Relative frequency (%)
Distance between Igh and Foxo1 genes (μm)
Pro-B Foxo1+Igh(DJ)
(rep 1: n=1446; rep 2: n=1288)
Pre-B Foxo1+Igh(V)
(rep 1: n=915; rep 2: n=1466)
Thymus Foxo1+Igh(DJ)
(rep 1: n=2275; rep 2: n=1288)
C
Igh-Ebf1
Pro-B
p=0.9455 ns
% cells
Igh (DJ)
Igh (V)
D
Igh-Foxo1
Pro-B
p=0.0281*
% cells
Igh (DJ)
Igh (V)
E
Igh-Runx1
Pro-B
p=0.0008***
% cells
Igh (DJ)
Igh (V)
>1μm
0.8-1μm
0.5-0.8μm
<0.5μm
F
Igκ-Pax5
n = 422
n = 2847
n = 2564
Frequency (%)
Distance (μm)
p<0.0001**** p<0.0001****
p=0.0735 ns
% cells
Pro-B
Pre-B
Thymus
G
Igκ-Foxo1
n = 1905
n = 318
n = 1281
Frequency (%)
Distance (μm)
p<0.0001**** p<0.0001****
p=0.335 ns
% cells
Pro-B
Pre-B
Thymus
Pro-B
Pre-B
Thymus
>1μm
0.8-1μm
0.5-0.8μm
<0.5μm
H
Igκ-Ebf1
n = 727
n = 2448
n = 275
Frequency (%)
Distance (μm)
p<0.0001**** p<0.0001****
p=0.32 ns
% cells
Pro-B
Pre-B
Thymus
I
Igκ-Bach2
n = 280
n = 1396
n = 2885
Frequency (%)
Distance (μm)
p<0.0001**** p<0.0001****
p=1.0000 ns
% cells
Pro-B
Pre-B
Thymus

Figure S6. 3D FISH data is reproducible and validates Chi-C interchromosomal interactions of Igh and Igκ. Related to Figure 5.

Biological replicates of 3D FISH experiments for (A) Igh + Ebf1 and (B) Igh + Foxo1 were highly reproducible. The Igh (DJ) probe was used for pro-B and thymus, and the Igh (V) probe for pre-B. The closest signals in each cell were used. n = number of cells analysed per slide.

(C-E) The D-J region participates in interchomosomal interactions more frequently than the distal V region. Distances between the Igh and the locus of interest in pro-B cells (Ebf1 in (C); Foxo1 in (D); Runx1 in (E)) were compared when using the V FISH probe (BAC RP23-70F21) and the DJ FISH probe (BAC RP23-109B20) depicted in Figure 6. A Mann-Whitney test was performed. The distances <1μm are plotted in three categories: <0.5μm, 0.5-0.8μm and 0.8-1μm; Dashed line denotes the probability of a random interaction at a distance of <1μm of 6.9%.

(F-I) 3D FISH probing of the Igκ locus validates Igk interchromosomal interactions. Igκ with (F) Pax5, (G) Foxo1, (H) Ebf1 or (I) Bach2. Igκ probes RP24-179E20 and RP23-124O23 were labelled with Alexa Fluor 488 (green). Line graphs show cumulative distributions of distances <1μm between the Igκ locus and genes of interest in three cell types. The closest signal pairs in each cell were used. n=number of nuclei analysed. Bar graphs show distribution of distances grouped into four brackets (<0.5μm, 0.5-0.8μm, 0.8-1μm, >1μm). Dashed line denotes the probability of a random interaction at a distance of <1μm of 6.9%. P-values were calculated using the Fisher's exact test with Bonferroni correction.

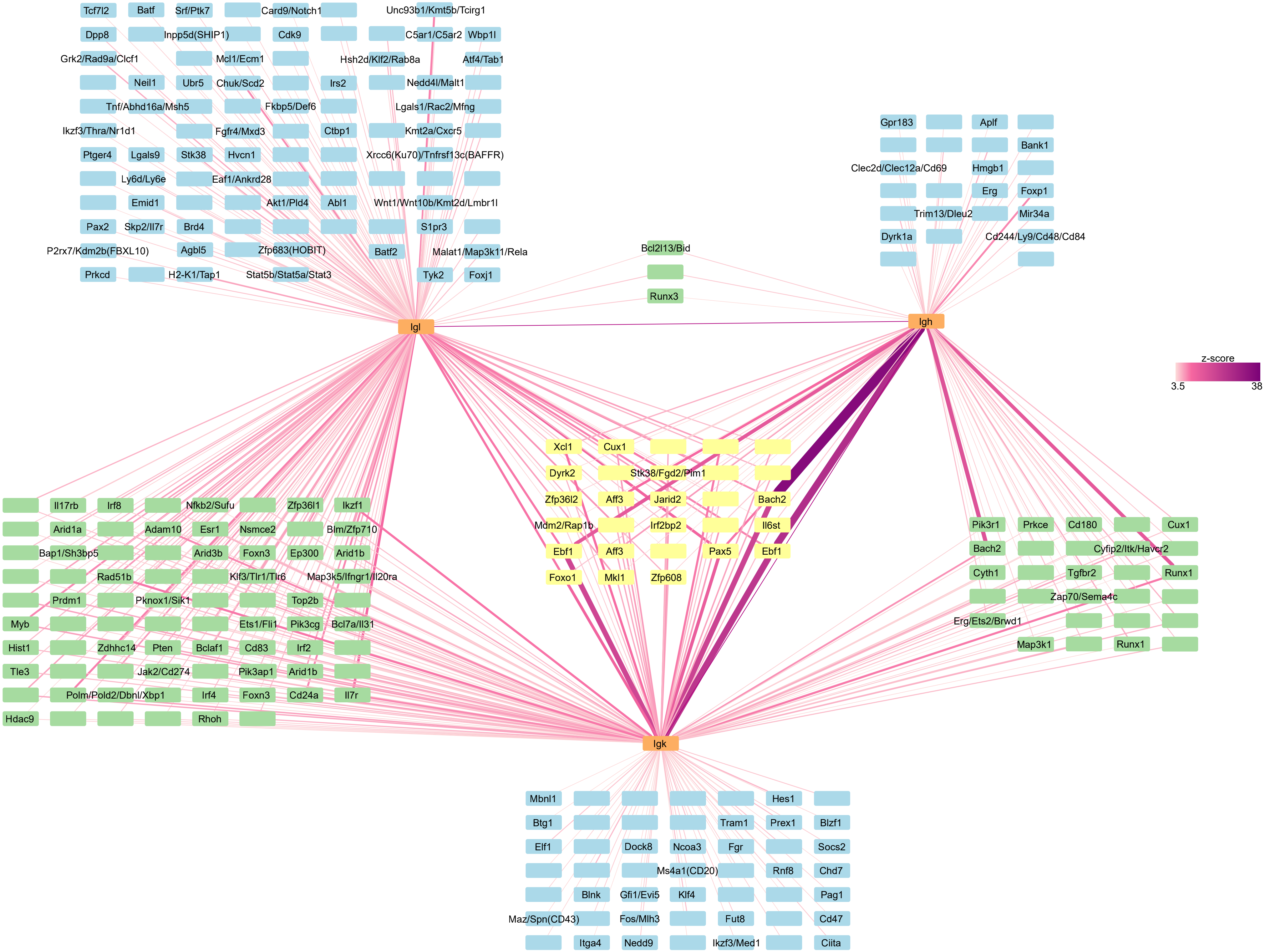

Tcf7l2
Batf
Srf/Ptk7
Card9/Notch1
Unc93b1/Kmt5b/Tcirg1
Dpp8
Inpp5d(SHIP1)
Cdk9
C5ar1/C5ar2
Wbp1l
Grk2/Rad9a/Clcf1
Mcl1/Ecm1
Hsh2d/Klf2/Rab8a
Atf4/Tab1
Neil1
Ubr5
Chuk/Scd2
Irs2
Nedd4l/Malt1
Tnf/Abhd16a/Msh5
Fkbp5/Def6
Lgals1/Rac2/Mfng
Ikzf3/Thra/Nr1d1
Fgfr4/Mxd3
Ctbp1
Kmt2a/Cxcr5
Ptger4
Lgals9
Stk38
Hvcn1
Xrcc6(Ku70)/Tnfrsf13c(BAFFR)
Ly6d/Ly6e
Eaf1/Ankrd28
Emid1
Akt1/Pld4
Abl1
Wnt1/Wnt10b/Kmt2d/Lmbr1l
Pax2
Skp2/Il7r
Brd4
S1pr3
P2rx7/Kdm2b(FBXL10)
Agbl5
Zfp683(HOBIT)
Batf2
Malat1/Map3k11/Rela
Prkcd
H2-K1/Tap1
Stat5b/Stat5a/Stat3
Tyk2
Foxj1
Gpr183
Aplf
Bank1
Clec2d/Clec12a/Cd69
Hmgb1
Erg
Foxp1
Trim13/Dleu2
Mir34a
Dyrk1a
Cd244/Ly9/Cd48/Cd84
Bcl2l13/Bid
Runx3
Igl
Igh
z-score
3.5
38
Xcl1
Cux1
Dyrk2
Stk38/Fgd2/Pim1
Zfp36l2
Aff3
Jarid2
Bach2
Mdm2/Rap1b
Irf2bp2
Il6st
Ebf1
Aff3
Pax5
Ebf1
Foxo1
Mkl1
Zfp608
Il17rb
Irf8
Nfkb2/Sufu
Zfp36l1
Ikzf1
Arid1a
Adam10
Esr1
Nsmce2
Blm/Zfp710
Bap1/Sh3bp5
Arid3b
Foxn3
Ep300
Arid1b
Rad51b
Klf3/Tlr1/Tlr6
Map3k5/Ifngr1/Il20ra
Prdm1
Pknox1/Sik1
Top2b
Myb
Ets1/Fli1
Pik3cg
Bcl7a/Il31
Hist1
Zdhhc14
Pten
Bclaf1
Cd83
Irf2
Tle3
Jak2/Cd274
Pik3ap1
Arid1b
Polm/Pold2/Dbnl/Xbp1
Irf4
Foxn3
Cd24a
Il7r
Hdac9
Rhoh
Pik3r1
Prkce
Cd180
Cux1
Bach2
Cyfip2/Itk/Havcr2
Cyth1
Tgfbr2
Runx1
Zap70/Sema4c
Erg/Ets2/Brwd1
Map3k1
Runx1
Igk
Mbnl1
Hes1
Btg1
Tram1
Prex1
Blzf1
Elf1
Dock8
Ncoa3
Fgr
Socs2
Ms4a1(CD20)
Rnf8
Chd7
Blnk
Gfi1/Evi5
Klf4
Pag1
Maz/Spn(CD43)
Fos/Mlh3
Fut8
Cd47
Itga4
Nedd9
Ikzf3/Med1
Ciita

Figure S7. Interchromosomal interaction network of the Ig loci in pre-B cells. Related to Figure 6.

The nodes correspond to 0.5Mb bins in which interchromosomal interactions were analysed. Bins contacted by all three Ig loci are indicated by yellow nodes, bins contacted by two Ig loci are indicated by green nodes, and bins contacted by only one Ig locus are indicated by blue nodes. The thickness and shade of the purple lines linking the nodes represent the frequency of the interaction (z-score value). Putative genes of interest are annotated for selected nodes.
